# Supplementary material for: An Educational Session for Medical Students Exploring Weight Bias in Clinical Care Through the Lens of Body Diversity
Source: MedEdPORTAL. 2023 Sep 5;19:11342. doi: 10.15766/mep_2374-8265.11342 (PMC10477274; doi:10.15766/mep_2374-8265.11342)
Supplement: Supplementary file 1 — Understanding Body Diversity.pptxAddressing Weight Bias in Clinical Care.pptxFacilitator Guide.docxStudent Guide.docxMaterials Checklist and Timeline.docxQuiz.docxEvaluation Survey.docx [file mep_2374-8265.11342-s001.zip › B. Addressing Weight Bias in Clinical Care.pptx]

## Slide 1
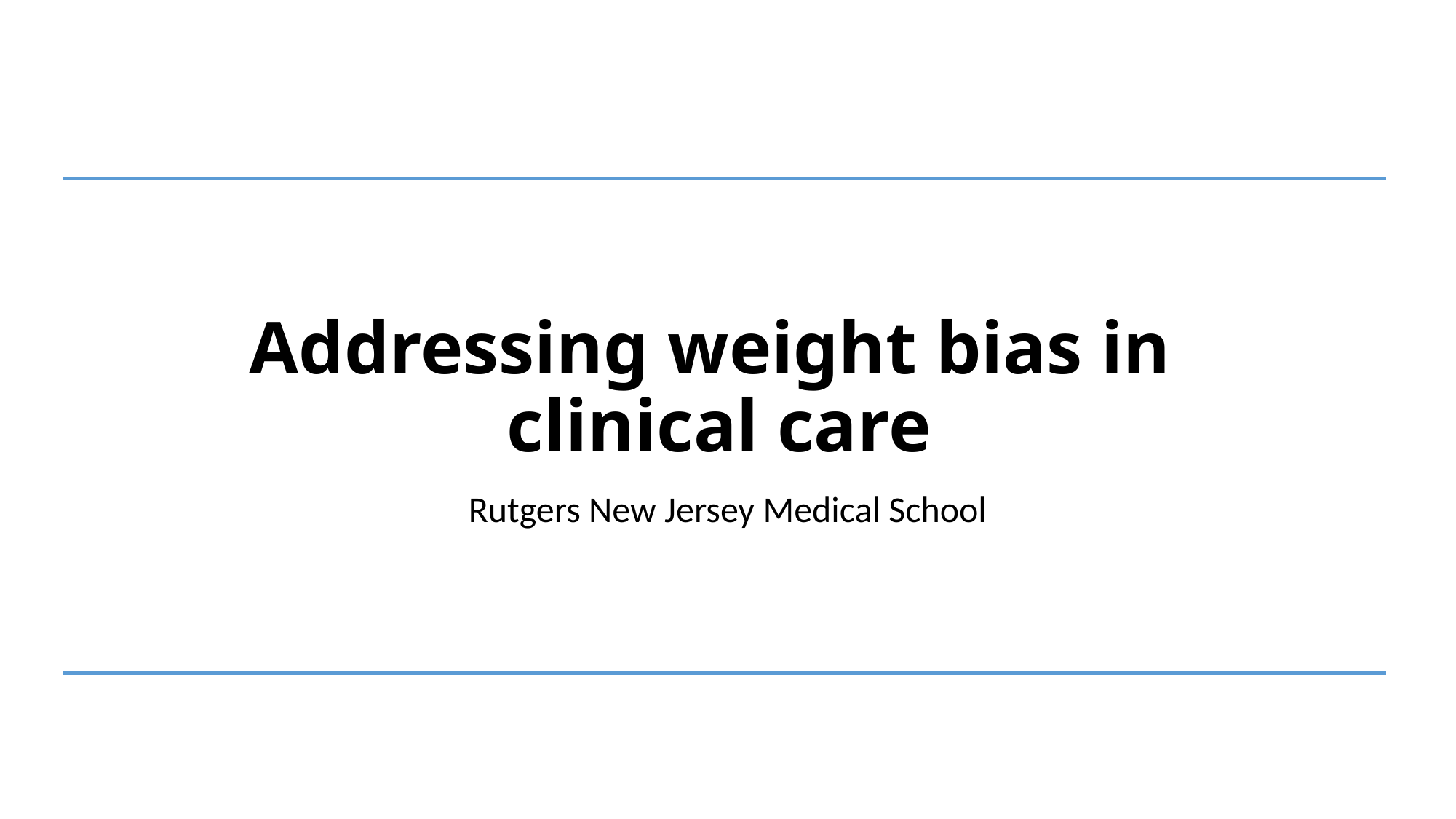

# Addressing weight bias in clinical care
Rutgers New Jersey Medical School

## Slide 2
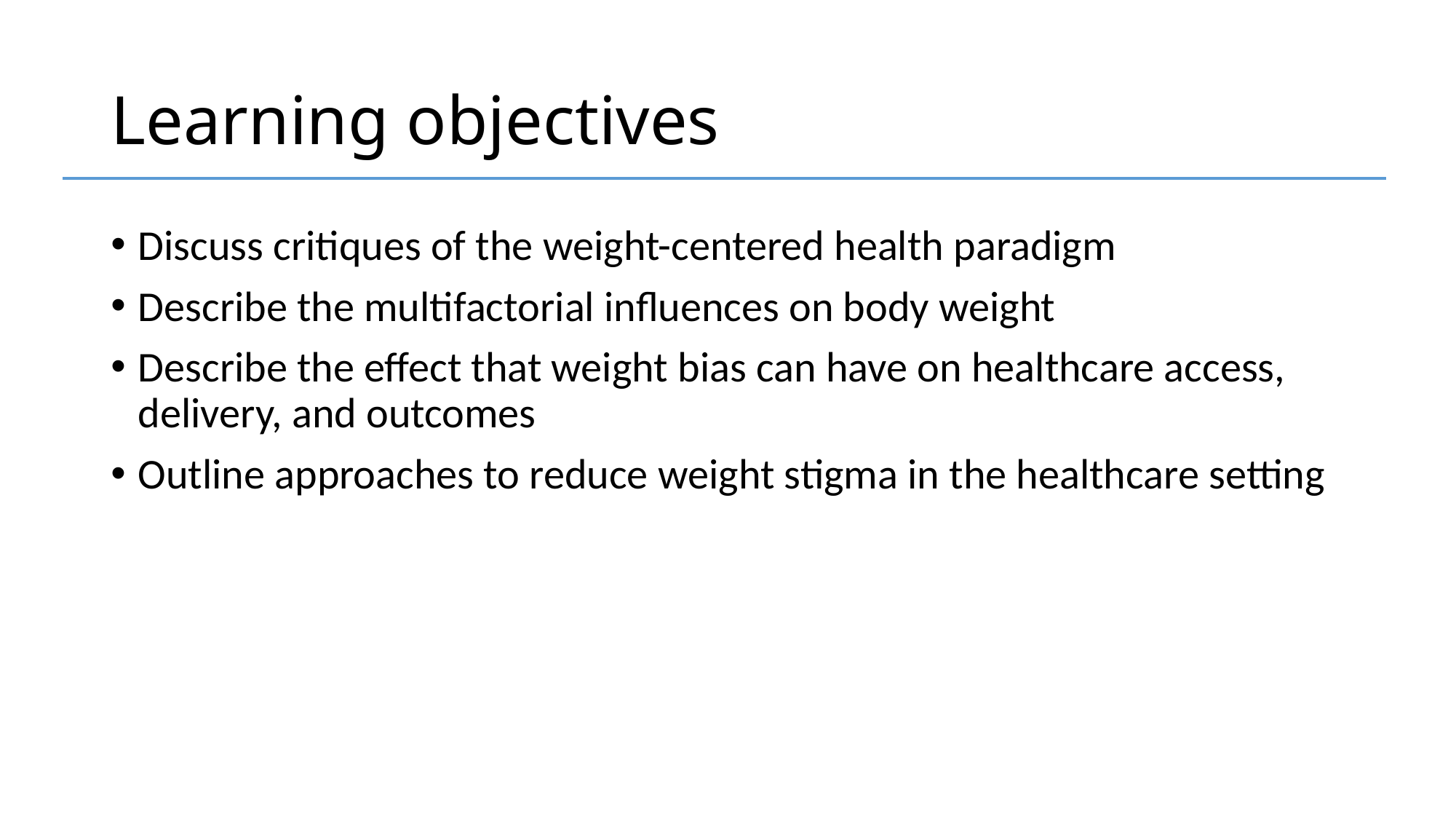

# Learning objectives
Discuss critiques of the weight-centered health paradigm
Describe the multifactorial influences on body weight
Describe the effect that weight bias can have on healthcare access, delivery, and outcomes
Outline approaches to reduce weight stigma in the healthcare setting

## Slide 3
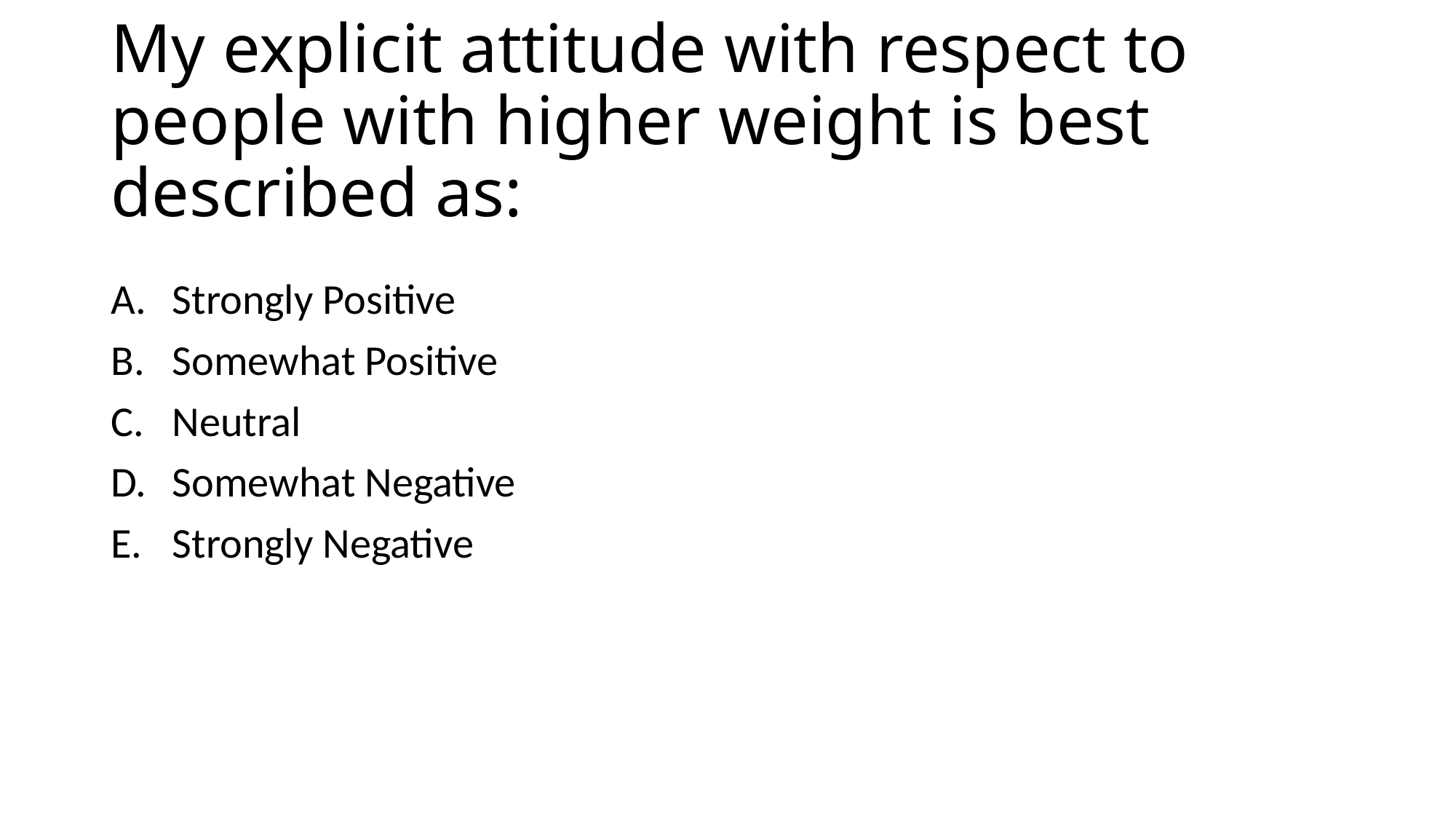

# My explicit attitude with respect to people with higher weight is best described as:
Strongly Positive
Somewhat Positive
Neutral
Somewhat Negative
Strongly Negative

## Slide 4
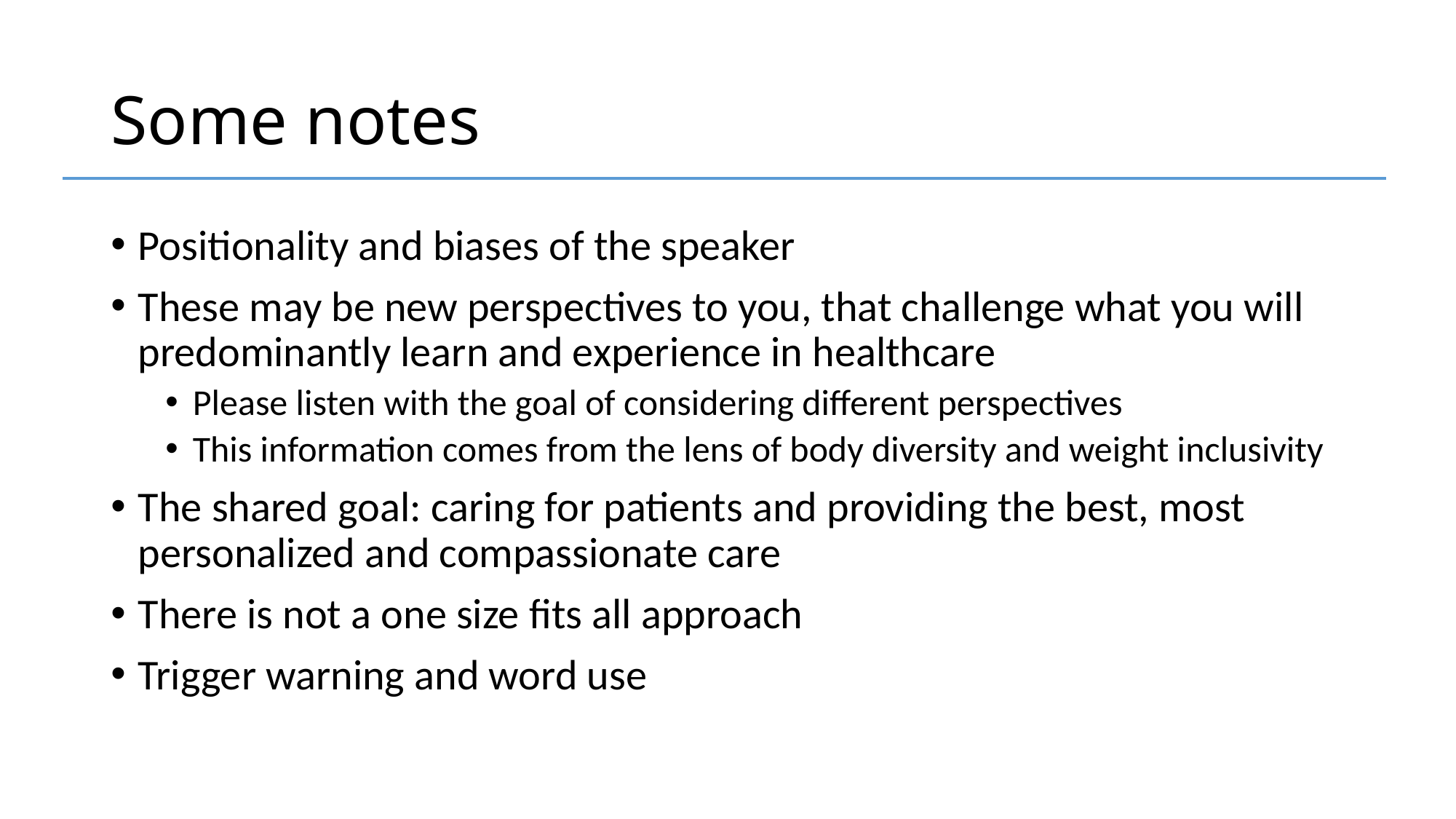

# Some notes
Positionality and biases of the speaker
These may be new perspectives to you, that challenge what you will predominantly learn and experience in healthcare
Please listen with the goal of considering different perspectives
This information comes from the lens of body diversity and weight inclusivity
The shared goal: caring for patients and providing the best, most personalized and compassionate care
There is not a one size fits all approach
Trigger warning and word use

## Slide 5
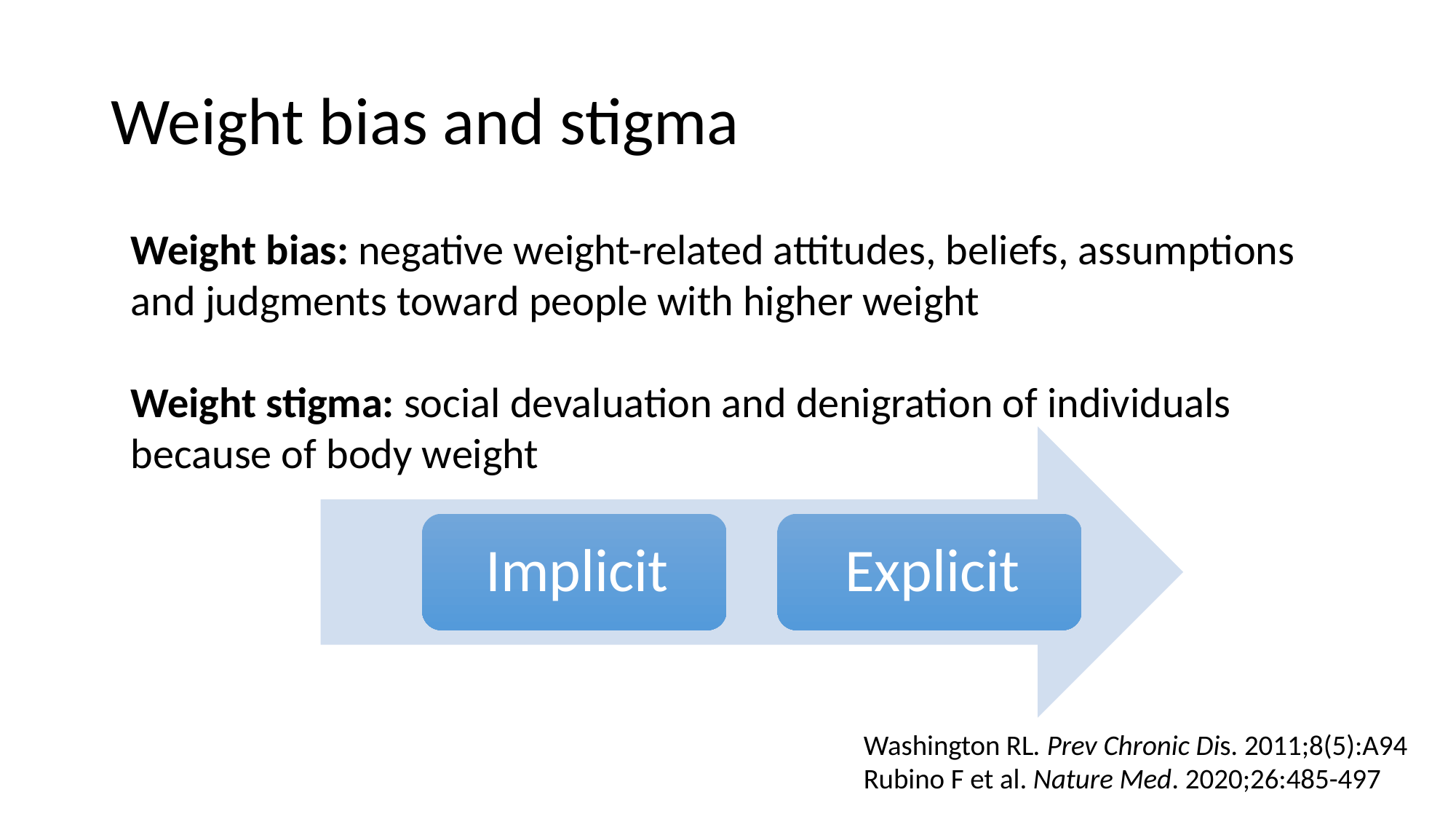

# Weight bias and stigma
Weight bias: negative weight-related attitudes, beliefs, assumptions and judgments toward people with higher weight
Weight stigma: social devaluation and denigration of individuals because of body weight
Washington RL. Prev Chronic Dis. 2011;8(5):A94
Rubino F et al. Nature Med. 2020;26:485-497

## Slide 6
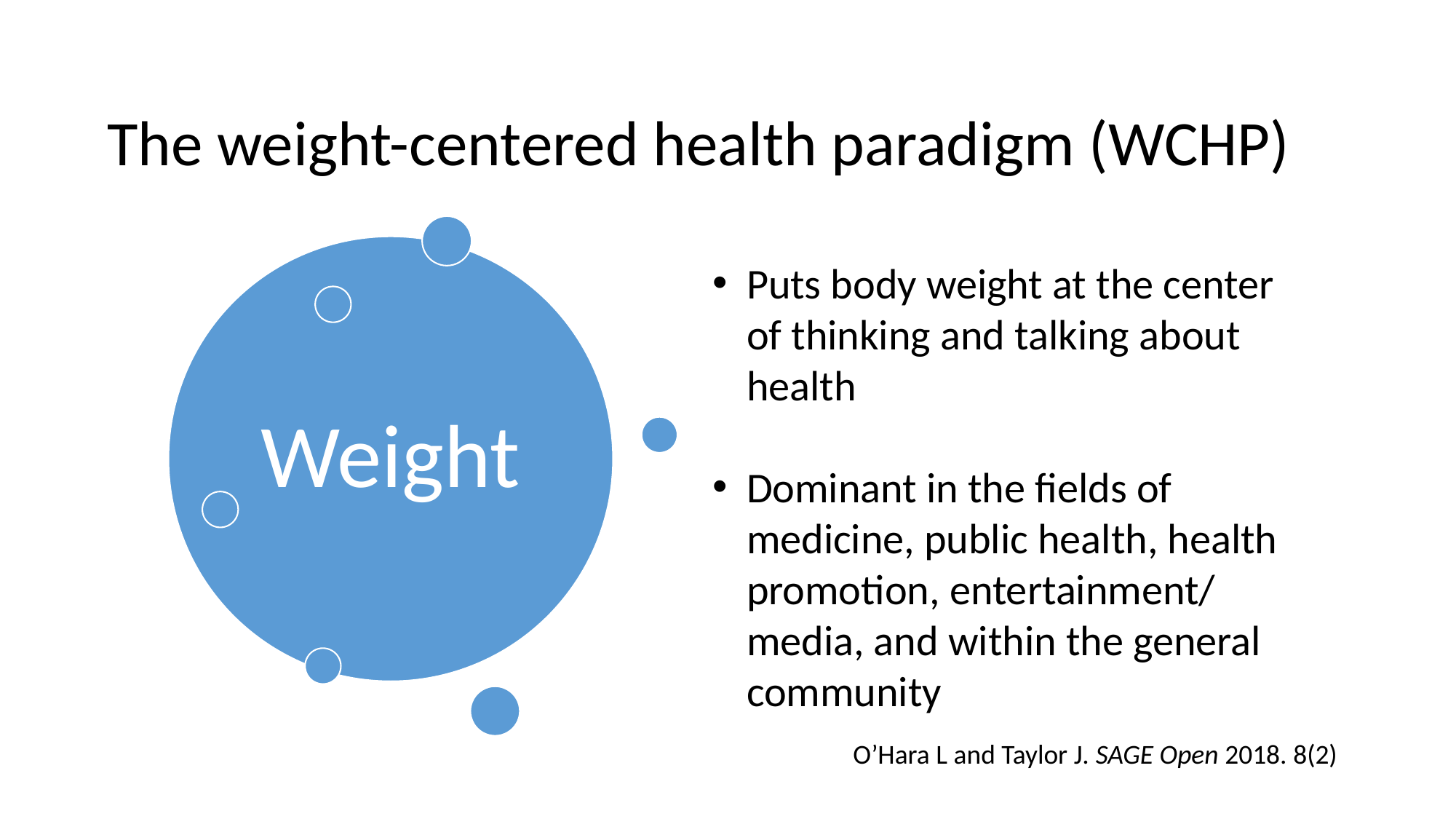

# The weight-centered health paradigm (WCHP)
Puts body weight at the center of thinking and talking about health
Dominant in the fields of medicine, public health, health promotion, entertainment/ media, and within the general community
O’Hara L and Taylor J. SAGE Open 2018. 8(2)

## Slide 7
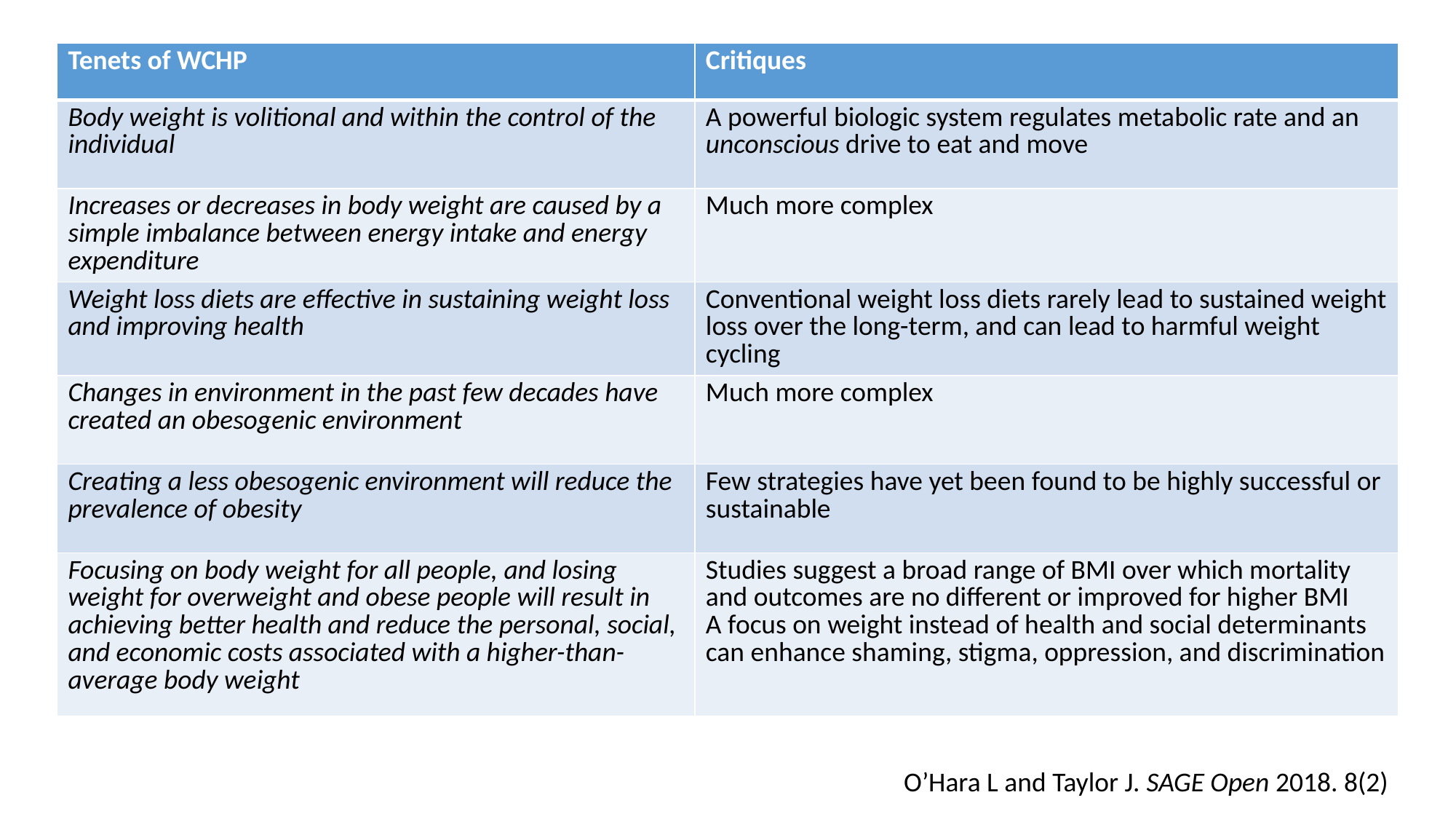

| Tenets of WCHP | Critiques |
| --- | --- |
| Body weight is volitional and within the control of the individual | A powerful biologic system regulates metabolic rate and an unconscious drive to eat and move |
| Increases or decreases in body weight are caused by a simple imbalance between energy intake and energy expenditure | Much more complex |
| Weight loss diets are effective in sustaining weight loss and improving health | Conventional weight loss diets rarely lead to sustained weight loss over the long-term, and can lead to harmful weight cycling |
| Changes in environment in the past few decades have created an obesogenic environment | Much more complex |
| Creating a less obesogenic environment will reduce the prevalence of obesity | Few strategies have yet been found to be highly successful or sustainable |
| Focusing on body weight for all people, and losing weight for overweight and obese people will result in achieving better health and reduce the personal, social, and economic costs associated with a higher-than-average body weight | Studies suggest a broad range of BMI over which mortality and outcomes are no different or improved for higher BMI A focus on weight instead of health and social determinants can enhance shaming, stigma, oppression, and discrimination |
O’Hara L and Taylor J. SAGE Open 2018. 8(2)

## Slide 8
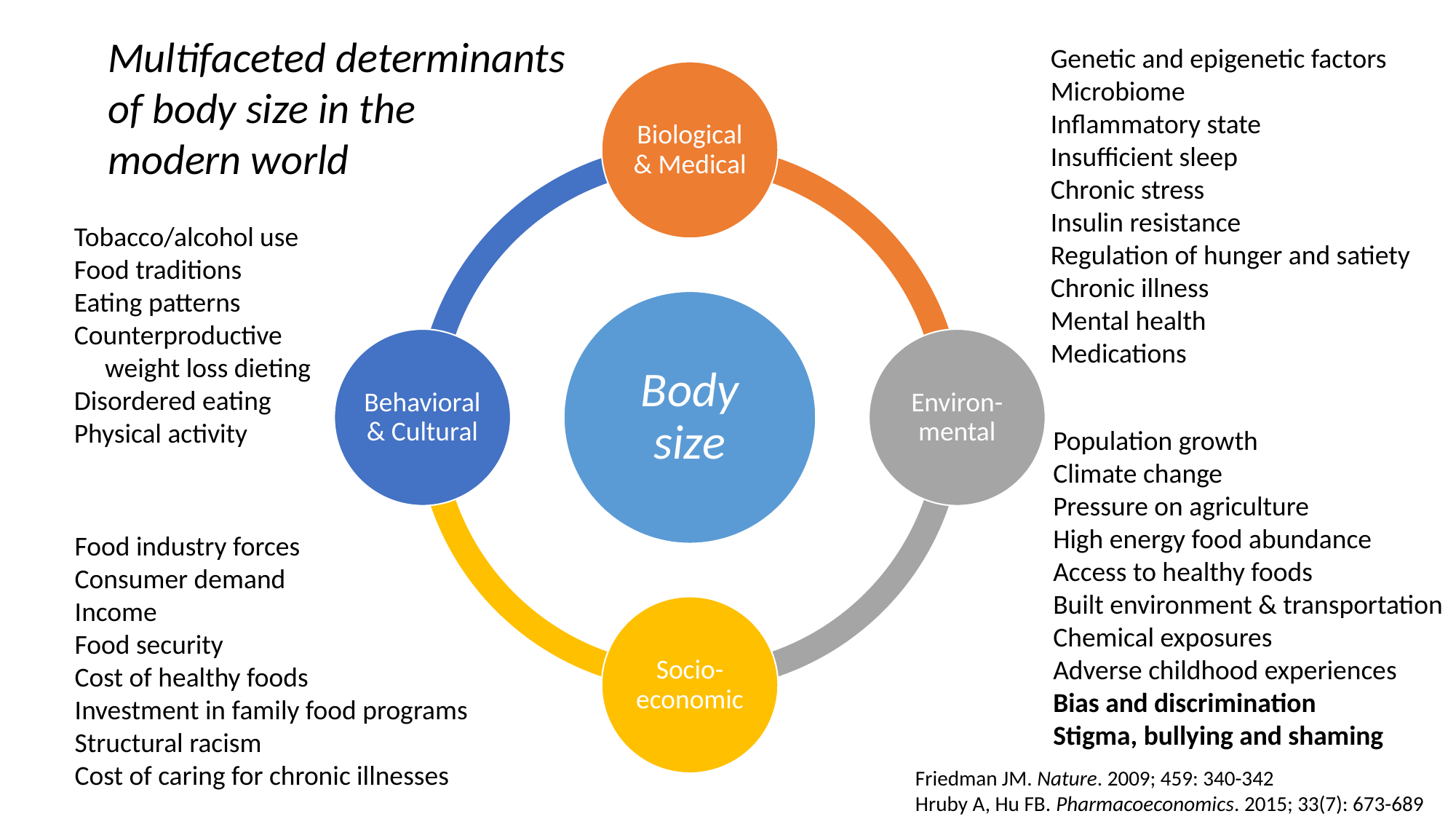

Multifaceted determinants
of body size in the
modern world
Genetic and epigenetic factors
Microbiome
Inflammatory state
Insufficient sleep
Chronic stress
Insulin resistance
Regulation of hunger and satiety
Chronic illness
Mental health
Medications
Tobacco/alcohol use
Food traditions
Eating patterns
Counterproductive
 weight loss dieting
Disordered eating
Physical activity
Population growth
Climate change
Pressure on agriculture
High energy food abundance
Access to healthy foods
Built environment & transportation
Chemical exposures
Adverse childhood experiences
Bias and discrimination
Stigma, bullying and shaming
Food industry forces
Consumer demand
Income
Food security
Cost of healthy foods
Investment in family food programs
Structural racism
Cost of caring for chronic illnesses
Friedman JM. Nature. 2009; 459: 340-342
Hruby A, Hu FB. Pharmacoeconomics. 2015; 33(7): 673-689

## Slide 9
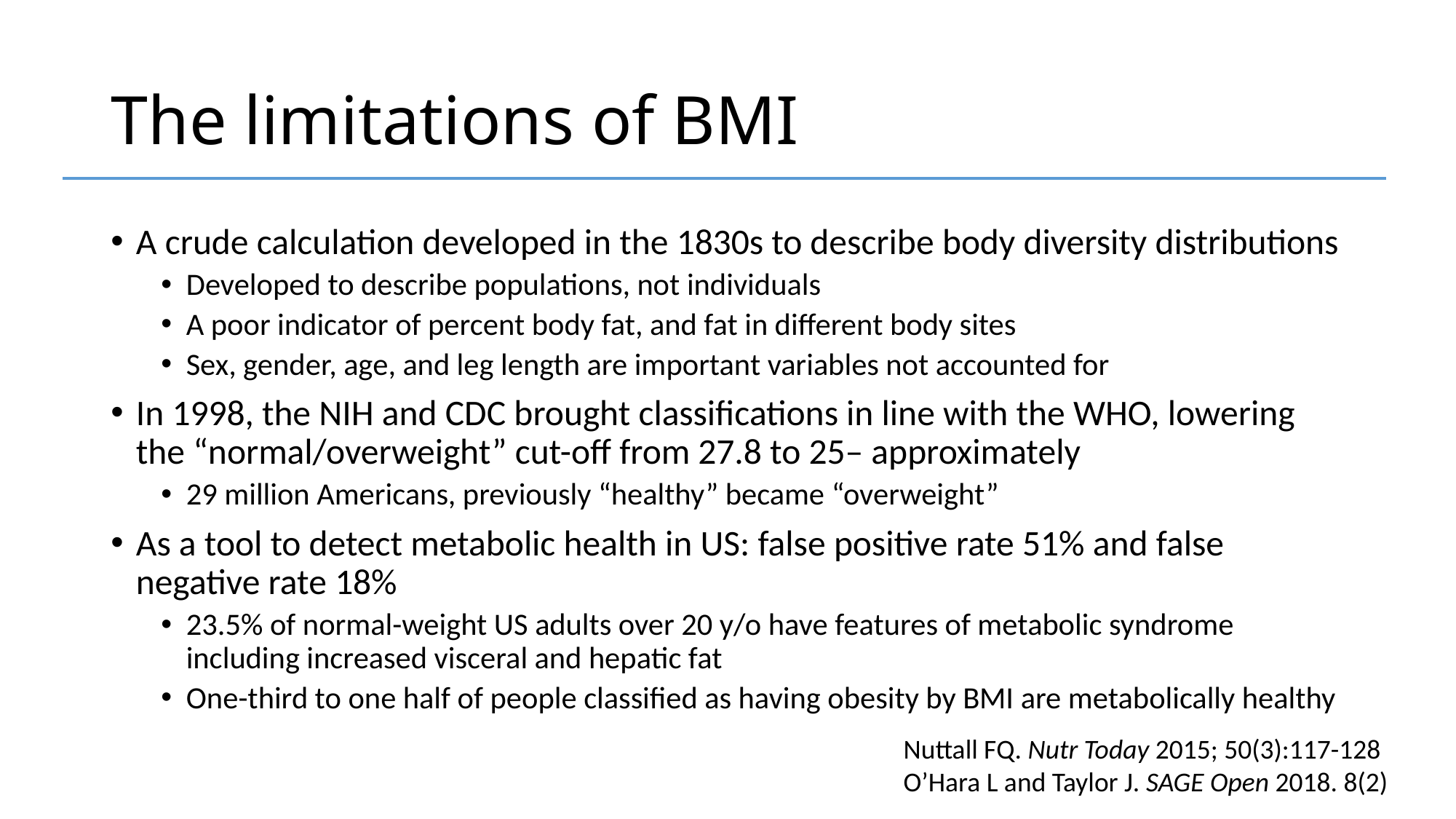

# The limitations of BMI
A crude calculation developed in the 1830s to describe body diversity distributions
Developed to describe populations, not individuals
A poor indicator of percent body fat, and fat in different body sites
Sex, gender, age, and leg length are important variables not accounted for
In 1998, the NIH and CDC brought classifications in line with the WHO, lowering the “normal/overweight” cut-off from 27.8 to 25– approximately
29 million Americans, previously “healthy” became “overweight”
As a tool to detect metabolic health in US: false positive rate 51% and false negative rate 18%
23.5% of normal-weight US adults over 20 y/o have features of metabolic syndrome including increased visceral and hepatic fat
One-third to one half of people classified as having obesity by BMI are metabolically healthy
Nuttall FQ. Nutr Today 2015; 50(3):117-128
O’Hara L and Taylor J. SAGE Open 2018. 8(2)

## Slide 10
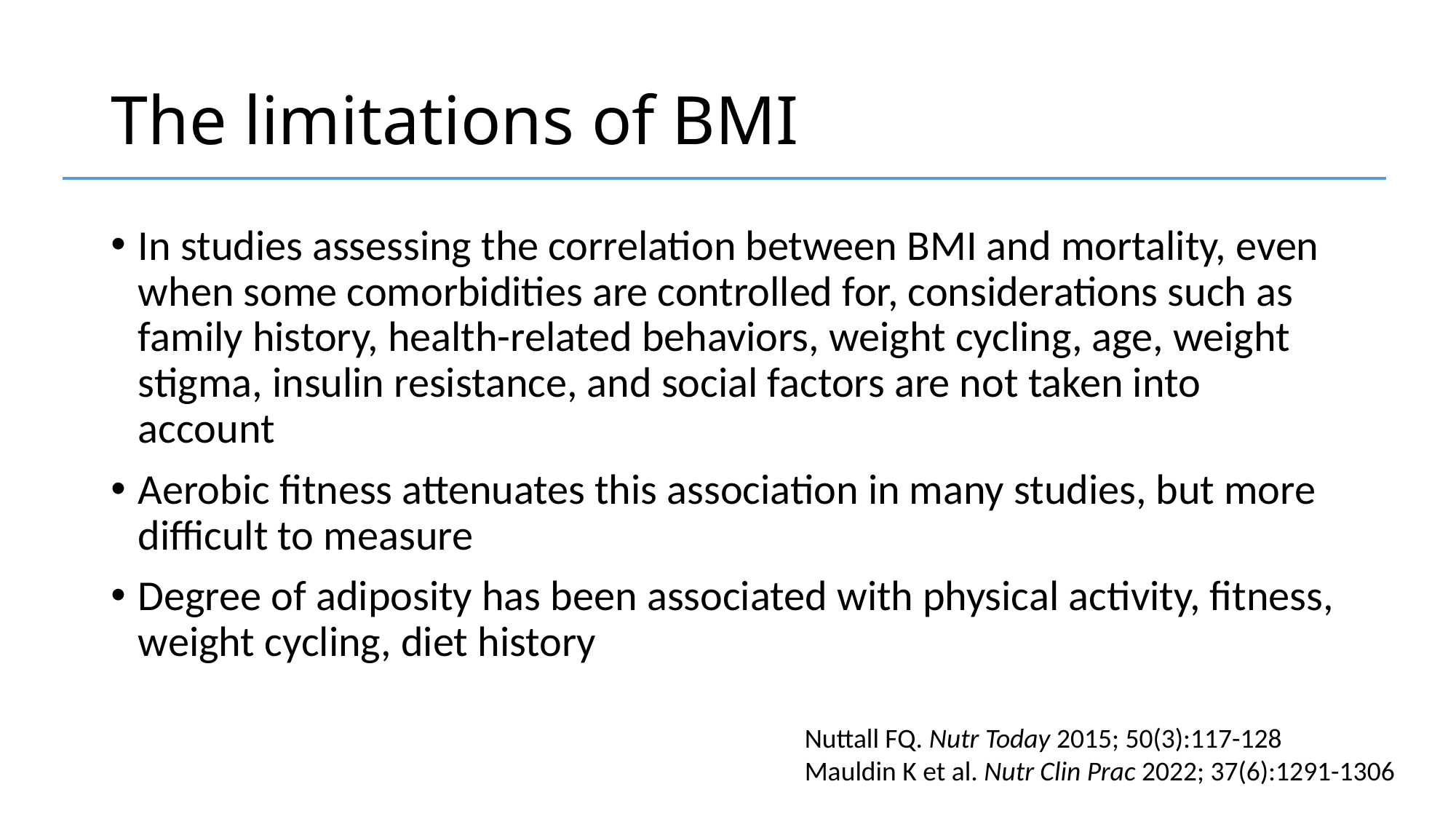

# The limitations of BMI
In studies assessing the correlation between BMI and mortality, even when some comorbidities are controlled for, considerations such as family history, health-related behaviors, weight cycling, age, weight stigma, insulin resistance, and social factors are not taken into account
Aerobic fitness attenuates this association in many studies, but more difficult to measure
Degree of adiposity has been associated with physical activity, fitness, weight cycling, diet history
Nuttall FQ. Nutr Today 2015; 50(3):117-128
Mauldin K et al. Nutr Clin Prac 2022; 37(6):1291-1306

## Slide 11
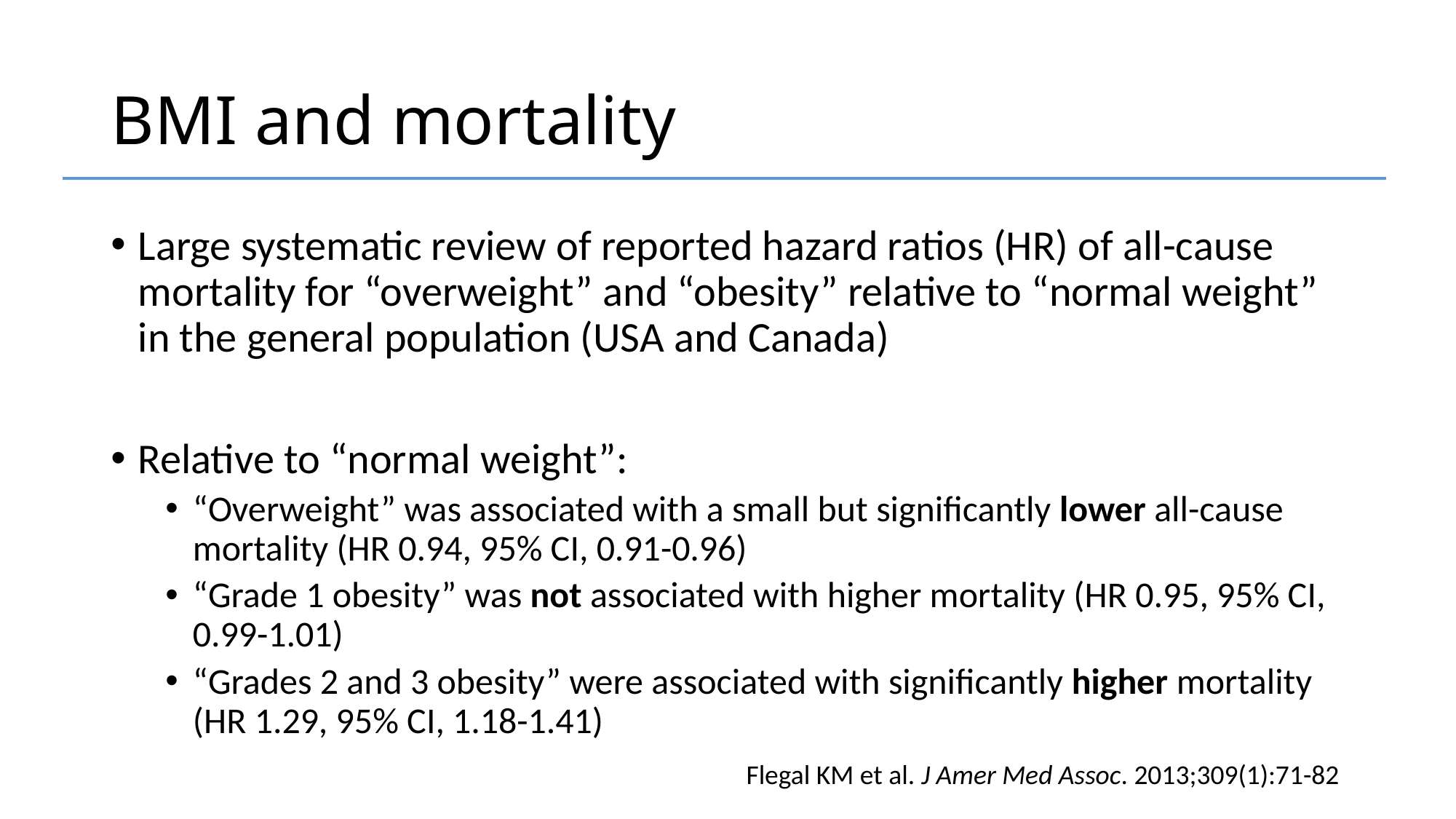

# BMI and mortality
Large systematic review of reported hazard ratios (HR) of all-cause mortality for “overweight” and “obesity” relative to “normal weight” in the general population (USA and Canada)
Relative to “normal weight”:
“Overweight” was associated with a small but significantly lower all-cause mortality (HR 0.94, 95% CI, 0.91-0.96)
“Grade 1 obesity” was not associated with higher mortality (HR 0.95, 95% CI, 0.99-1.01)
“Grades 2 and 3 obesity” were associated with significantly higher mortality (HR 1.29, 95% CI, 1.18-1.41)
Flegal KM et al. J Amer Med Assoc. 2013;309(1):71-82

## Slide 12
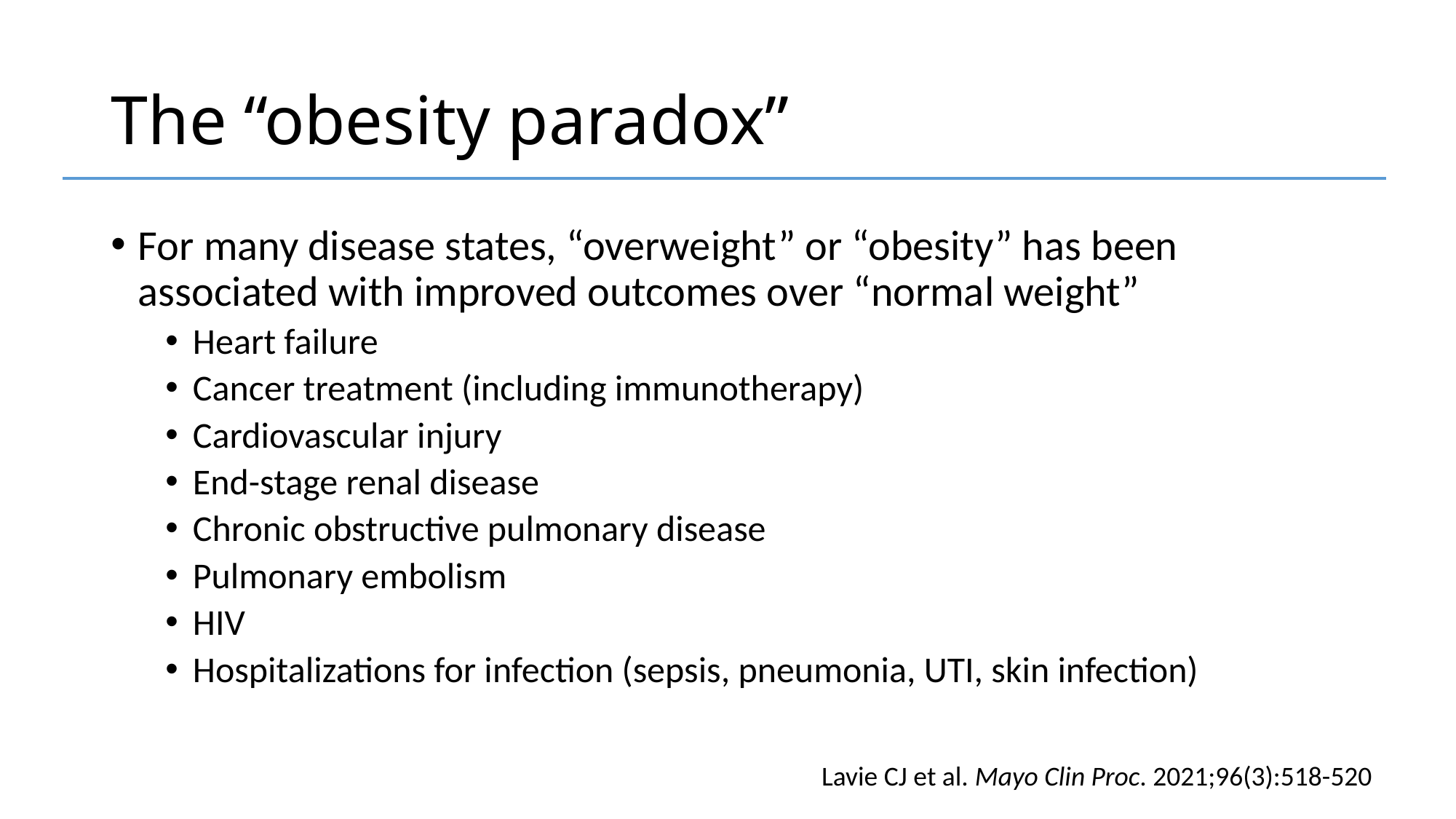

# The “obesity paradox”
For many disease states, “overweight” or “obesity” has been associated with improved outcomes over “normal weight”
Heart failure
Cancer treatment (including immunotherapy)
Cardiovascular injury
End-stage renal disease
Chronic obstructive pulmonary disease
Pulmonary embolism
HIV
Hospitalizations for infection (sepsis, pneumonia, UTI, skin infection)
Lavie CJ et al. Mayo Clin Proc. 2021;96(3):518-520

## Slide 13
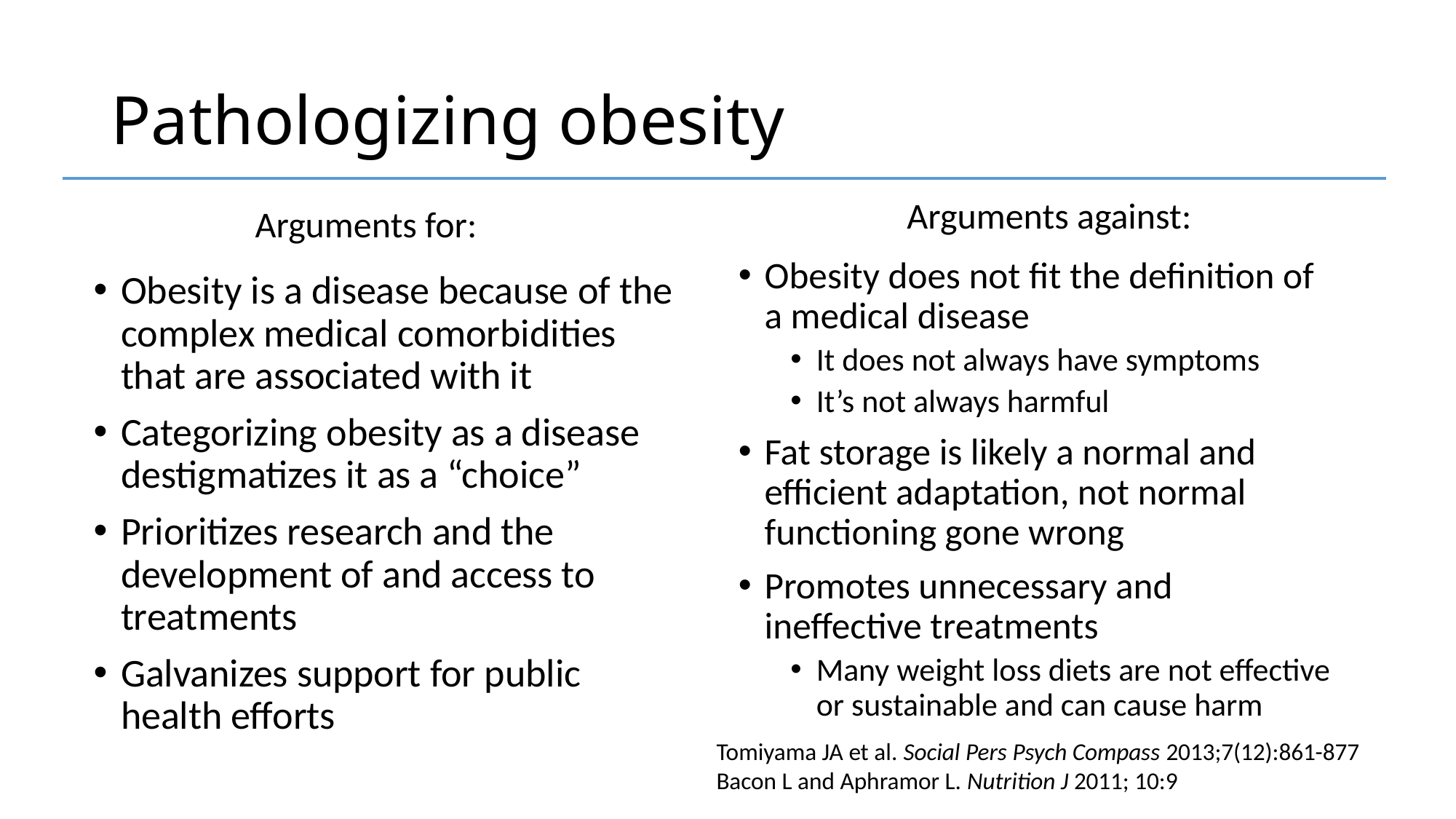

# Pathologizing obesity
Arguments against:
Arguments for:
Obesity does not fit the definition of a medical disease
It does not always have symptoms
It’s not always harmful
Fat storage is likely a normal and efficient adaptation, not normal functioning gone wrong
Promotes unnecessary and ineffective treatments
Many weight loss diets are not effective or sustainable and can cause harm
Obesity is a disease because of the complex medical comorbidities that are associated with it
Categorizing obesity as a disease destigmatizes it as a “choice”
Prioritizes research and the development of and access to treatments
Galvanizes support for public health efforts
Tomiyama JA et al. Social Pers Psych Compass 2013;7(12):861-877
Bacon L and Aphramor L. Nutrition J 2011; 10:9

## Slide 14
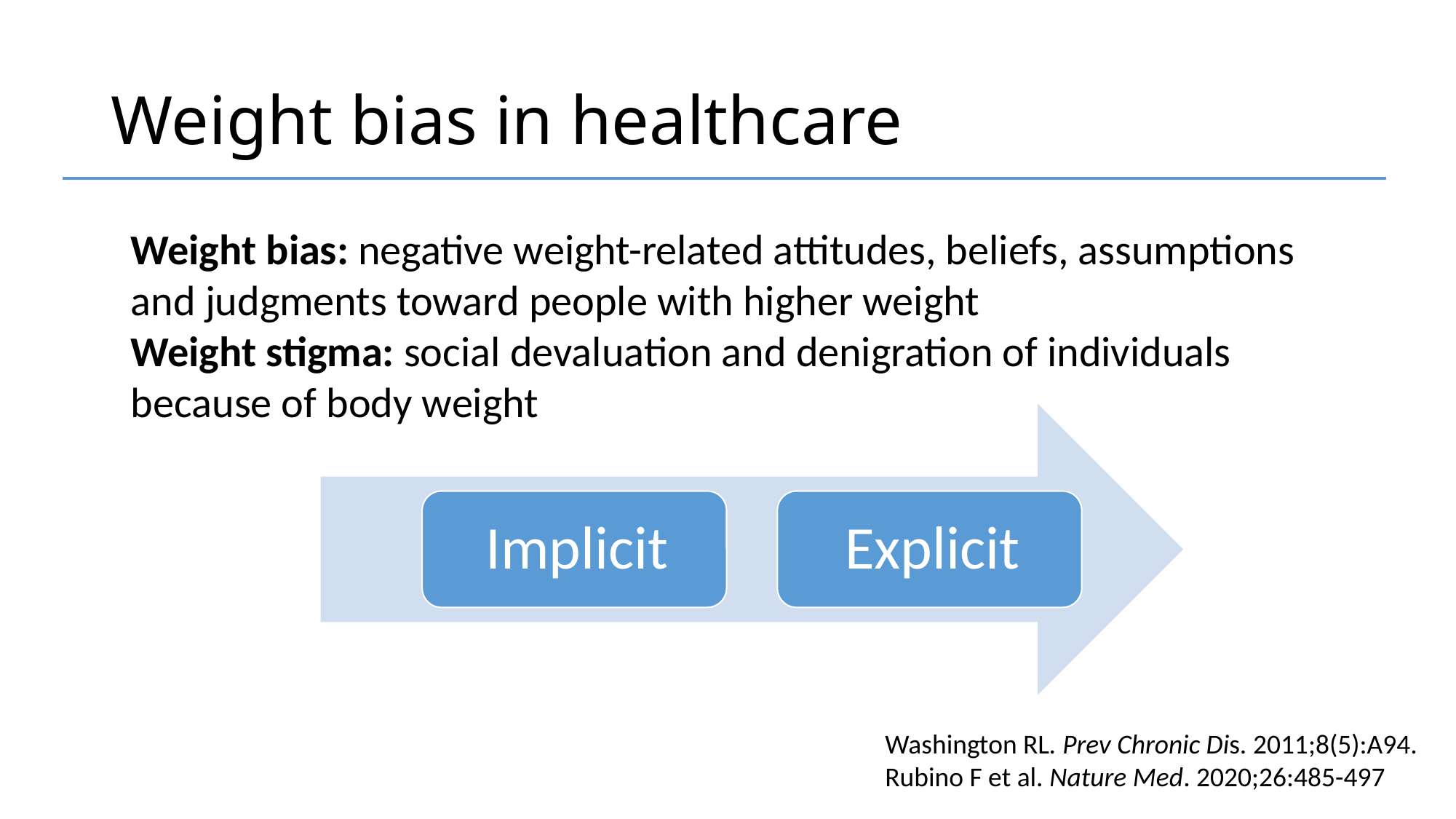

# Weight bias in healthcare
Weight bias: negative weight-related attitudes, beliefs, assumptions and judgments toward people with higher weight
Weight stigma: social devaluation and denigration of individuals because of body weight
Washington RL. Prev Chronic Dis. 2011;8(5):A94.
Rubino F et al. Nature Med. 2020;26:485-497

## Slide 15
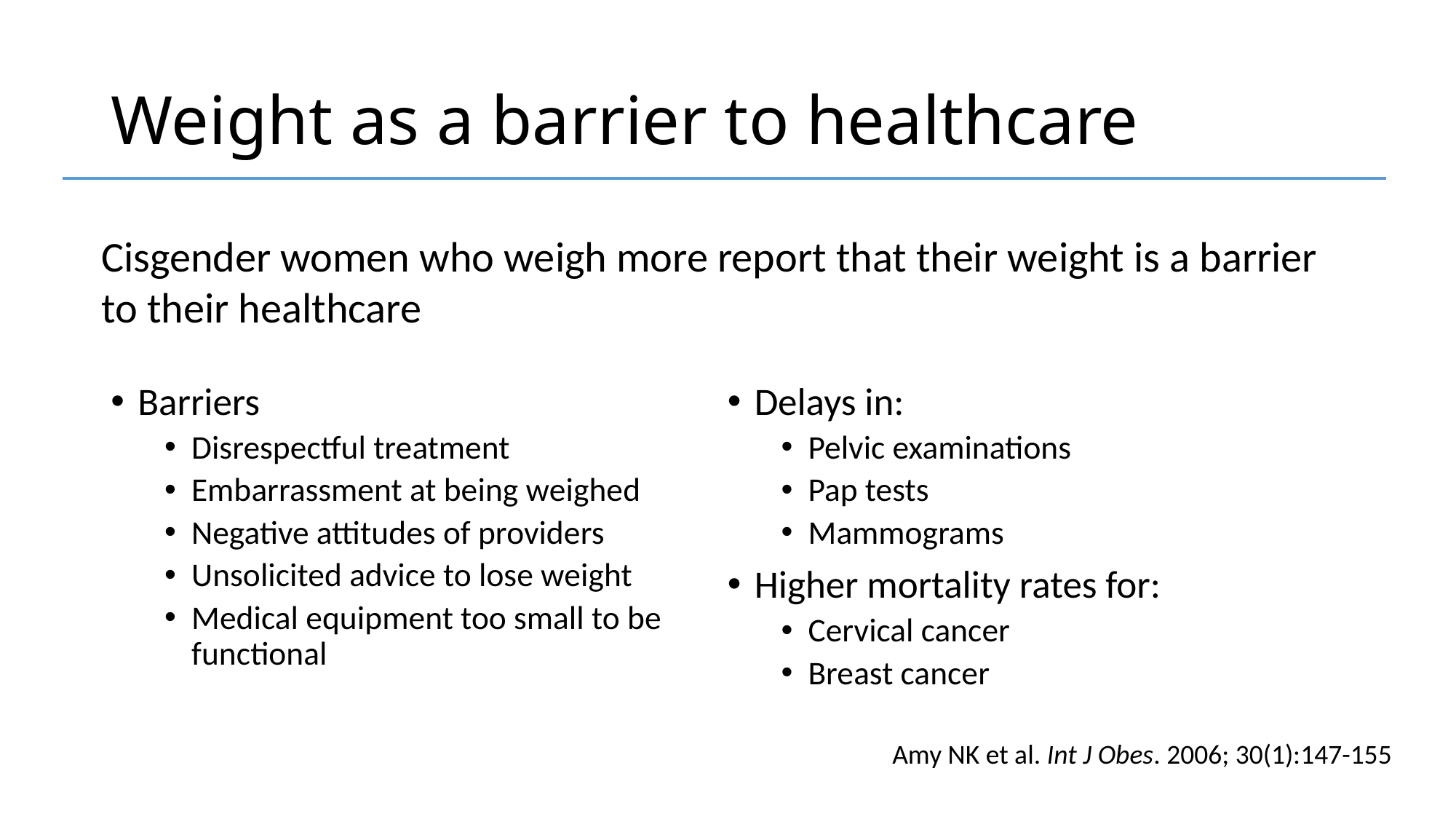

# Weight as a barrier to healthcare
Cisgender women who weigh more report that their weight is a barrier to their healthcare
Barriers
Disrespectful treatment
Embarrassment at being weighed
Negative attitudes of providers
Unsolicited advice to lose weight
Medical equipment too small to be functional
Delays in:
Pelvic examinations
Pap tests
Mammograms
Higher mortality rates for:
Cervical cancer
Breast cancer
Amy NK et al. Int J Obes. 2006; 30(1):147-155

## Slide 16
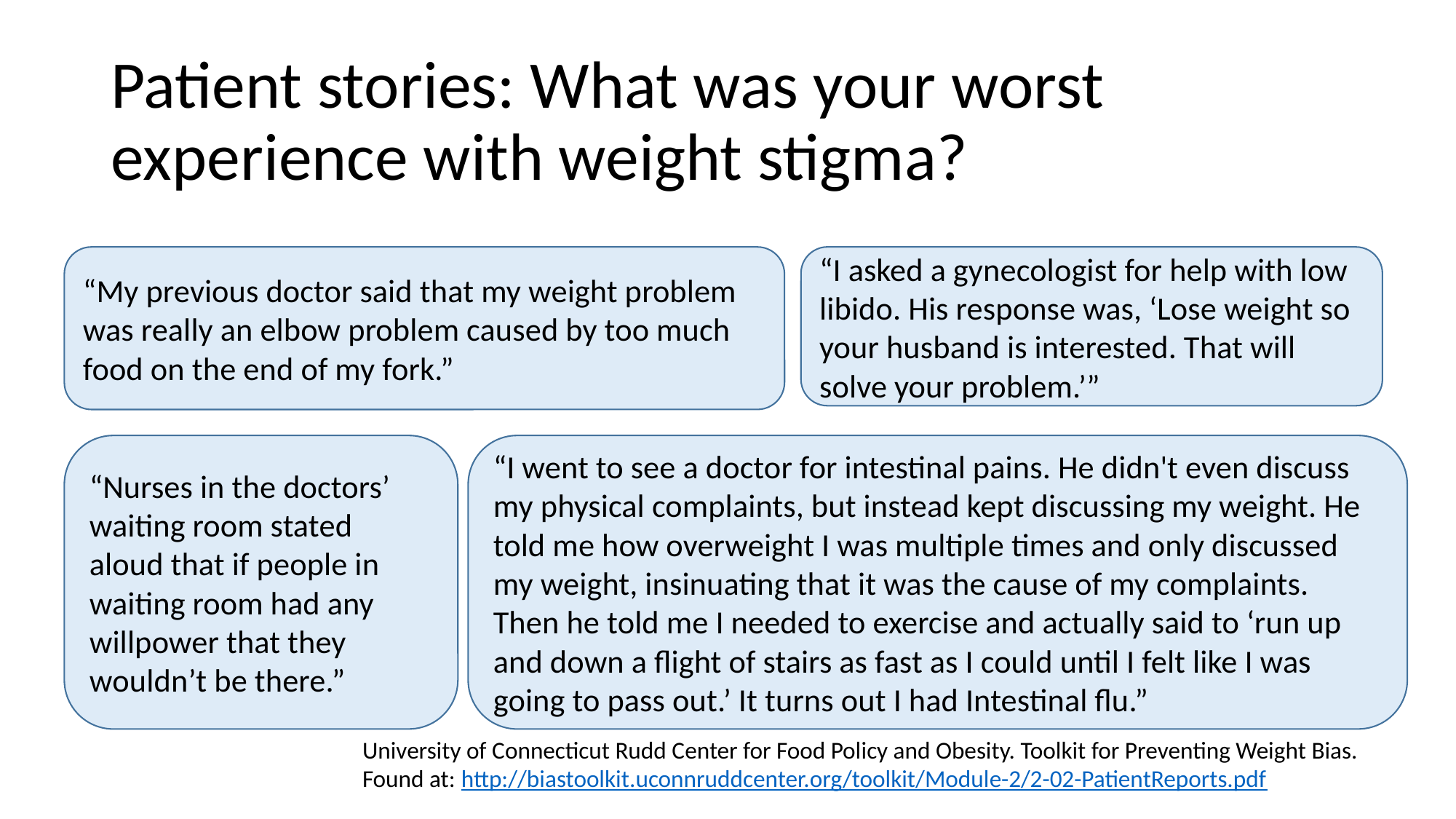

# Patient stories: What was your worst experience with weight stigma?
“My previous doctor said that my weight problem was really an elbow problem caused by too much food on the end of my fork.”
“I asked a gynecologist for help with low libido. His response was, ‘Lose weight so your husband is interested. That will solve your problem.’”
“Nurses in the doctors’ waiting room stated aloud that if people in waiting room had any willpower that they wouldn’t be there.”
“I went to see a doctor for intestinal pains. He didn't even discuss my physical complaints, but instead kept discussing my weight. He told me how overweight I was multiple times and only discussed my weight, insinuating that it was the cause of my complaints. Then he told me I needed to exercise and actually said to ‘run up and down a flight of stairs as fast as I could until I felt like I was going to pass out.’ It turns out I had Intestinal flu.”
University of Connecticut Rudd Center for Food Policy and Obesity. Toolkit for Preventing Weight Bias.
Found at: http://biastoolkit.uconnruddcenter.org/toolkit/Module-2/2-02-PatientReports.pdf

## Slide 17
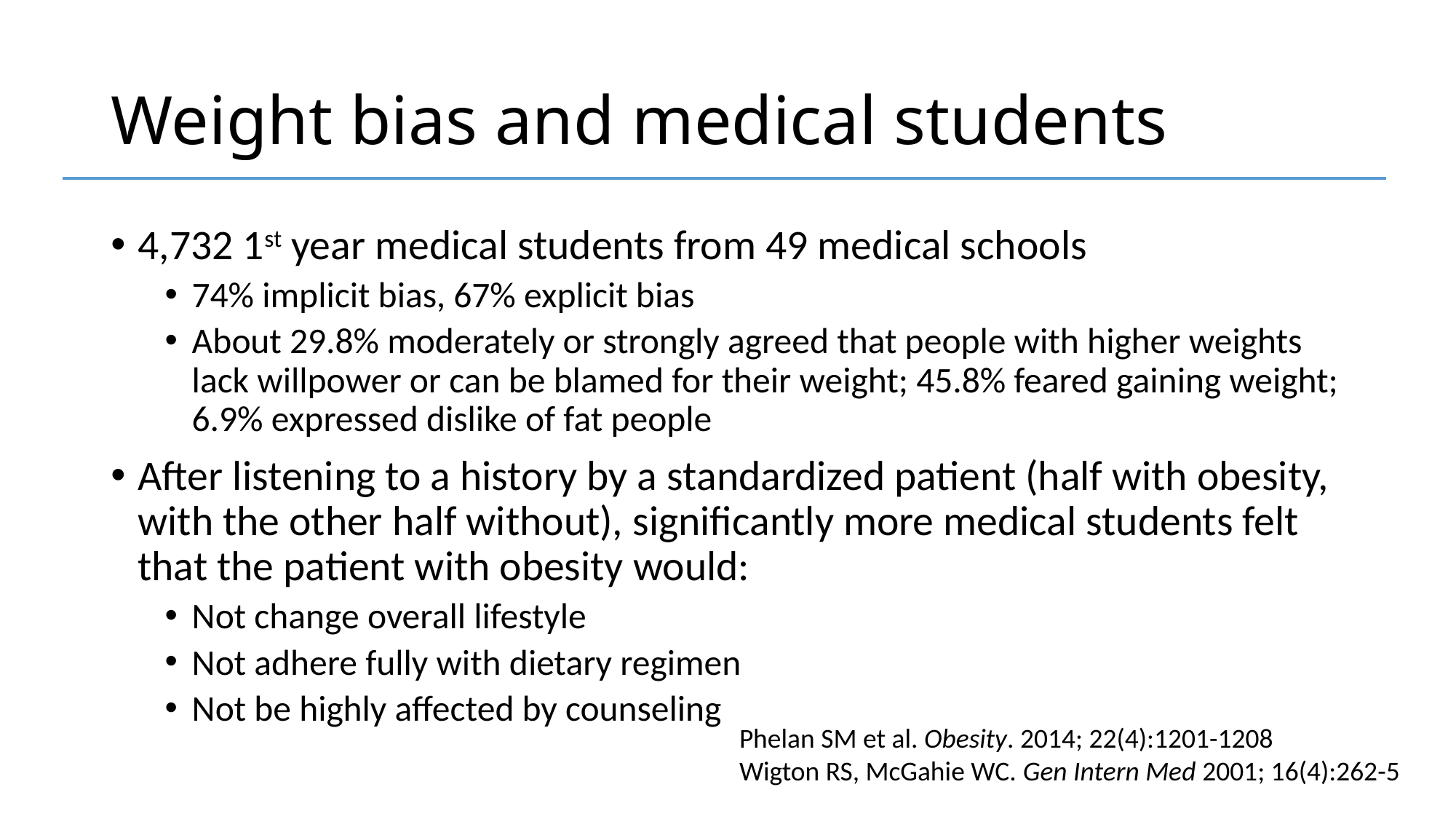

# Weight bias and medical students
4,732 1st year medical students from 49 medical schools
74% implicit bias, 67% explicit bias
About 29.8% moderately or strongly agreed that people with higher weights lack willpower or can be blamed for their weight; 45.8% feared gaining weight; 6.9% expressed dislike of fat people
After listening to a history by a standardized patient (half with obesity, with the other half without), significantly more medical students felt that the patient with obesity would:
Not change overall lifestyle
Not adhere fully with dietary regimen
Not be highly affected by counseling
Phelan SM et al. Obesity. 2014; 22(4):1201-1208
Wigton RS, McGahie WC. Gen Intern Med 2001; 16(4):262-5

## Slide 18
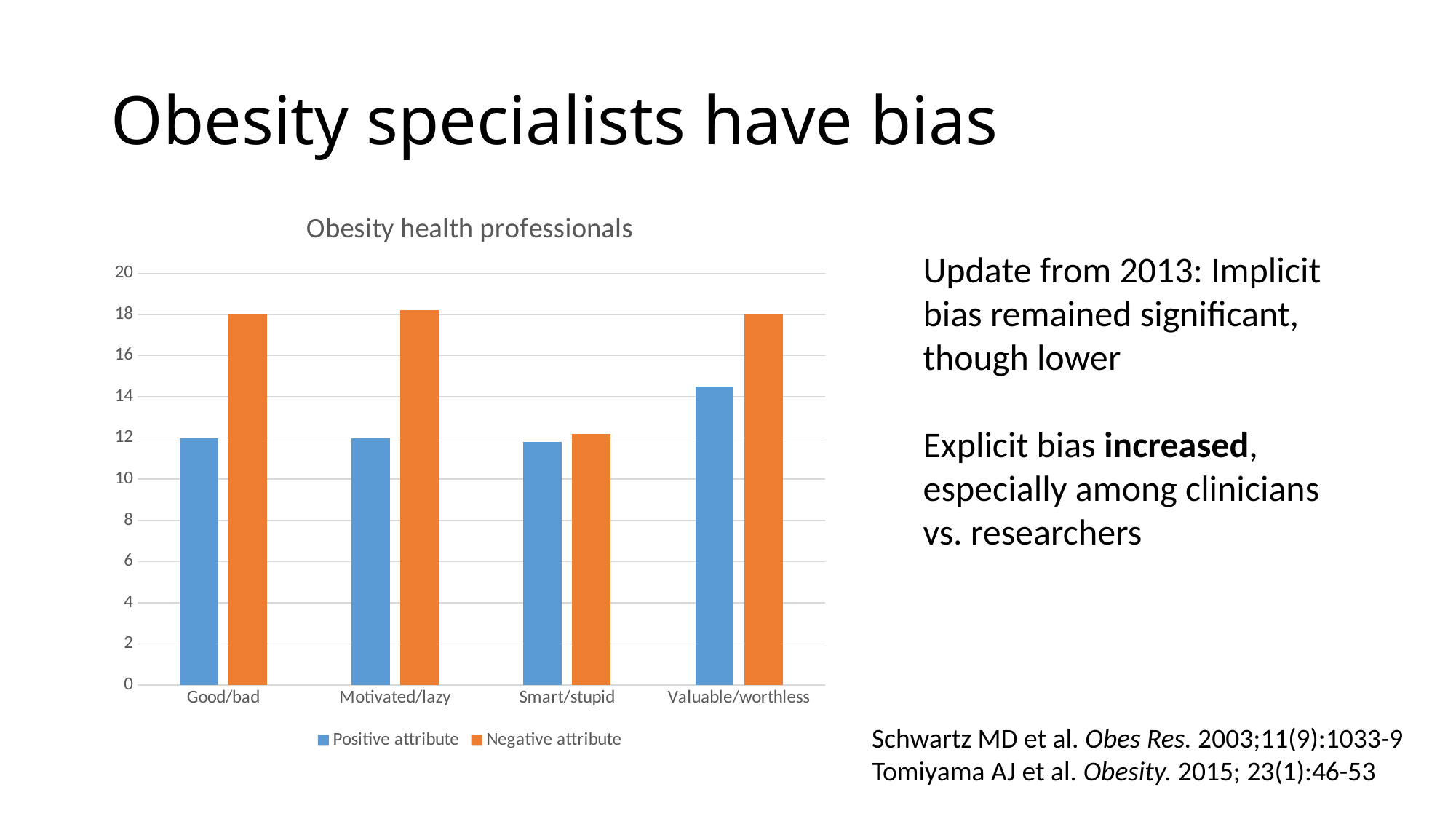

# Obesity specialists have bias
### Chart: Obesity health professionals
| Category | Positive attribute | Negative attribute |
|---|---|---|
| Good/bad | 12.0 | 18.0 |
| Motivated/lazy | 12.0 | 18.2 |
| Smart/stupid | 11.8 | 12.2 |
| Valuable/worthless | 14.5 | 18.0 |Update from 2013: Implicit bias remained significant, though lower
Explicit bias increased, especially among clinicians vs. researchers
Schwartz MD et al. Obes Res. 2003;11(9):1033-9
Tomiyama AJ et al. Obesity. 2015; 23(1):46-53

## Slide 19
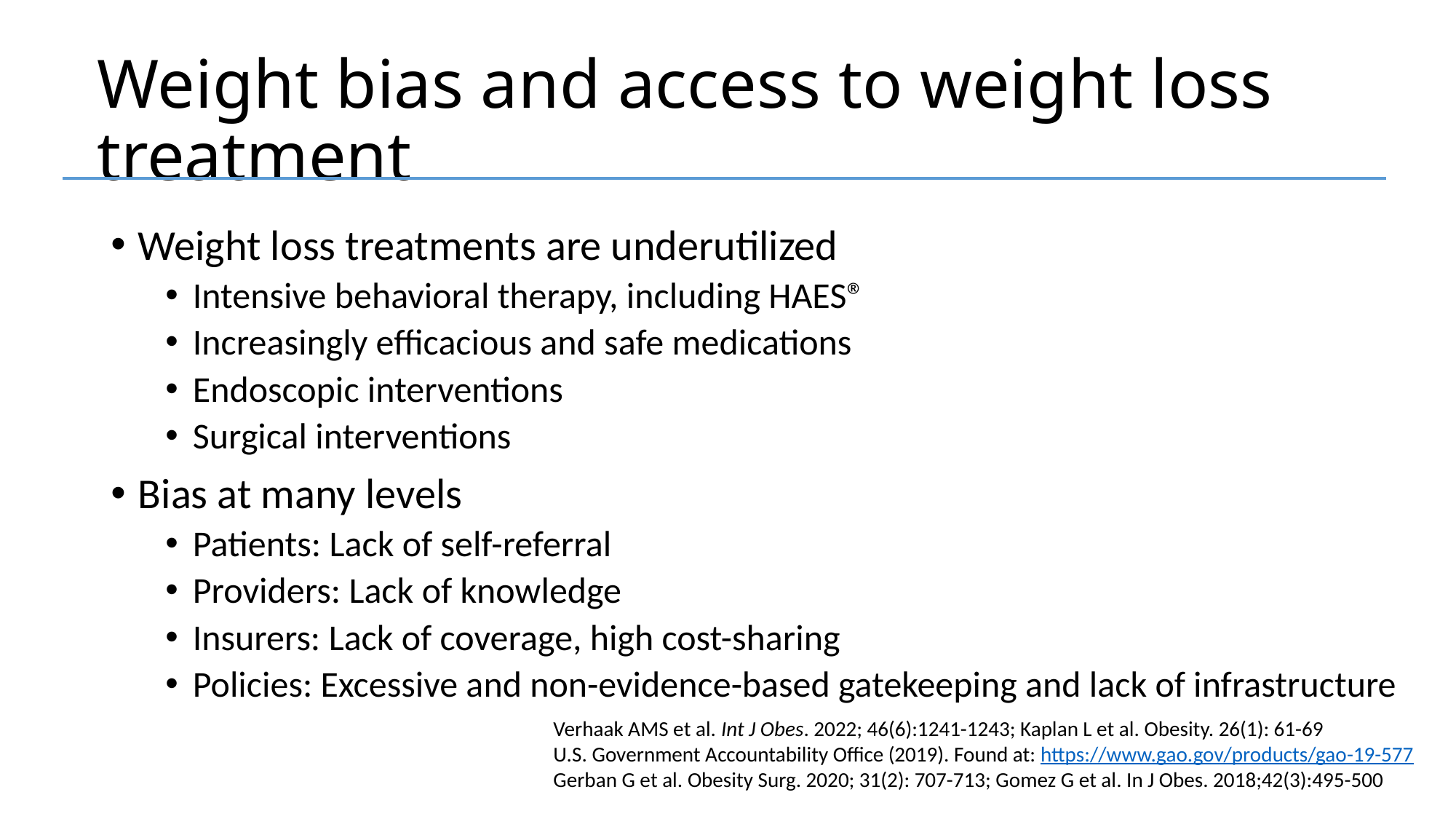

# Weight bias and access to weight loss treatment
Weight loss treatments are underutilized
Intensive behavioral therapy, including HAES®
Increasingly efficacious and safe medications
Endoscopic interventions
Surgical interventions
Bias at many levels
Patients: Lack of self-referral
Providers: Lack of knowledge
Insurers: Lack of coverage, high cost-sharing
Policies: Excessive and non-evidence-based gatekeeping and lack of infrastructure
Verhaak AMS et al. Int J Obes. 2022; 46(6):1241-1243; Kaplan L et al. Obesity. 26(1): 61-69
U.S. Government Accountability Office (2019). Found at: https://www.gao.gov/products/gao-19-577
Gerban G et al. Obesity Surg. 2020; 31(2): 707-713; Gomez G et al. In J Obes. 2018;42(3):495-500

## Slide 20
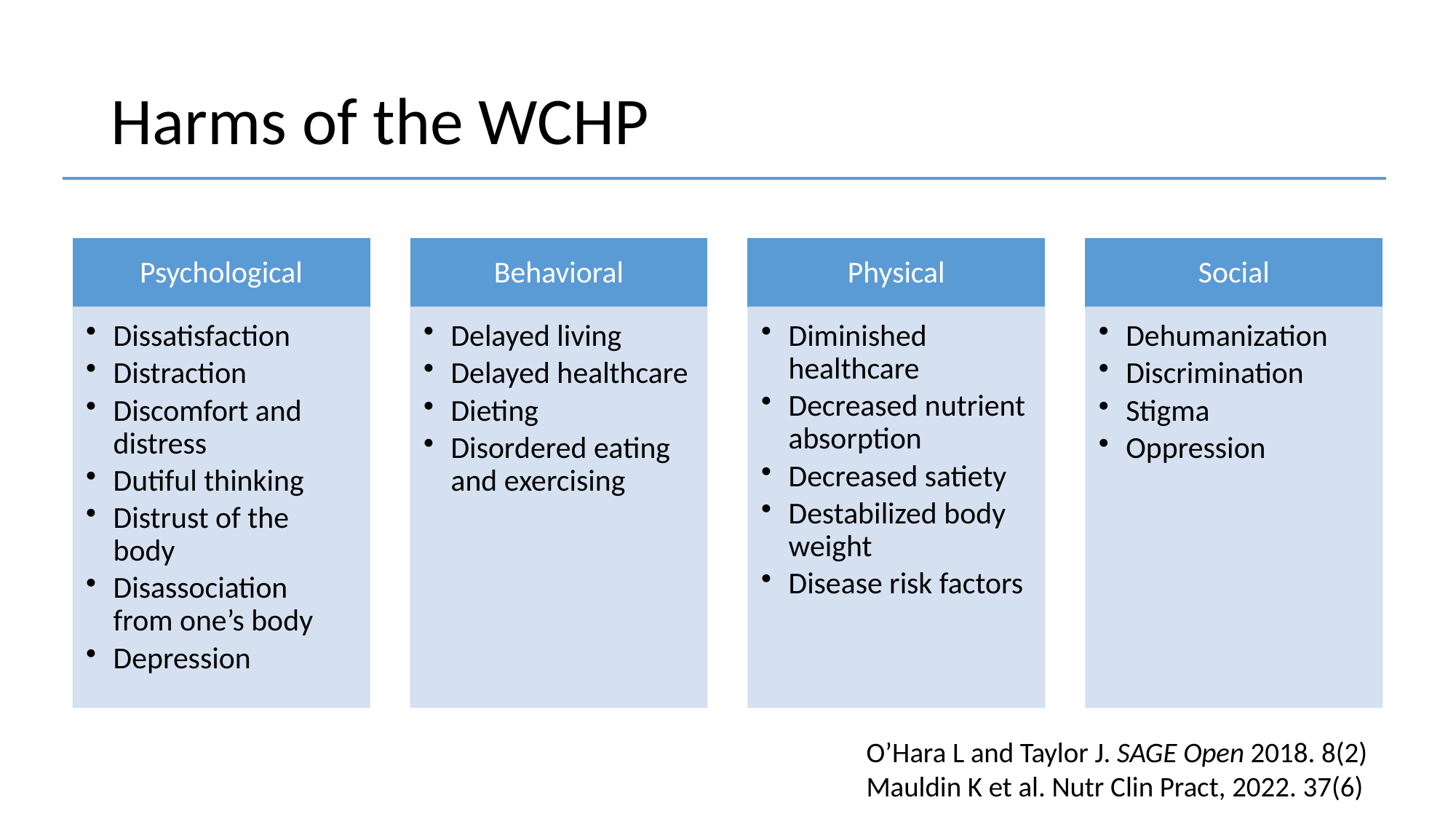

# Harms of the WCHP
O’Hara L and Taylor J. SAGE Open 2018. 8(2)
Mauldin K et al. Nutr Clin Pract, 2022. 37(6)

## Slide 21
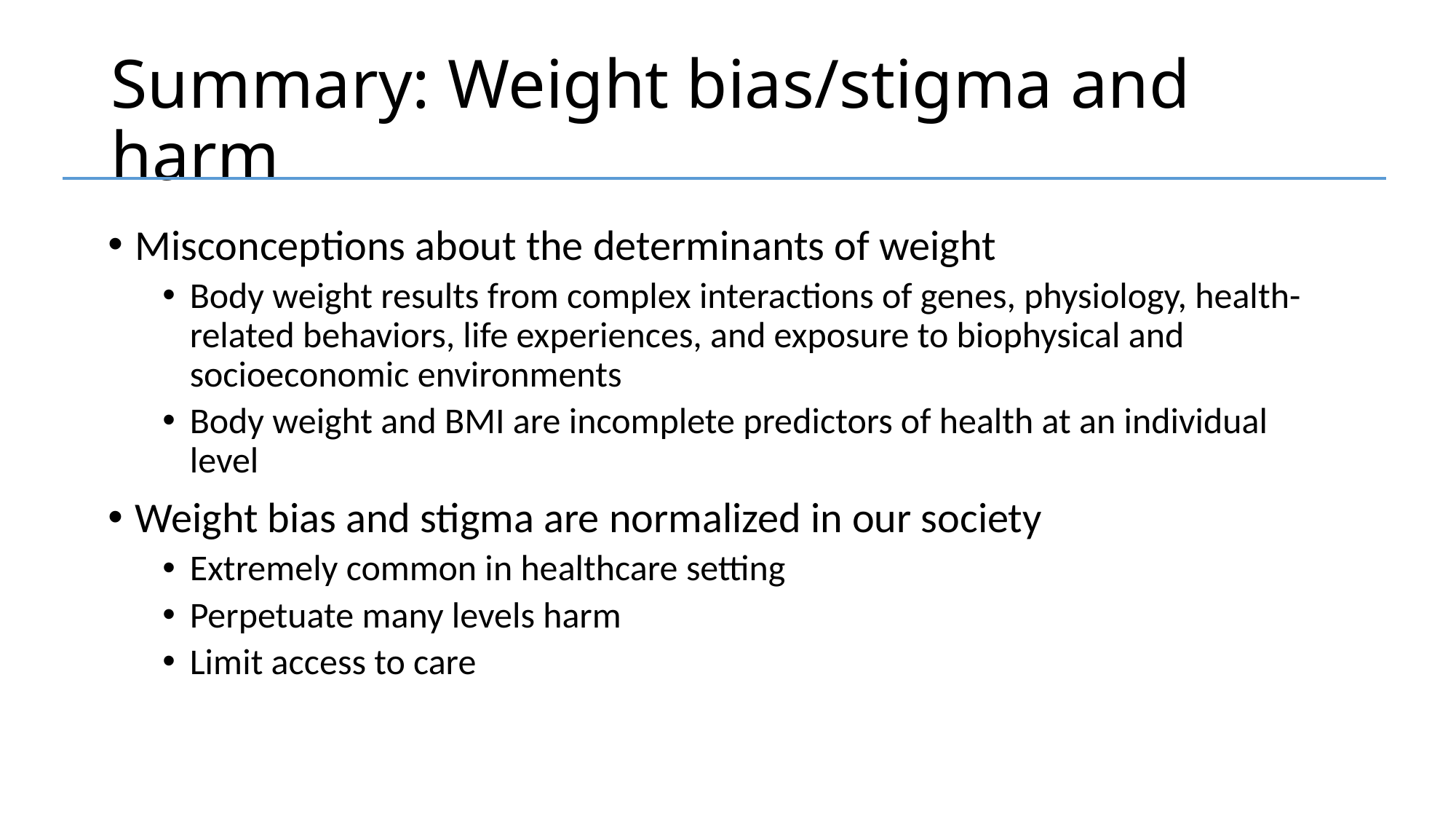

# Summary: Weight bias/stigma and harm
Misconceptions about the determinants of weight
Body weight results from complex interactions of genes, physiology, health-related behaviors, life experiences, and exposure to biophysical and socioeconomic environments
Body weight and BMI are incomplete predictors of health at an individual level
Weight bias and stigma are normalized in our society
Extremely common in healthcare setting
Perpetuate many levels harm
Limit access to care

## Slide 22
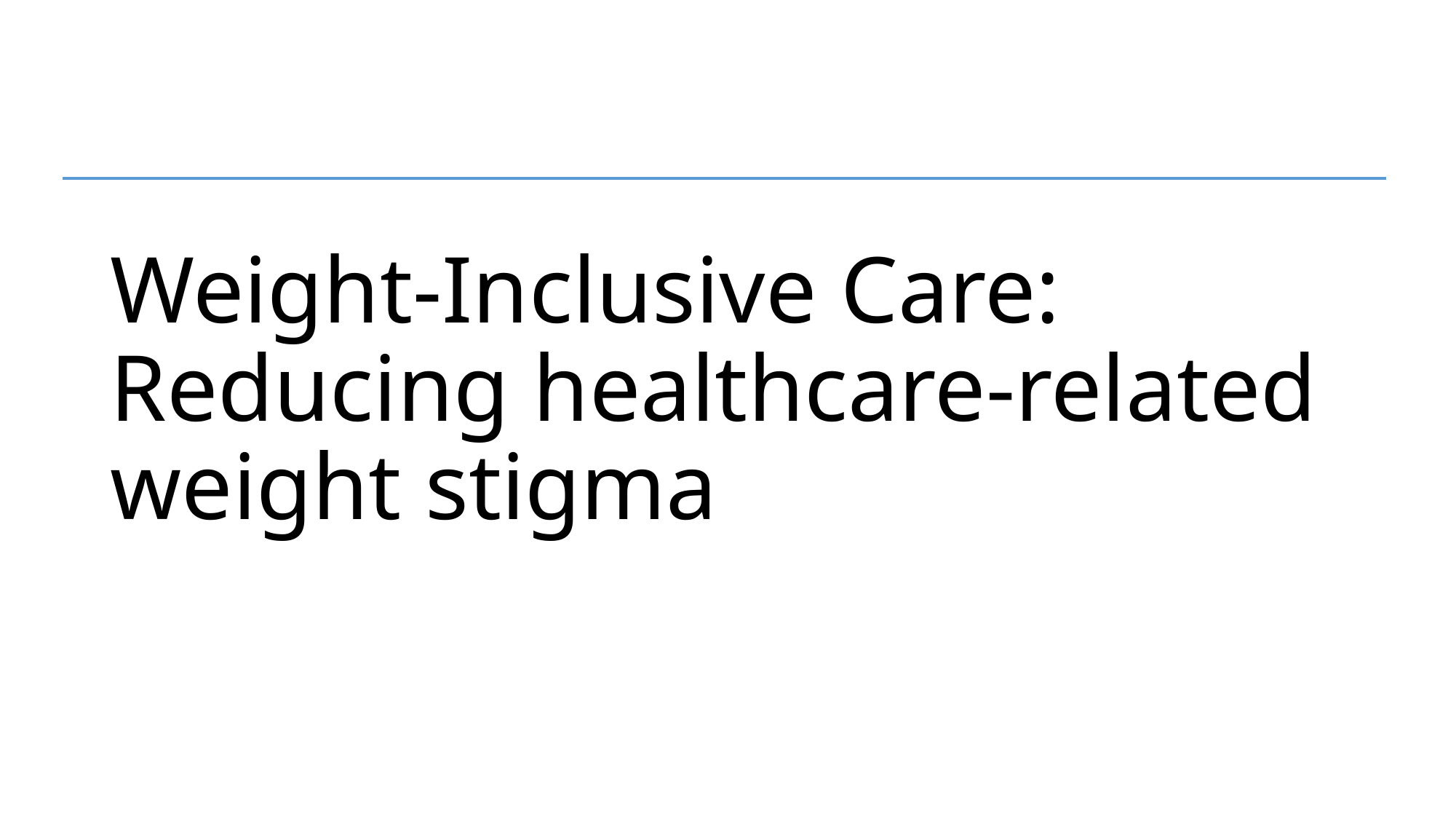

# Weight-Inclusive Care: Reducing healthcare-related weight stigma

## Slide 23
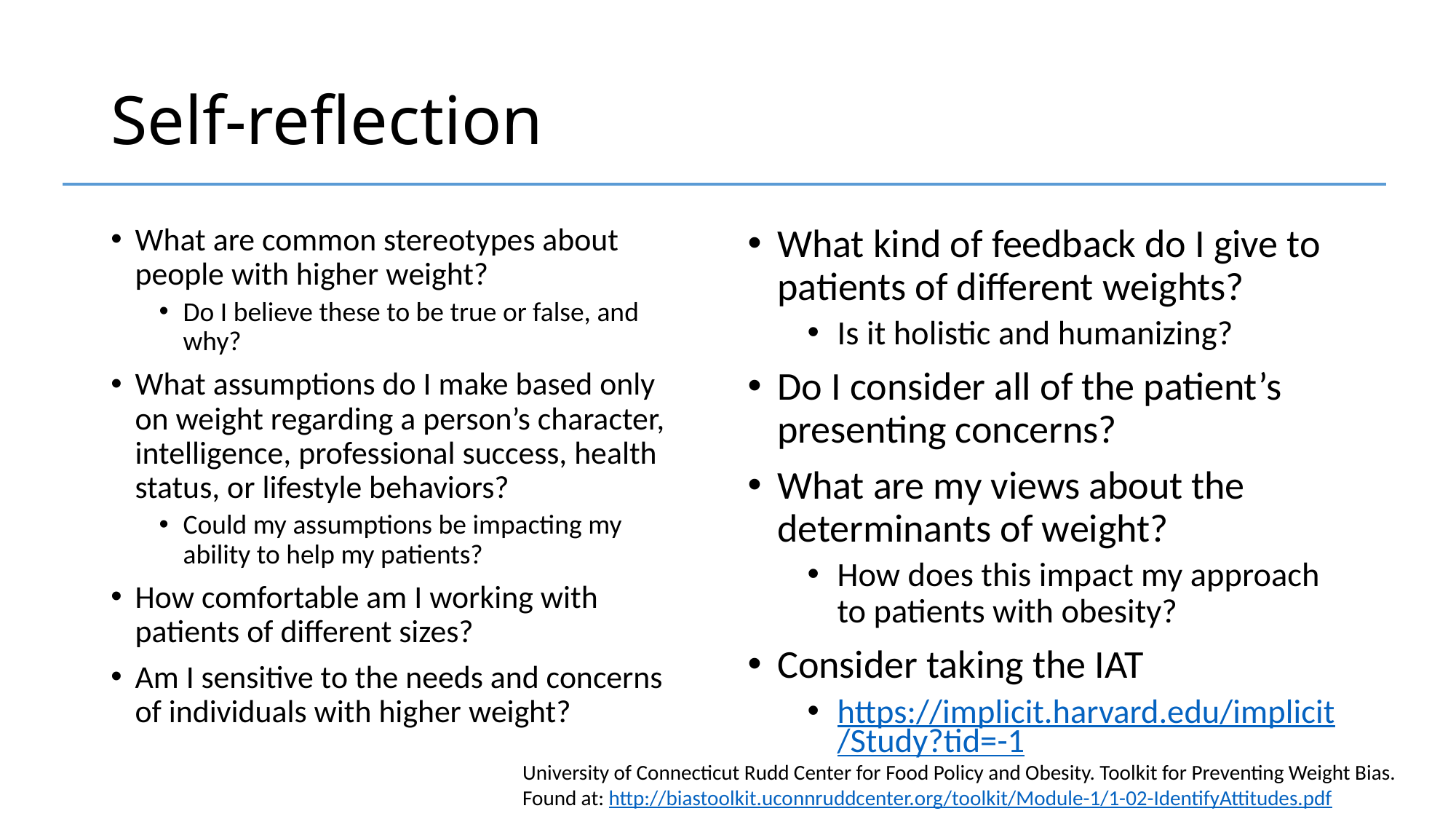

# Self-reflection
What are common stereotypes about people with higher weight?
Do I believe these to be true or false, and why?
What assumptions do I make based only on weight regarding a person’s character, intelligence, professional success, health status, or lifestyle behaviors?
Could my assumptions be impacting my ability to help my patients?
How comfortable am I working with patients of different sizes?
Am I sensitive to the needs and concerns of individuals with higher weight?
What kind of feedback do I give to patients of different weights?
Is it holistic and humanizing?
Do I consider all of the patient’s presenting concerns?
What are my views about the determinants of weight?
How does this impact my approach to patients with obesity?
Consider taking the IAT
https://implicit.harvard.edu/implicit/Study?tid=-1
University of Connecticut Rudd Center for Food Policy and Obesity. Toolkit for Preventing Weight Bias.
Found at: http://biastoolkit.uconnruddcenter.org/toolkit/Module-1/1-02-IdentifyAttitudes.pdf

## Slide 24
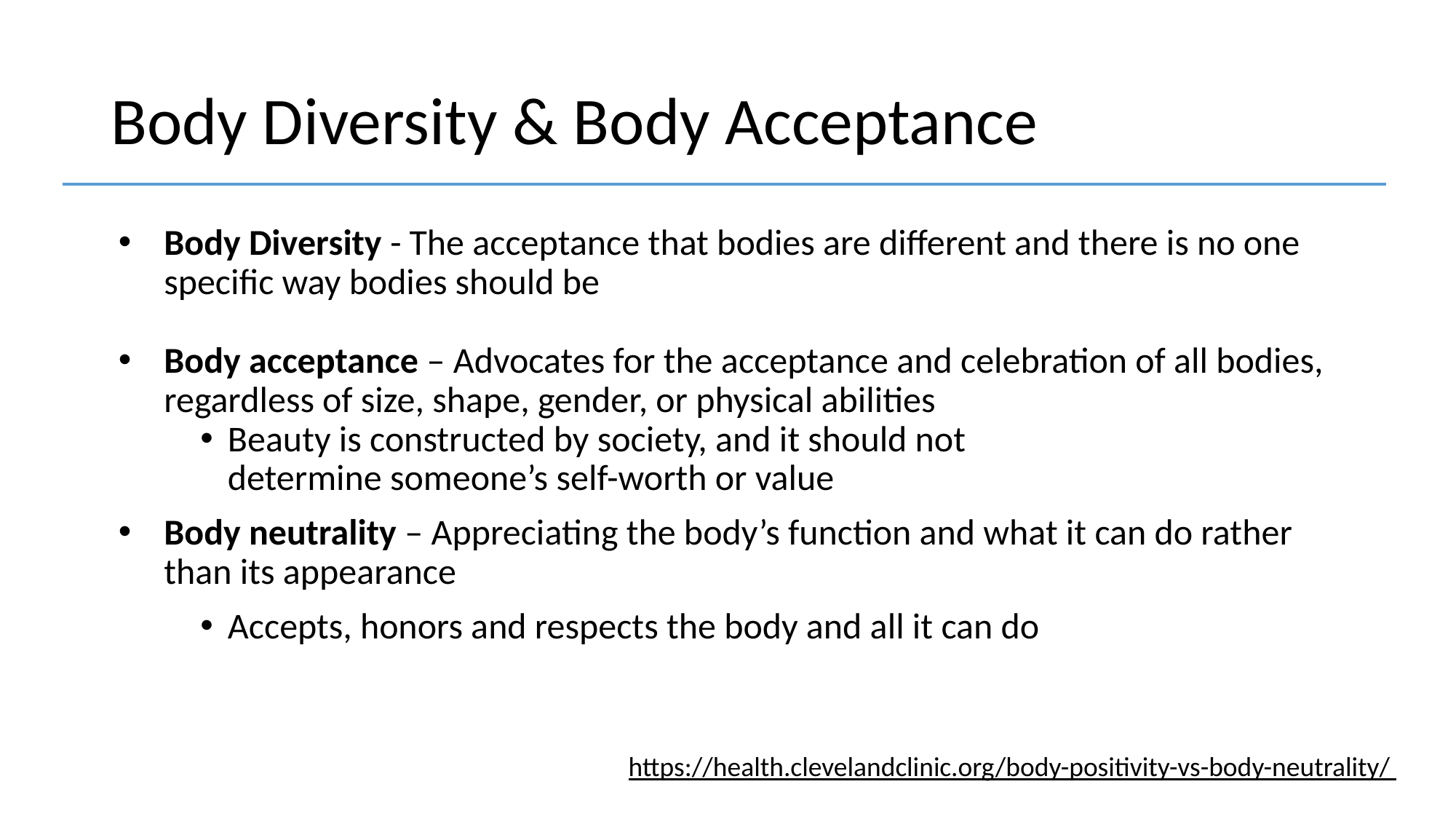

# Body Diversity & Body Acceptance
Body Diversity - The acceptance that bodies are different and there is no one specific way bodies should be
Body acceptance – Advocates for the acceptance and celebration of all bodies, regardless of size, shape, gender, or physical abilities
Beauty is constructed by society, and it should not
determine someone’s self-worth or value
Body neutrality – Appreciating the body’s function and what it can do rather than its appearance
Accepts, honors and respects the body and all it can do
https://health.clevelandclinic.org/body-positivity-vs-body-neutrality/

## Slide 25
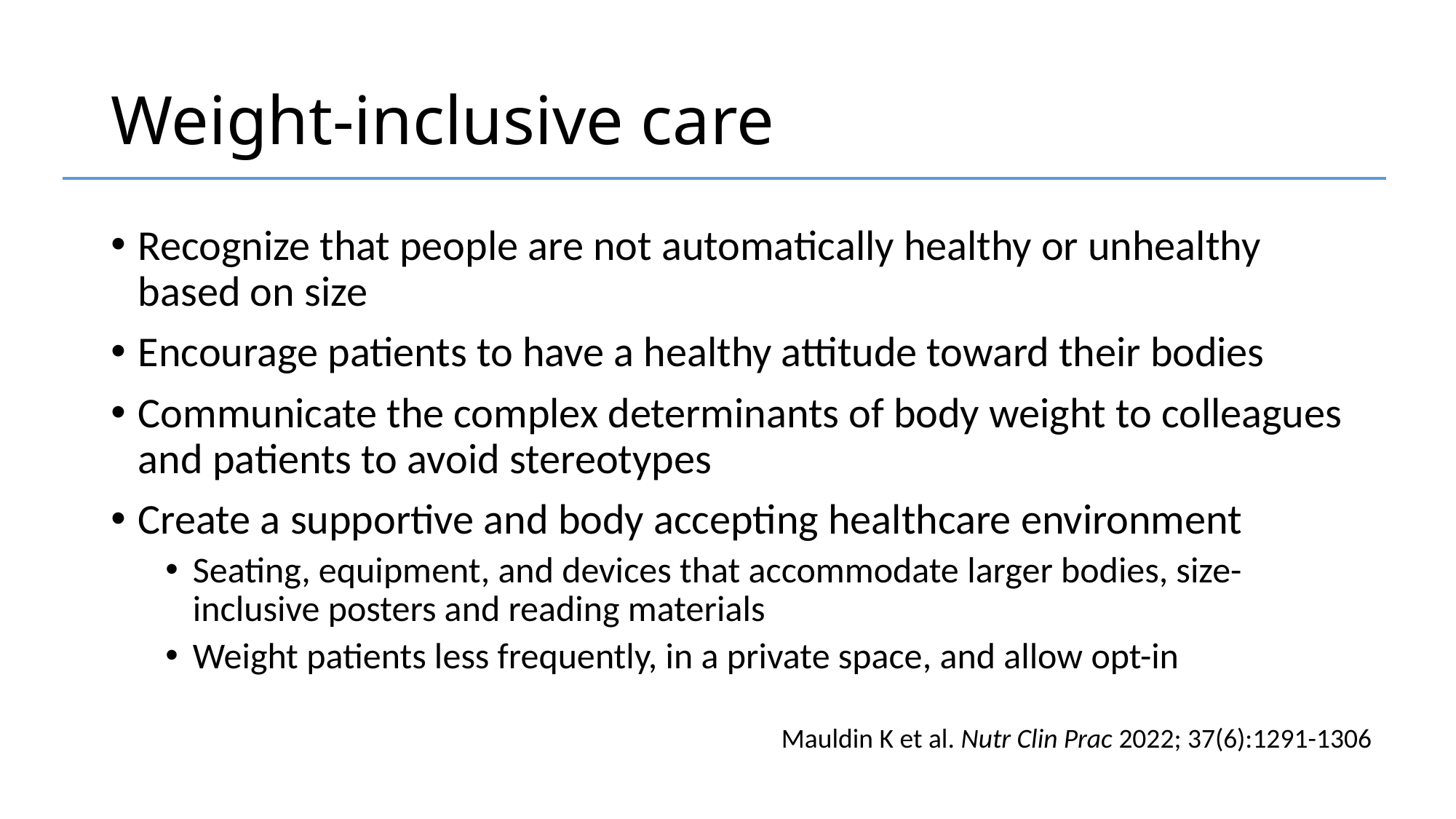

# Weight-inclusive care
Recognize that people are not automatically healthy or unhealthy based on size
Encourage patients to have a healthy attitude toward their bodies
Communicate the complex determinants of body weight to colleagues and patients to avoid stereotypes
Create a supportive and body accepting healthcare environment
Seating, equipment, and devices that accommodate larger bodies, size-inclusive posters and reading materials
Weight patients less frequently, in a private space, and allow opt-in
Mauldin K et al. Nutr Clin Prac 2022; 37(6):1291-1306

## Slide 26
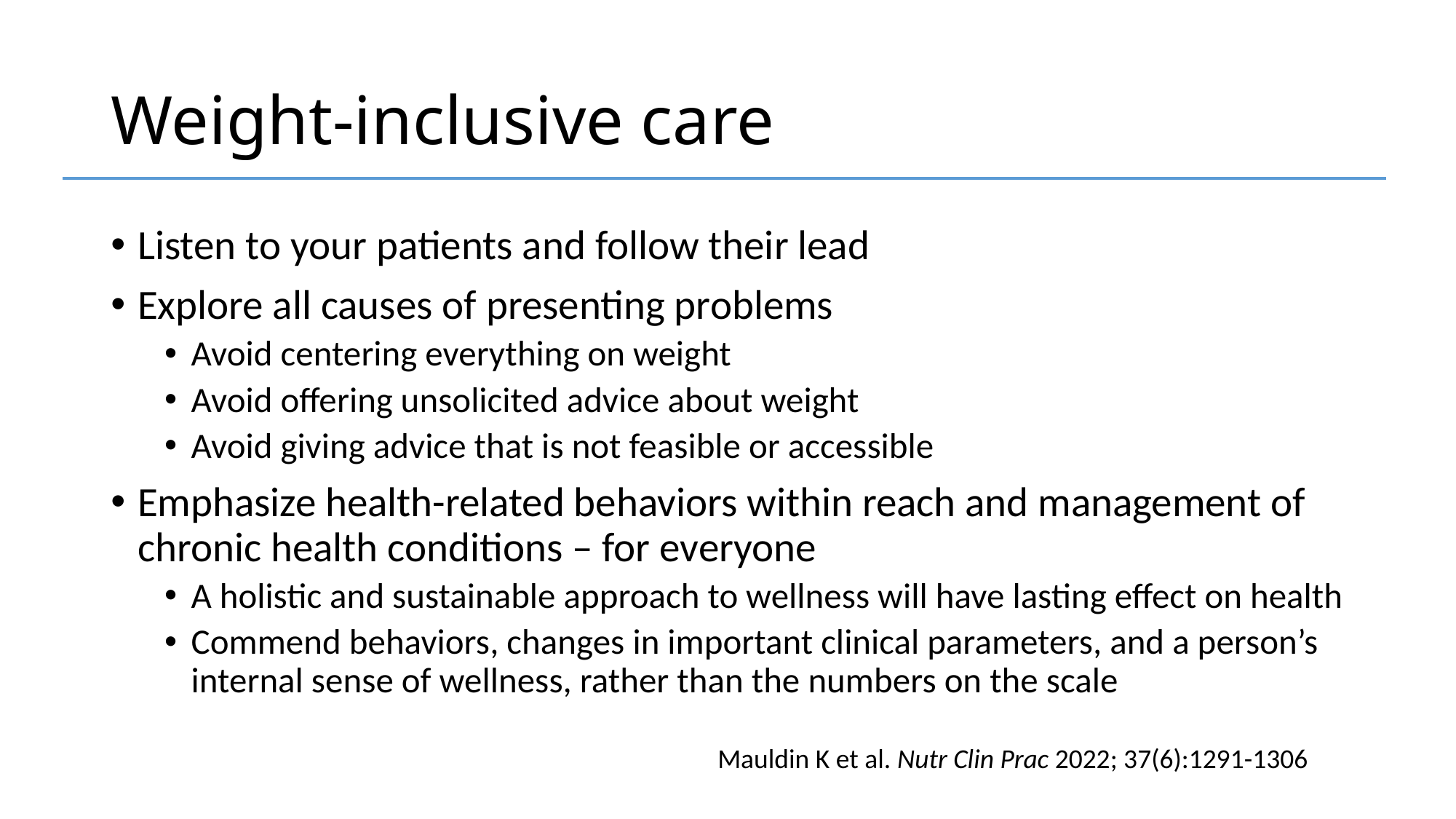

# Weight-inclusive care
Listen to your patients and follow their lead
Explore all causes of presenting problems
Avoid centering everything on weight
Avoid offering unsolicited advice about weight
Avoid giving advice that is not feasible or accessible
Emphasize health-related behaviors within reach and management of chronic health conditions – for everyone
A holistic and sustainable approach to wellness will have lasting effect on health
Commend behaviors, changes in important clinical parameters, and a person’s internal sense of wellness, rather than the numbers on the scale
Mauldin K et al. Nutr Clin Prac 2022; 37(6):1291-1306

## Slide 27
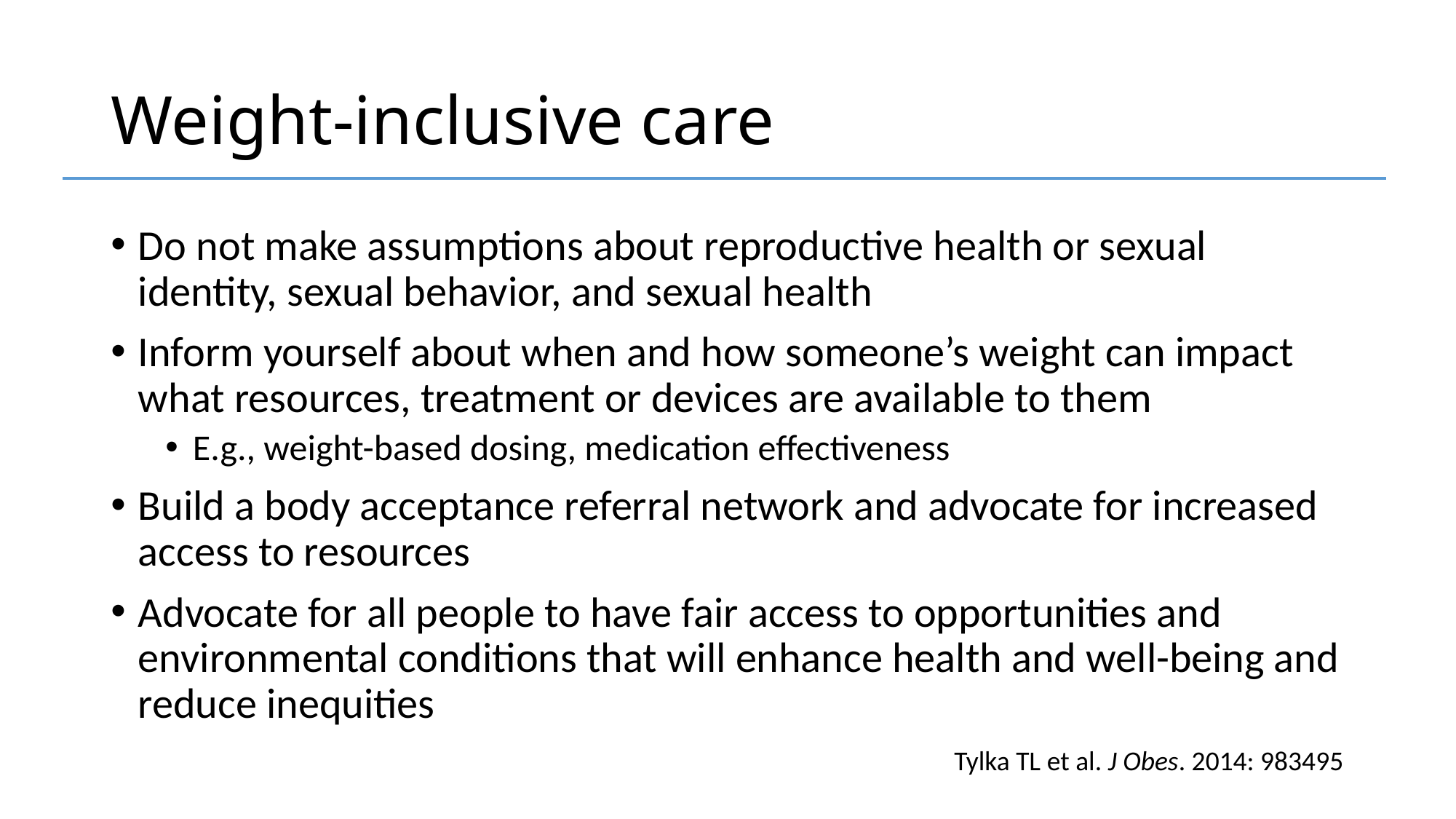

# Weight-inclusive care
Do not make assumptions about reproductive health or sexual identity, sexual behavior, and sexual health
Inform yourself about when and how someone’s weight can impact what resources, treatment or devices are available to them
E.g., weight-based dosing, medication effectiveness
Build a body acceptance referral network and advocate for increased access to resources
Advocate for all people to have fair access to opportunities and environmental conditions that will enhance health and well-being and reduce inequities
Tylka TL et al. J Obes. 2014: 983495

## Slide 28
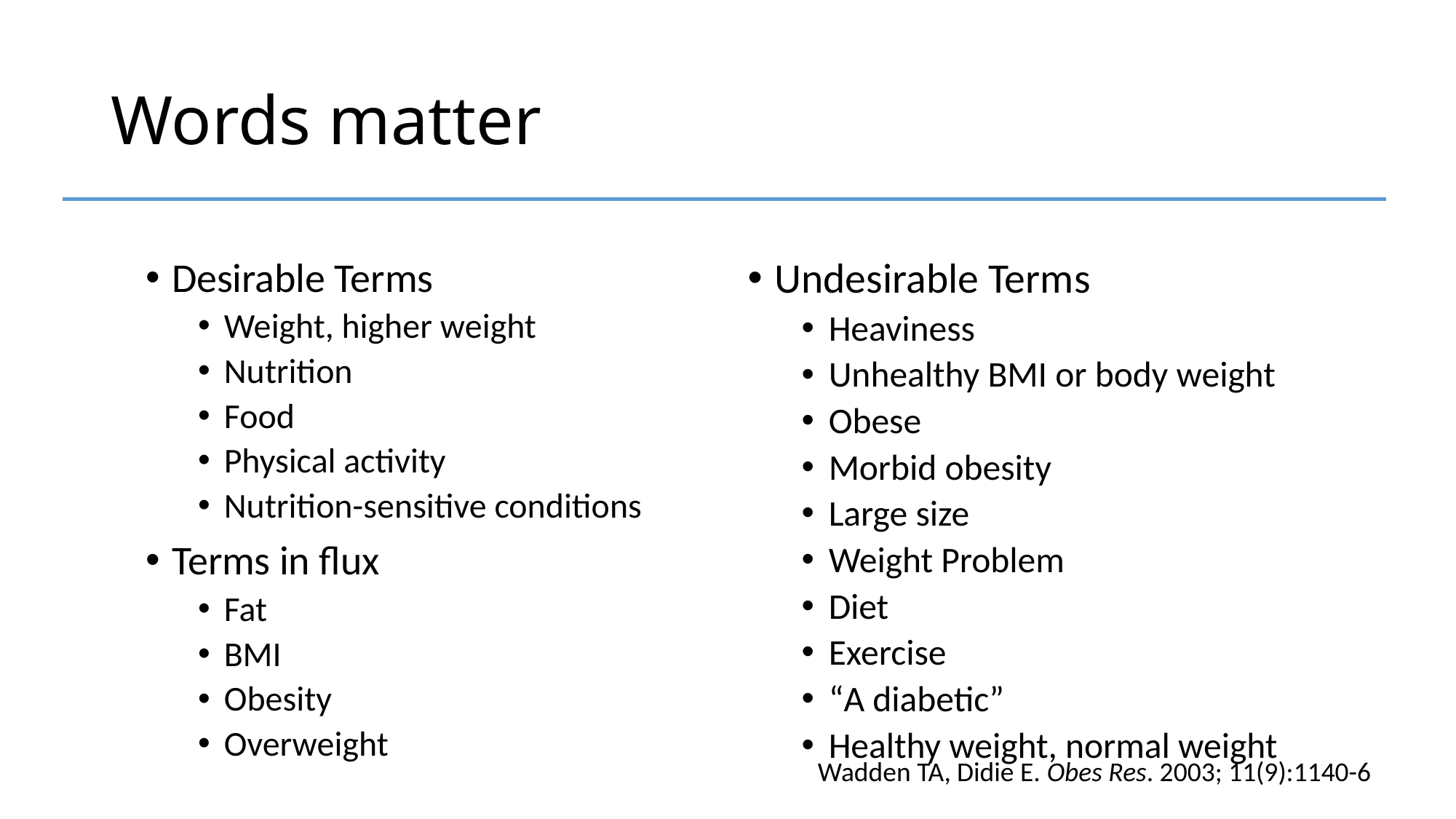

# Words matter
Desirable Terms
Weight, higher weight
Nutrition
Food
Physical activity
Nutrition-sensitive conditions
Terms in flux
Fat
BMI
Obesity
Overweight
Undesirable Terms
Heaviness
Unhealthy BMI or body weight
Obese
Morbid obesity
Large size
Weight Problem
Diet
Exercise
“A diabetic”
Healthy weight, normal weight
Wadden TA, Didie E. Obes Res. 2003; 11(9):1140-6

## Slide 29
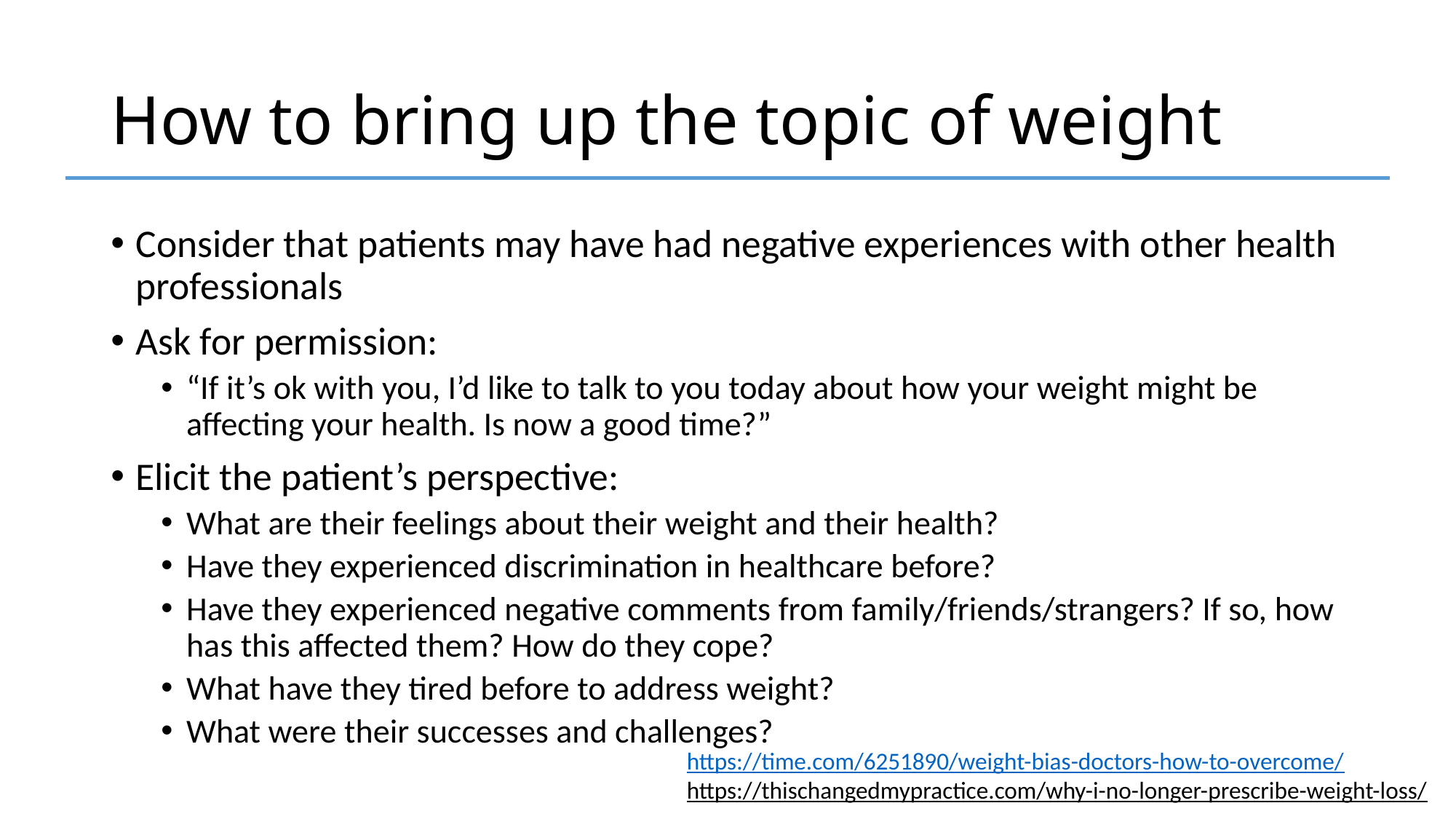

# How to bring up the topic of weight
Consider that patients may have had negative experiences with other health professionals
Ask for permission:
“If it’s ok with you, I’d like to talk to you today about how your weight might be affecting your health. Is now a good time?”
Elicit the patient’s perspective:
What are their feelings about their weight and their health?
Have they experienced discrimination in healthcare before?
Have they experienced negative comments from family/friends/strangers? If so, how has this affected them? How do they cope?
What have they tired before to address weight?
What were their successes and challenges?
https://time.com/6251890/weight-bias-doctors-how-to-overcome/
https://thischangedmypractice.com/why-i-no-longer-prescribe-weight-loss/

## Slide 30
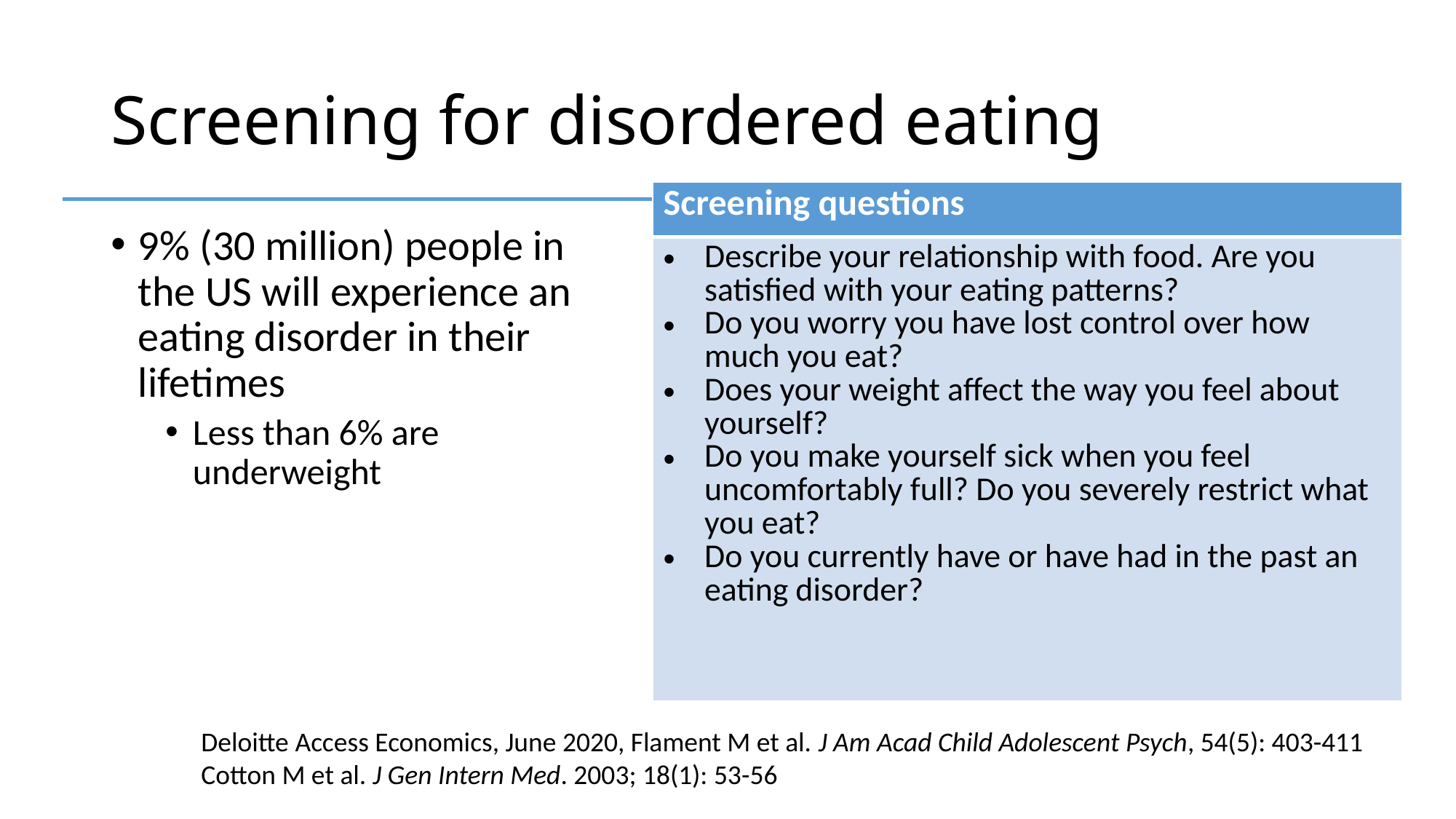

# Screening for disordered eating
| Screening questions |
| --- |
| Describe your relationship with food. Are you satisfied with your eating patterns? Do you worry you have lost control over how much you eat? Does your weight affect the way you feel about yourself? Do you make yourself sick when you feel uncomfortably full? Do you severely restrict what you eat? Do you currently have or have had in the past an eating disorder? |
9% (30 million) people in the US will experience an eating disorder in their lifetimes
Less than 6% are underweight
Deloitte Access Economics, June 2020, Flament M et al. J Am Acad Child Adolescent Psych, 54(5): 403-411
Cotton M et al. J Gen Intern Med. 2003; 18(1): 53-56

## Slide 31
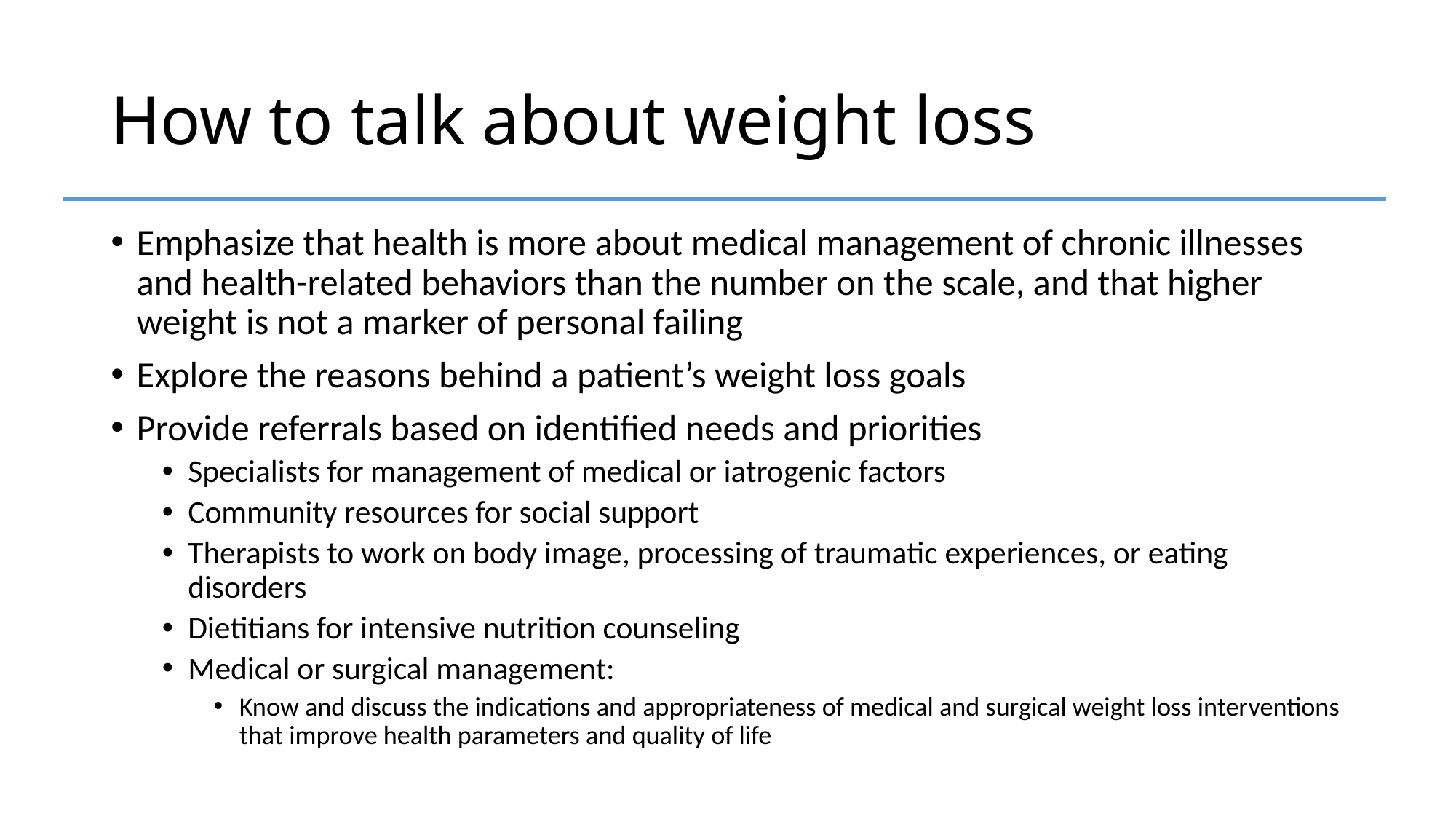

# How to talk about weight loss
Emphasize that health is more about medical management of chronic illnesses and health-related behaviors than the number on the scale, and that higher weight is not a marker of personal failing
Explore the reasons behind a patient’s weight loss goals
Provide referrals based on identified needs and priorities
Specialists for management of medical or iatrogenic factors
Community resources for social support
Therapists to work on body image, processing of traumatic experiences, or eating disorders
Dietitians for intensive nutrition counseling
Medical or surgical management:
Know and discuss the indications and appropriateness of medical and surgical weight loss interventions that improve health parameters and quality of life

## Slide 32
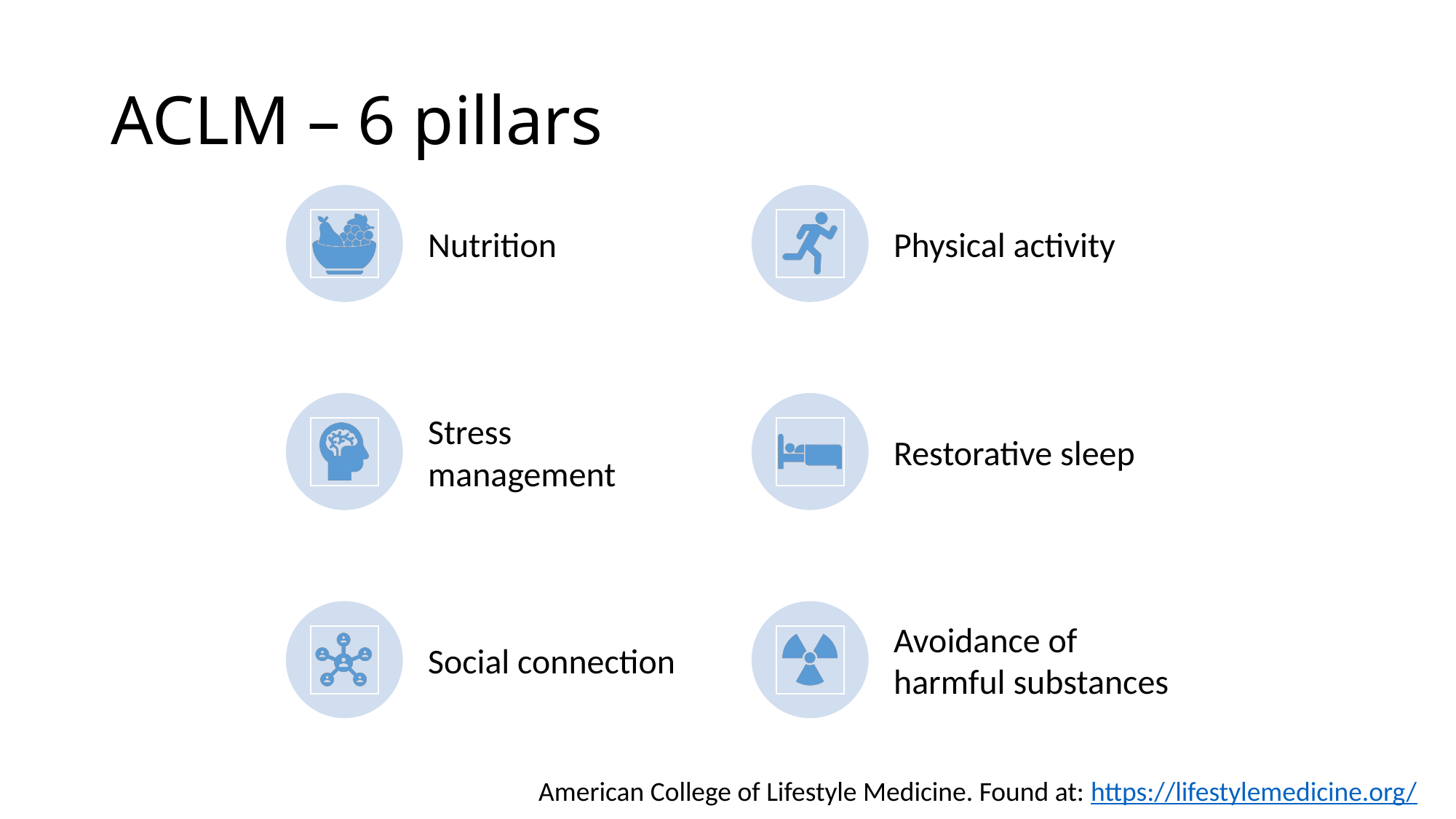

# ACLM – 6 pillars
American College of Lifestyle Medicine. Found at: https://lifestylemedicine.org/

## Slide 33
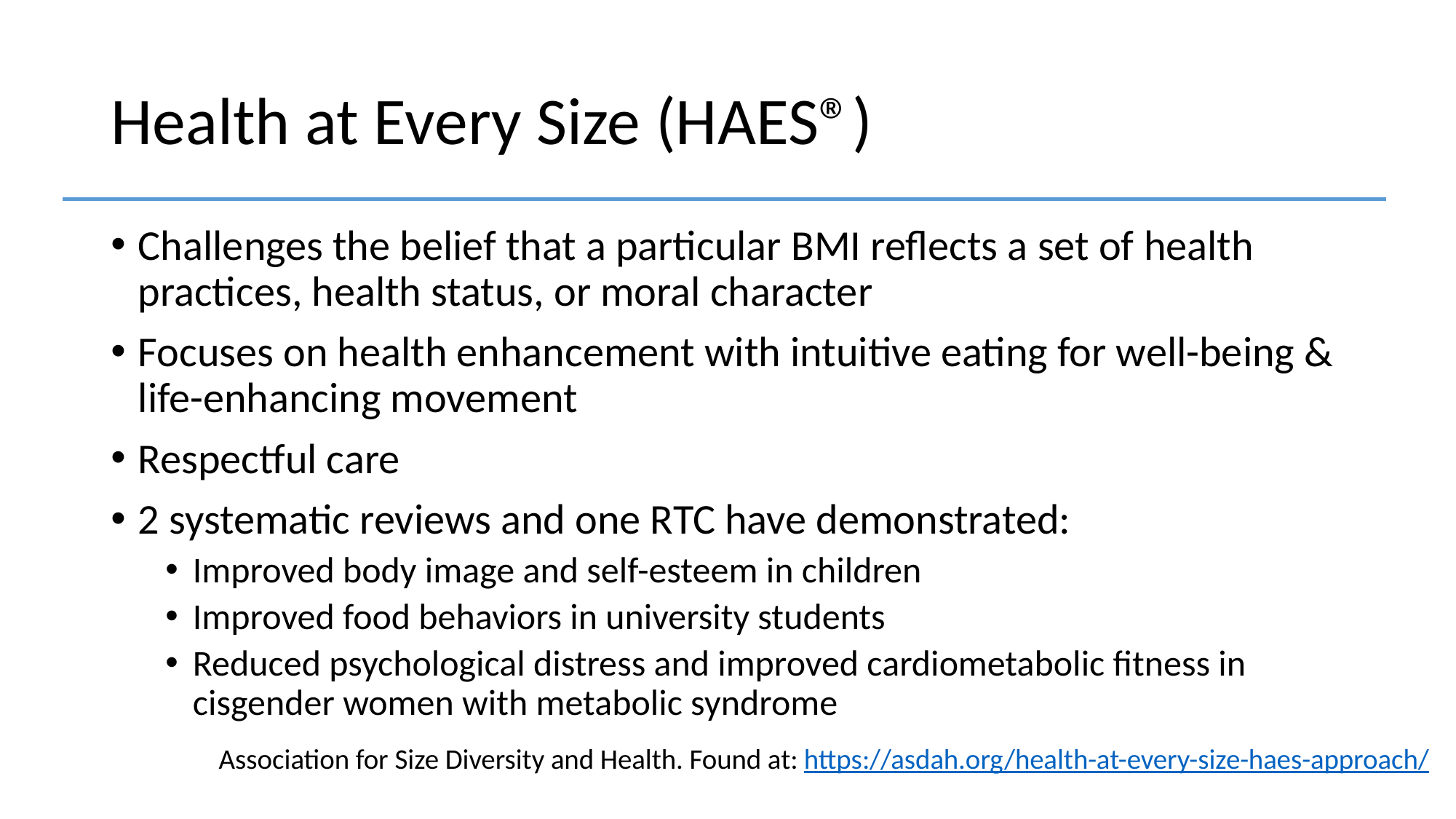

# Health at Every Size (HAES®)
Challenges the belief that a particular BMI reflects a set of health practices, health status, or moral character
Focuses on health enhancement with intuitive eating for well-being & life-enhancing movement
Respectful care
2 systematic reviews and one RTC have demonstrated:
Improved body image and self-esteem in children
Improved food behaviors in university students
Reduced psychological distress and improved cardiometabolic fitness in cisgender women with metabolic syndrome
Association for Size Diversity and Health. Found at: https://asdah.org/health-at-every-size-haes-approach/

## Slide 34
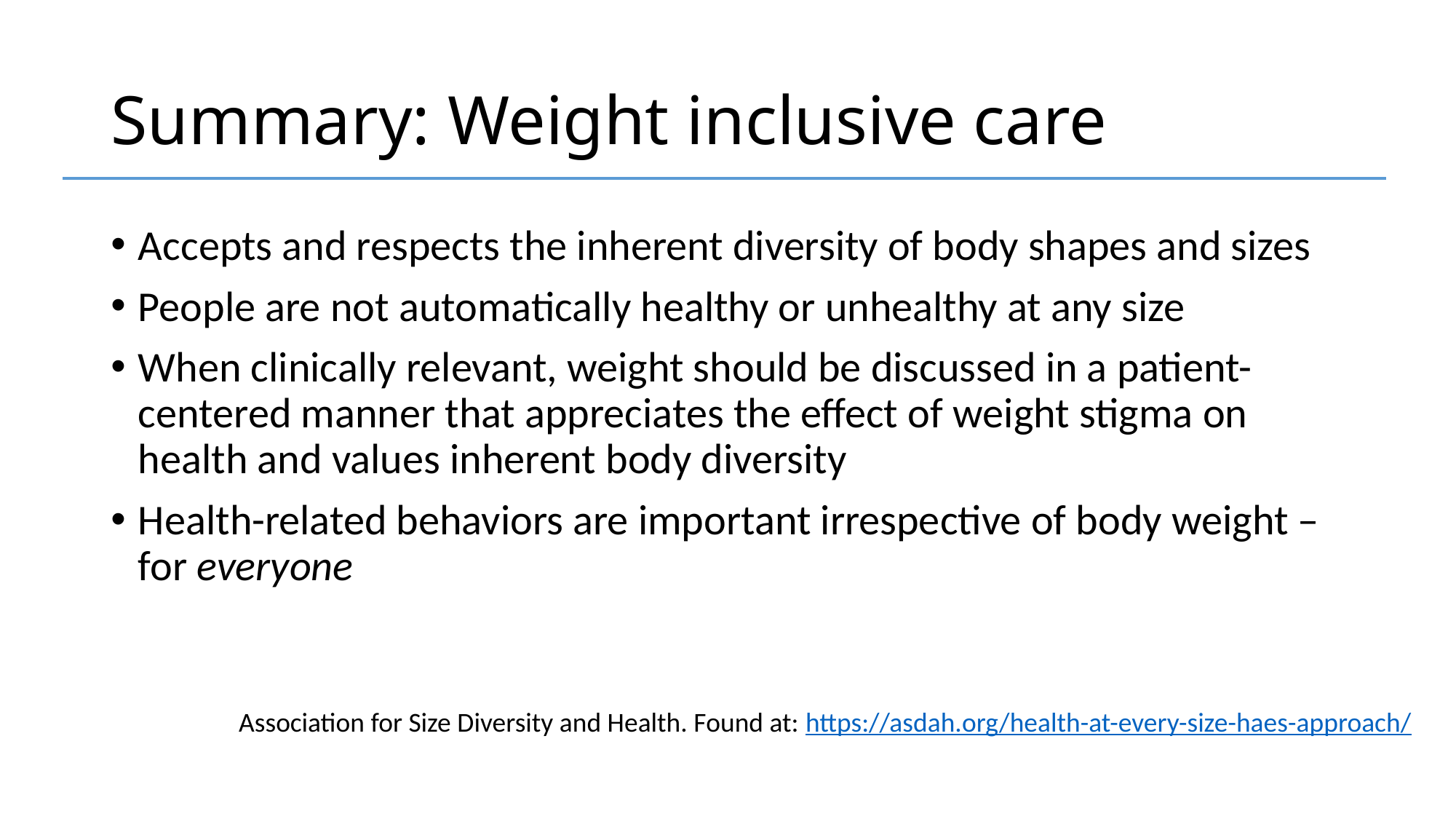

# Summary: Weight inclusive care
Accepts and respects the inherent diversity of body shapes and sizes
People are not automatically healthy or unhealthy at any size
When clinically relevant, weight should be discussed in a patient-centered manner that appreciates the effect of weight stigma on health and values inherent body diversity
Health-related behaviors are important irrespective of body weight – for everyone
Association for Size Diversity and Health. Found at: https://asdah.org/health-at-every-size-haes-approach/

## Slide 35
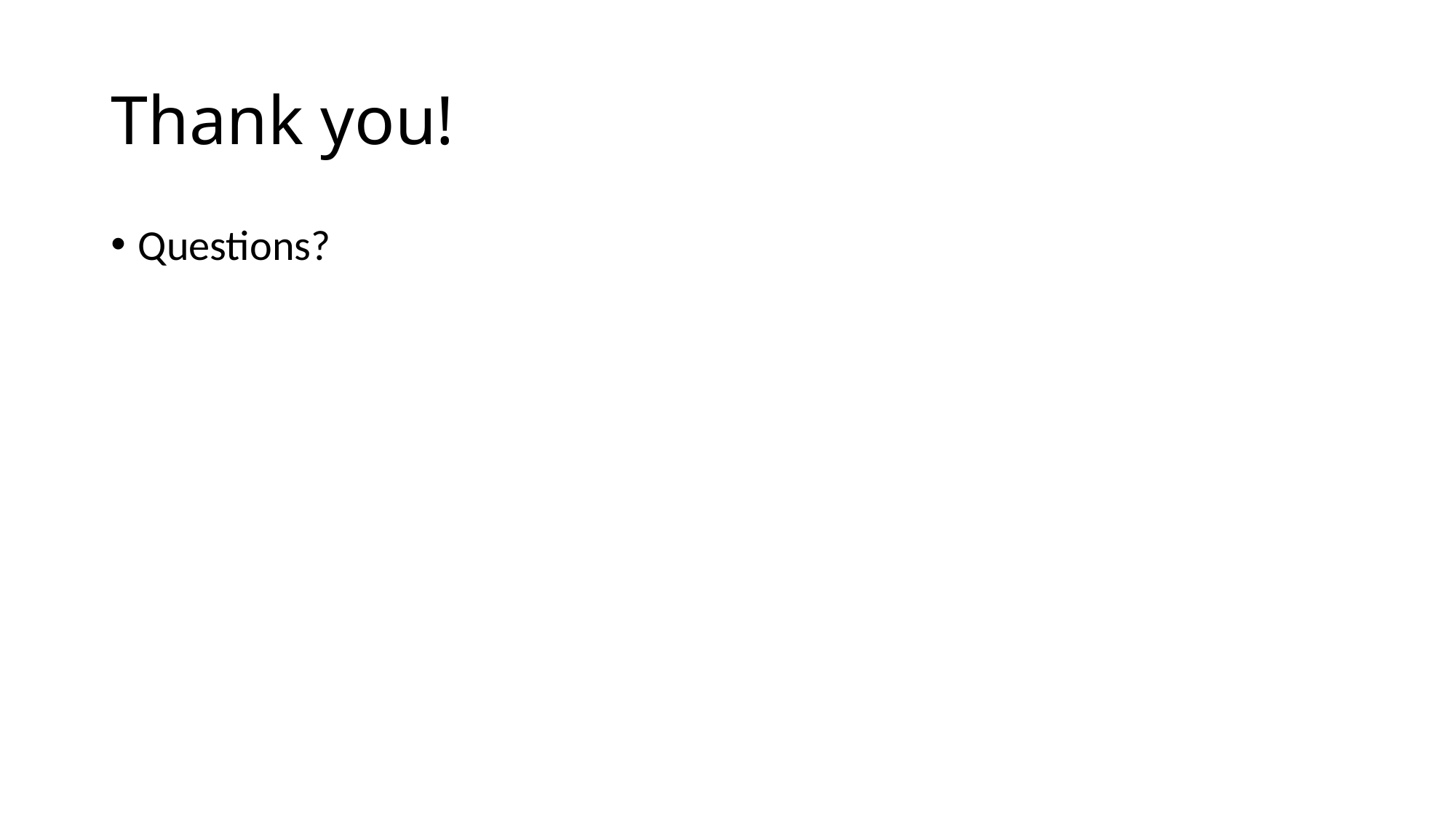

# Thank you!
Questions?
