# Supplementary material for: An Educational Session for Medical Students Exploring Weight Bias in Clinical Care Through the Lens of Body Diversity
Source: MedEdPORTAL. 2023 Sep 5;19:11342. doi: 10.15766/mep_2374-8265.11342 (PMC10477274; doi:10.15766/mep_2374-8265.11342)
Supplement: Supplementary file 1 — Understanding Body Diversity.pptxAddressing Weight Bias in Clinical Care.pptxFacilitator Guide.docxStudent Guide.docxMaterials Checklist and Timeline.docxQuiz.docxEvaluation Survey.docx [file mep_2374-8265.11342-s001.zip › C. Facilitator Guide.docx]

**Appendix C - Facilitator Guide**

**Weight bias**

***Instructions:*** *This facilitator guide should be used for both facilitator training and to help guide facilitators during the small group session. It should be distributed electronically prior to the small group session so that facilitators can review the detailed notes in advance. It is recommended that the training consists of reviewing this document with the facilitators and answering any questions. Electronic or paper copies can also be provided in the small group rooms on the day of the small group session along with attendance sheets, if applicable. The time allotted for this activity is* ***110-120 minutes (about 2 hours)****, with an estimated time designated at the top of each section.*

**Learning Objectives:** By the end of the session, students will be able to:

- Discuss critiques of the weight-centered paradigm
- Describe the multifactorial influences on body weight
- Describe the effect that weight bias can have on healthcare access, delivery, and outcomes
- Outline approaches to reduce weight stigma and incorporate body acceptance into the healthcare setting
- Discuss body diversity and body acceptance and their relevance to clinical care

**INTRODUCTIONS and COMMUNAL AGREEMENTS (10 minutes)**

1. Listen actively
2. Communicate in a nonjudgmental fashion
3. Do not be afraid to respectfully challenge each other by asking questions, but refrain from personal attacks
4. Use “I” statements—speak from your own experience
5. Maintain confidentiality
6. Reveal only what you feel comfortable revealing about yourself
7. There is no one “right” answer
8. The goal is not to agree; it is to gain a deeper understanding

***Facilitator Notes:***

*Read the agreements and ask if clarification is needed. Ask if anyone would like to add items. Elicit from the group members the steps they want to take if someone does not adhere to the agreements. Of note, this session asks students to openly discuss their own biases and presents challenges to the medical status quo with respect to weight. Dialogue and learning from one another are encouraged. Students will take turns reading the details of the case, and then dialogue in response to the prompting quesitons.*

**CASE DISCUSSION (100 minutes)**

In this case session, we will be exploring the medical history and care of a patient and dialoguing about common myths around weight gain and higher weight. Students may use outside references to supplement their learning during this discussion.

**Part 1 (10-15 mins)**

**Case Overview**

Jacqueline Williams is a 47-year-old cisgender woman with a history of well-controlled HIV on treatment and hypertension who presents for a new patient appointment in the HIV primary care clinic. She is transferring care from another provider. She was diagnosed with HIV approximately 9 months ago after her cisgender male partner was diagnosed. Her CD4 count at the time of her diagnosis was 231 with a viral load of 19,180. Her previous provider had started her on antiretroviral medications with a regimen of dolutegravir, lamivudine, and abacavir in a single tablet. She had been adherent to the medication and her last CD4 the month before had increased to 503 with an undetectable viral load. Her prior provider had also started her on hydrochlorothiazide, but she had not continued this because of the side effect of frequent urination.

Her main complaint today is of progressive fatigue over the past several months. She has a hard time getting out of bed in the morning and feels tired during the day. She does not report feeling excessively cold or warm, rashes, headaches, or polyuria/polydipsia.

She is seeking a new provider because she felt that her previous provider was not taking her concerns seriously.

**Prompting questions:**

1. What clinical problems have you identified with the information that you have so far?
2. Would you explore her reasons for changing providers in more detail? Why or why not? How would you ask?

***Facilitator Notes:***

*The patient is presenting with a concern of fatigue. She has a history of HIV and started treatment for this in the last 9 months, as well as hypertension. She also expresses some dissatisfaction with her prior care and undesirable side effects to her antihypertensive that led to discontinuation.*

*Students may have differing opinions about whether to inquire about why she felt her previous provider was not addressing her concerns seriously. However, this may be very useful information to explore. Does she have any hopes or expectations in changing providers? How can the provider now better meet her needs?*

**Part 2 (15-20 mins)**

Further history from the patient and review of the chart:

**Past Medical History**

HIV (see HPI for details)

--No history of opportunistic infections

Hypertension, diagnosed at the time of initial HIV visit, currently on no medications

Hyperlipidemia, diagnosed at the time of initial HIV visit, currently on no medications

Depression

--Briefly on citalopram about 5 years ago but had not tolerated well due to side effects

**Obstetric/Gyn History**

G3P2012

--Two vaginal deliveries, no complications, one miscarriage 1^st^ trimester

Periods every 4-8 weeks, irregular over last year, duration of 5 days with light to moderate flow

**Allergies**: NKDA

**Current medications**

Dolutegravir/abacavir/lamivudine 50/600/300mg one tablet by mouth once a day

Hydrochlorothiazide 25mg one tablet by mouth daily (prescribed but not taking)

**Family History:**

Mother: Gallstones

Father: Alcohol-related liver disease s/p orthotopic liver transplant

Daughters (x2) in their early 20s and have no known health conditions

**Current health risk factors:**

Nutrition: Does not eat red meat or pork. Rarely eats fried food, tries to eat mostly baked or grilled fish/chicken. Trying to incorporate more vegetables and fruits. Beverages: mostly water and iced tea. Coffee with cream and sugar every morning

Physical activity: Walks to and from bus stops and stays active at her job. More recently has incorporated longer walks by getting off the bus a few stops before her destination

Smoking history: 7 cigarettes per day since age 24; recently quit for about 2 months but then resumed 1 month ago in the setting of increased stress

Alcohol: 3-4 drinks per day (wine), 3-4 times per week. CAGE: 2 (Cut down- yes; Annoyed- no; Guilty- yes; Eye-Opener- no)

Other substances: None/never

Sleep pattern: Sleeps about 5-7 hours per night. Often stays up later than she thinks she should and about 3-4 nights out the week has a hard time falling asleep. Sleep is generally uninterrupted but frequently feels tired when she wakes up. No reports of snoring or apneic episodes. No caffeine after 2:00pm

**Social History:**

Sexual history: Sex with cisgender men

--Practices oral and vaginal intercourse

--Uses condoms 100% for vaginal sex since dx of HIV

--One partner over last 4 years; he is living with HIV and in care

--No history of other sexually transmitted infections

Personal history: Born in [location of medical school], has always lived here. Formerly married, had 2 children, divorced about 10 years ago.

Occupation: Employed as home health aide

Housing: Lives with her partner, rents a one-bedroom apartment in [location of medical school], stable housing

HITS score: 7 (H- never; I- sometimes; T- never; S- often)

**Prompting questions:**

1. *Myth*: Anyone can control weight with diet and exercise alone.
   1. Why is this a myth? From where do you think this myth originated?
   2. How can this myth harm patients in the context of clinical care?
   3. Given this additional information for this case, what factors outside of food and physical activity do you identify in her history that can impact her body weight?

***Facilitator Notes:***

*Ms. Williams has several factors that may have contributed to weight gain:*

- *Health-related behaviors (alcohol intake, recent 2 months of smoking cessation)*
- *Mental health concerns (history of depression, potential high stress levels – financial insecurity, HITS of 7, experiencing stigma of HIV)*
- *Food insecurity, transportation*
- *Inadequate sleep*
- *Physical factors (fatigue)*
- *Medical factors (started on treatment for HIV at a relatively low CD4 count, which can lead to weight gain with suppressed viral load; irregular periods suggesting possible peri-menopausal state)*
- *Medications (Antiretroviral therapy- dolutegravir has been associated with weight gain in excess of what is expected from suppressed viral load)*

*The myth that anyone can control their weight with diet and exercise alone fails to consider the other medical and social factors that influence body weight. It also assumes that the weight itself is the essential clinical problem. But as discussed, weight gain may be a proxy or a result of precursor metabolic disturbances, and weight in and of itself may or may not be medically consequential. As discussed in the lecture, many people who fall into the WHO category of “overweight” or “obese” are metabolically healthy and not at risk for adverse clinical outcomes.*

*Medical conditions, such as hypothyroidism, possible peri-menopausal state, and depression can lead to low energy and decreased physical activity. In addition, the reset of metabolism after weight gain can make it more difficult to lose weight even with relatively severe calorie restriction. Patients may meet plateaus in weight loss due to feedback physiologic systems that maintain body homeostasis and inhibition of leptin, a protein cytokine secreted and stored in fat cells that modulates hunger.*

*Furthermore, not everyone has equitable access to the tools for a sustainable health-related behaviors, particularly those who live with disabilities or with a physical infrastructure that makes health-related activities inaccessible, and health-promoting foods inaccessible, more expensive, less advertised, and less appealing than the alternatives.*

**Part 3 (15-20 mins)**

|  |  |  |
| --- | --- | --- |
| **Visit Vitals** | | |
| • | Blood pressure | 155/80; repeat manual measurement 146/84 |
| • | Pulse | 68 |
| • | Temperature | 98.1 °F (36.7 °C) (Oral) |
| • | Respiratory rate | 14 |
| • | Height | 1.803 m (5' 11") |
| • | Weight | 102.4 kg (225 lb 12.8 oz) |
| • | BMI  Waist circumference | 31.49 kg/m2  Not done |

Other than the note of a flat affect, the rest of the exam shows normal findings

**Recent labs:**

Na 140 WBC 5.4

K 4.4 Hemoglobin 13.6 HCT 41.7

Cl 102 Platelets 197

HCO3 24 **Total cholesterol 284**

BUN 8 **Triglycerides 194**

Cr 0.7 HDL 56

Fasting Glucose 93 **LDL 189**

**AST 51**

**ALT 48**

Tot Protein 8.6 HBA1c 5.7

Albumin 4.7 TSH 1.150

Alkaline Phosphatase 66

Total bilirubin 0.4

**Prompting questions:**

1. Consider the limitations discussed with BMI in the lecture. What classification does this patient fall under based on the WHO classification? How do you consider BMI playing a role (or not) in the clinical assessment for this patient? Is it helpful?
2. *Myth*: People with higher weight lack motivation and self-control.
   1. From where do you think this myth originated?
   2. Have you ever considered your own biases with respect to larger bodies? What biases have you noted in yourself and/or others?
   3. How can these biases harm patients in the context of clinical care?

***Facilitator Notes:***

*The international classification of adult “underweight,” “overweight,” and “obesity” according to BMI*

*Underweight <18.5*

*Normal range 18.5-25.9*

*Overweight ≥25*

*Obese ≥30*

*Class 1 30-34.9*

*Class 2 35-39.9*

*Class 3 ≥40*

*Students may debate the clinical utility of BMI assessment. Within clinical medicine, there is still an active debate as to its utility, and many entities, including the USPTF, recommend BMI screening assessment followed by referral to intensive behavioral interventions for BMI >30. Patients with BMI >35 (Class 2 & 3) may be candidates for weight loss treatments, which have been shown to have clinical benefits, including reversal of diabetes and hypertension. However, as discussed, BMI is very limited in its utility as a screening tool at the level of the individual for metabolic illnesses and as a tool in research and leads to an inappropriately intense focus on weight and weight loss.*

*The pathologizing/medicalization of weight is in some ways reflective of an underlying bias that higher weight is a moral failing in a culture that has disdain for larger bodies. The myth that people with higher weight lack motivation and self-control is based on this false underlying belief and highlights intense amount of bias around weight in our society. Despite our best intentions we often make unconscious judgments about people based on their appearance. This is especially true in the case of people with higher weight. Research shows that healthcare providers have the pre-conceived impression that patients with higher weight lack motivation and self-control, perceiving them as lazy. This can have profound effects on the way that patients are treated in the healthcare setting, leading to dismissal of their concerns, less effort to work with patients on behavior change, and negative attitudes and comments about weight. In this case, from obtaining a full history, we can see that the patient made significant health-related changes by adopting new behaviors. She does not lack motivation, but rather, deserves support and attention to her medical problems, medications, social factors, and improved patient-provider communication/trust.*

**Part 4 (15-20 mins)**

Before proceeding with lifestyle modification counseling, you ask some questions to better understand her perspective.

**Patient’s perspective:**

The patient felt that her previous provider attributed all her concerns to excess weight. She also felt uncomfortable in the previous office because one of the medical assistants once gave her the unsolicited advice that all she needed was “a little bit of willpower” to “curb the fat”. She has been worried about her weight gain; she has gained 25 pounds since starting HIV medications. Generally, she does not like taking medications and would prefer more natural remedies when possible. She is absolutely dedicated to taking her HIV medication, however, and never misses a dose.

She would like to be able to lose the weight she gained but is not sure what else she can do as nothing has seemed to help so far. It is getting frustrating having people comment about her weight and tell her that all her problems are related to this. On the other hand, she is glad that she did not lose weight in the setting of her HIV diagnosis, because she is afraid that people will find out about her HIV diagnosis and tell everyone that she is wasting away because of it.

She also reports feeling depressed. Things are not going well with her partner. She blames him for giving her HIV. He recently lost his job and is looking for a new one. It is stressful having to support them both and helping her daughter pay for college. She has had to take on more patients and work longer hours, making meal preparation very difficult. She has felt too ashamed to disclose her new HIV diagnosis with her friends or family, and this has been troubling her. She wishes she had someone she could talk to other than her boyfriend.

**Prompting questions**

1. What thoughts/feelings emerge as you hear the entirety of her history and life experiences?
2. *Myth*: Forcing people to take responsibility for their weight is the best way to facilitate behavior change.
   1. From where do you think this myth originated?
   2. How can this myth harm patients in the context of clinical care? How is it relevant in this case?

***Facilitator Notes:***

*She has several issues that are impacting her life currently, including high stress levels, depression, and financial insecurity.*

*She is seeking healthier behaviors (trying to eat more vegetables, salads, and be physically active with walking more) but states that her work schedule and psychosocial factors make this difficult. It is important to recognize that she has already been motivated to make changes to be healthier and to provide positive reinforcement for this. At the same time, there are many other issues that take precedence.*

*Mental health concerns (stress and depression) need to be addressed and prioritized. Students may feel a sense of clarity or empathy in understanding her perspective. However, they may also feel a sense of frustration, or helplessness. As medical providers, how can we possibly take on the complex social factors and stigma that affect our patients? How can we begin to alleviate her suffering when we seemingly have little control over her relationship, her income, her living situation, societal sources of stigma related to HIV and weight? They may indicate that there are appropriate referrals that can be made at this time and using motivational interviewing techniques can be helpful too. It is important to note that sometimes simply allowing the patient to express their perspective, to listen to it and to express your understanding, can be therapeutic in and of itself. It also establishes trust going forward, so that the patient may be more likely to follow through on your advice and recommendations in the future.*

*The myth that forcing people to take responsibility will facilitate behavior change is one that is accepted in U.S. culture. Weight bias is among the most prevalent and pervasive types of bias in our society. While some people may respond to a fear-generating response, most will not. Stigmatizing or shaming patients, particularly in a healthcare setting, more frequently leads to resignation, hopelessness and avoidance, rather than motivation. In fact, studies show that weight discrimination is associated with earlier mortality, suggesting the powerful harm that weight stigma can perpetuate. Furthermore, it is important to emphasize that elevated BMI does not automatically mean that behavior change is needed. Weight is not a behavior. Given the diversity of body types, people with elevated BMI may have active lifestyles, eat health-promoting foods, be physically fit, and be at low risk for metabolic or other complications. The patient in this case was further motivated to change providers because of the comments and treatment around her weight she had experienced prior. In other cases, patients may avoid or disengage from care. And her experienced stress and discrimination may be intersectional with gender, race/ethnicity, and illness scripts.*

*In this case, there is evidence of metabolic dysfunction, with elevated blood pressure, elevated cholesterol, and hepatic steatosis. These are clear parameters that can be treated and followed over time, and a large part of that treatment includes lifestyle factors in addition to medical management. Later in the course, we will learn the basics of motivational interviewing, a technique that can help clinicians motivate patients for behavior change and facilitate* ***positive*** *feelings and action.*

**Part 5 (15 mins)**

After obtaining the history and physical and establishing rapport with the patient, you refer her for psychosocial support with medical case management. You note the important steps she has taken to improve her health, and plan for close follow-up in one month. At that time, you agree to discuss in more detail management of her hypertension, hyperlipidemia, and unintentional weight gain. You also discuss with her the option of changing her antiretroviral medications, but she is not ready to make a change at this time because her current regimen is convenient and has worked well for her.

One month later, you are preparing to see the patient for follow-up.

**Prompting questions:**

1. Putting everything together, what recommendations do you have for the patient?
2. How might an approach based on body diversity and acceptance facilitate your discussions with the patient surrounding her medical issues and her concerns about her weight? Do you have concerns about this approach? What elements of this approach do you view as most valuable to patient care?

***Facilitator’s Notes***

*Recommendation: Remember the 6 pillars from the American College of Lifestyle medicine: Health-promoting foods, better sleep, social support, reduction of alcohol and tobacco use, stress relief, and regular physical activity. However, remember that not everyone is able to fully integrate lifestyle changes related to their social circumstances, and it is important for us to elicit detailed social histories and take them into consideration.*

*Use open-ended questions to begin the discussion.*

- *We have identified a few issues that I’m concerned about for your health. What are your thoughts about these conditions?*
- *What have you tried in the past to improve your health?*
- *Which of these lifestyle factors that we’ve discussed today do you feel you’d like to address first?*
- *What do you feel motivated to work on this visit?*
- *If the patient identifies she wants to work on weight loss – Elicit her perspective. What are your feelings about your weight? What is your motivation for weight loss? Have you experienced stigma/discrimination? If so, how has this affected you? Have you tried to lose weight before? Tell me about your experiences with losing weight in the past. Describe your relationship with food.*
- *Consider screening for eating disorders if you are concerned based on the patient’s perspective: Are you satisfied with your eating patterns? Do you worry you have lost control over your eating? Do you restrict what you eat? Do you make yourself sick when you feel uncomfortably full? Do you currently have or have had in the past an eating disorder?*

*Additional helpful recommendations and referrals:*

- *Change medications that contribute to weight gain*
- *Antihypertensive treatment*
- *Statin therapy*
- *Medication for depression*
- *Medical case management / social work*
- *Mental health care*
- *Dietitian*
- *Support groups*

*Students may be curious about the indications for weight loss treatments, including medications and bariatric surgery. These clinical criteria are covered in other courses in the curriculum and are not the focus of this session. In this case, there are important medical, mental health, and social factors that take precedence over moving directly towards weight loss treatments. The decision to proceed with treatment is an individualized one. Students should refer to the lecture for advice on how to approach bringing up the topic of weight loss when weight is a factor influencing health conditions. This can include asking for permission, eliciting the patient’s perspective about their experiences with weight, and talking with them about available treatment options, benefits and risks, and parameters that can be followed over time, including blood pressure control, A1c, cholesterol, hepatic panel tests, and an internal sense of wellness.*

*When prompted, students will likely bring up concerns about body acceptance in clinical care, primarily because they have frequently heard and learned that “obesity” is a risk factor for various disease states. They may express concern that minimizing the potential adverse health effects of elevated weight and metabolically active adipose tissue. Body acceptance and weight-inclusive care, however, do not advocate for ignoring weight when it has clinical relevance, but rather, acknowledge the reality of body diversity, center general well-being instead of achieving “normal weight,” and encourage people to have a positive attitude toward their bodies with the understanding that keeping it as healthy as possible, irrespective of weight, size, or ability, means caring for it holistically over the long-term and honoring its functions. In this way, weight-inclusive care aims to reduce stigma, enhance patient-provider communication, improve health outcomes, and increase access to high-quality clinical care.*

**Case resolution and wrap-up (15-20 mins)**

Over the next several visits, you recommend sleep hygiene, reducing salt, carbohydrate and sugar intake, and regular physical activity over the next 6 months. You suggest changing her HIV medication and starting a new antihypertensive. She is ready to change her HIV medication but reluctant to restart an antihypertensive in light of her experience prior. She would like to try to manage her blood pressure with lifestyle, which is also more consistent with her desire to use natural remedies rather than medications. She starts rosuvastatin for hyperlipidemia.

You refer the patient for mental health services. She does not want to start an anti-depressant medication since she is worried about the side effects. With encouragement and support from her medical case manager, she successfully discloses her HIV status to her sister and her older daughter, and they are very supportive, which helps her with the burden of HIV stigma. Her relationship with her partner ends on mutual terms and he moves out. She continues to take on extra work as a home health aide and at times her job remains quite stressful, but she has a few patients for whom she regularly cares and feels that she gets a sense of purpose from her work.

Over the next year, the patient meets regularly with a trained Health At Every Size® dietitian, and they work together to create meal plans within her budget that reduce salt, sugars and carbohydrates and increase potassium-rich foods, fresh fruits and vegetables, working to be more attuned to her eating habits and eating more things that she likes. She starts a regular exercise program 2-3 times a week by going to the local gym to swim, which she always had enjoyed. She reports feeling more energized, fit, and hopeful. At 18 months after her first visit, her vital sign and lab parameters are:

- BP 136/70
- Triglycerides 168
- LDL 145, HDL 53
- A1c 5.5
- ALT 21

**Prompting questions:**

1. What are some of the factors in the case that led to improved clinical outcomes in her blood pressure and labs?
2. What are the top three take-home messages that you learned from the pre-assignments and the case discussion?

***Facilitator Notes:***

*Possible factors for positive outcome:*

- *Addressing medication changes*
- *Reduction of HIV stigma*
- *Treatment of depression*
- *Enhanced trust in patient-provider relationship*
- *Mental health counseling*
- *Regular consultation with registered dietitian*
- *Support through medical case management*
- *Empowerment/motivation rather than shaming*
- *Incorporating patient’s values and wishes into the treatment plan*
- *Physical activity regimen that she enjoys and is accessible/affordable*
- *Ending of stressful relationship*
- *Finding meaning in work*
- *Medication management*

*Possible take-home messages:*

- *Weight stigma is harmful*
- *Debunking myths around weight will help us better care for our patients*
- *Focusing on body acceptance, motivation and empowerment, rather than shaming or stigmatizing can be therapeutic*
- *A global approach to the many factors that affect higher weight is more effective than strictly focusing on lifestyle changes or numbers on the scale alone*
- *Fostering partnerships to create treatment plans that are acceptable to both patient and provider is a key element of successful outcomes*
- *Health-promoting nutrition, physical activity, stress reduction, sleep hygiene, and social support are key recommendations for healthy living for* ***all*** *patients across the spectrum of body shape and size*

*Students may note that weight is not included at the end of the case. In weight-inclusive care, weight in and of itself is not the goal (unless this is an expressed personal goal of the patient)—the goals are improved health parameters and internal sense of well-being. It is also very important to point out that part of this patient’s presentation included weight gain and metabolic changes, but not all patients with elevated weight have metabolic dysfunction; and furthermore, the metabolic dysfunction in part may be causing the weight gain rather than the other way around. While high weight in the extreme can cause direct health effects (such as obstructive sleep apnea), having higher weight does not automatically equate to being sick. Incorporating body diversity and acceptance into medical care means helping patients focus on health above weight or body shape. This movement is very much grounded in empathy, partnership, and patient agency– seeing the unique strengths in every patient and working with them to improve and sustain healthy outcomes.*

*Contributors to this guide: Dr. Michelle DallaPiazza, Dr. Joy Cox, Dr. Dhvani Doshi, Dr. Victor Cueto, Dr. Aleksey Tentler. Rutgers New Jersey Medical School*
