# Supplementary material for: An Educational Session for Medical Students Exploring Weight Bias in Clinical Care Through the Lens of Body Diversity
Source: MedEdPORTAL. 2023 Sep 5;19:11342. doi: 10.15766/mep_2374-8265.11342 (PMC10477274; doi:10.15766/mep_2374-8265.11342)
Supplement: Supplementary file 1 — Understanding Body Diversity.pptxAddressing Weight Bias in Clinical Care.pptxFacilitator Guide.docxStudent Guide.docxMaterials Checklist and Timeline.docxQuiz.docxEvaluation Survey.docx [file mep_2374-8265.11342-s001.zip › D. Student Guide.docx]

**Appendix D - Student Guide**

**Weight bias**

***Instructions:*** *This guide is meant to be distributed to the students at the start of the small group session. It can be provided electronically on an educational portal or in paper format in the small group room. There are page breaks between each section, and students are encouraged not to look ahead in the guide. Students will take turns reading the details of the case, and then engage in dialogue in response to the prompting questions. The time allotted for this activity is* ***110-120 minutes (about 2 hours)****, with an estimated time designated at each section.*

**Learning Objectives:** By the end of the session, students will be able to:

- Discuss critiques of the weight-centered paradigm
- Describe the multifactorial influences on body weight
- Describe the effect that weight bias can have on healthcare access, delivery, and outcomes
- Outline approaches to reduce weight stigma and incorporate body acceptance into the healthcare setting
- Discuss body diversity and body acceptance and their relevance to clinical care

**INTRODUCTIONS and COMMUNAL AGREEMENTS (10 minutes)**

1. Listen actively
2. Communicate in a nonjudgmental fashion
3. Do not be afraid to respectfully challenge each other by asking questions, but refrain from personal attacks
4. Use “I” statements—speak from your own experience
5. Maintain confidentiality
6. Reveal only what you feel comfortable revealing about yourself
7. There is no one “right” answer
8. *The goal is not to agree; it is to gain a deeper understanding*

**CASE DISCUSSION (100 minutes)**

In this case session, we will be exploring the medical history and care of a patient and dialoguing about common myths around weight gain and higher weight. Students may use outside references to supplement their learning during this discussion.

**Part 1 (10-15 mins)**

**Case Overview**

Jacqueline Williams is a 47-year-old cisgender woman with a history of well-controlled HIV on treatment, hypertension, and an elevated hemoglobin A1c who presents for a new patient appointment in the HIV primary care clinic. She is transferring care from another provider. She was diagnosed with HIV approximately 9 months ago after her cisgender male partner was diagnosed. Her CD4 count at the time of her diagnosis was 231 with a viral load of 19,180. Her previous provider had started her on antiretroviral medications with a regimen of dolutegravir, lamivudine, and abacavir in a single tablet. She had been adherent to the medication and her last CD4 the month before had increased to 503 with an undetectable viral load. Her prior provider had also started her on hydrochlorothiazide, but she had not continued this because of the side effect of frequent urination.

Her main complaint today is of progressive fatigue over the past several months. She has a hard time getting out of bed in the morning and feels tired during the day. She does not report feeling excessively cold or warm, rashes, headaches, or polyuria/polydipsia.

She is seeking a new provider because she felt that her previous provider was not taking her concerns seriously.

**Prompting questions:**

1. What clinical problems have you identified with the information that you have so far?
2. Would you explore her reasons for changing providers in more detail? Why or why not? How would you ask?

**Part 2 (15-20 mins)**

Further history from the patient and review of the chart:

**Past Medical History**

HIV (see HPI for details)

--No history of opportunistic infections

Hypertension, diagnosed at the time of initial HIV visit, currently on no medications

Hyperlipidemia, diagnosed at the time of initial HIV visit, currently on no medications

Elevated hemoglobin A1c of 6.2 one month ago

Depression

--Briefly on SSRI citalopram about 5 years ago but had not tolerated well due to side effects

**Obstetric/Gyn History**

G3P2012

--Two vaginal deliveries, no complications, one miscarriage 1^st^ trimester

Periods every 4-8 weeks, irregular over last year, duration of 5 days with light to moderate flow

**Allergies**: NKDA

**Current medications**

Dolutegravir/abacavir/lamivudine 50/600/300mg one tablet by mouth once a day

Hydrochlorothiazide 25mg one tablet by mouth daily (prescribed but not taking)

**Family History:**

Mother: Gallstones

Father: Alcohol-related liver disease s/p orthotopic liver transplant

Daughters (x2) in their early 20s and are healthy

**Current health risk factors:**

Nutrition: Does not eat red meat or pork. Rarely eats fried food, tries to eat mostly baked or grilled fish/chicken. Trying to incorporate more vegetables and fruits. Beverages: mostly water and iced tea. Coffee with cream and sugar every morning

Physical activity: Walks to and from bus stops and stays active at her job. More recently has incorporated longer walks by getting off the bus a few stops before her destination

Smoking history: 7 cigarettes per day since age 24; recently quit for about 2 months but then resumed 1 month ago in the setting of increased stress

Alcohol: 3-4 drinks per day (wine), 3-4 times per week. CAGE: 2 (Cut down- yes; Annoyed- no; Guilty- yes; Eye-Opener- no)

Other substances: None/never

Sleep pattern: Sleeps about 5-7 hours per night. Often stays up later than she thinks she should and about 3-4 nights out the week has a hard time falling asleep. Sleep is generally uninterrupted but frequently feels tired when she wakes up. No reports of snoring or apneic episodes. No caffeine after 2:00pm

**Social History:**

Sexual history: Sex with cisgender men

--Practices oral and vaginal intercourse

--Uses condoms 100% for vaginal sex since dx of HIV

--One partner over last 4 years; he is living with HIV and in care

--No history of other sexually transmitted infections

Personal history: Born in [location of medical school] and has always lived here. Formerly married, had 2 children, divorced about 10 years ago.

Occupation: Employed as home health aide

Housing: Lives with her partner, rents a one-bedroom apartment in Newark, stable housing

HITS score: 7 (H- never; I- sometimes; T- never; S- often)

**Prompting questions:**

1. *Myth*: Weight can be controlled with diet and exercise alone.
   1. Why is this a myth? From where do you think this myth originated?
   2. How can this myth harm patients in the context of clinical care?
   3. Given this additional information for this case, what factors outside of food and physical activity do you identify in her history that can impact her body weight?

**Part 3 (15-20 mins)**

|  |  |  |
| --- | --- | --- |
| **Visit Vitals** | | |
| • | Blood pressure | 155/80; repeat manual measurement 146/84 |
| • | Pulse | 68 |
| • | Temperature | 98.1 °F (36.7 °C) (Oral) |
| • | Respiratory rate | 14 |
| • | Height | 1.803 m (5' 11") |
| • | Weight | 102.4 kg (225 lb 12.8 oz) |
| • | BMI  Waist circumference | 31.49 kg/m2  Not done |

Other than the note of a flat affect, the rest of the exam shows normal findings

**Recent labs:**

Na 140 WBC 5.4

K 4.4 Hemoglobin 13.6 HCT 41.7

Cl 102 Platelets 197

HCO3 24 **Total cholesterol 284**

BUN 8 **Triglycerides 194**

Cr 0.7 HDL 56

Fasting Glucose 93 **LDL 189**

**AST 51**

**ALT 48**

Tot Protein 8.6 **HBA1c 6.2**

Albumin 4.7 TSH 1.150

Alkaline Phosphatase 66

Total bilirubin 0.4

**Prompting questions:**

1. Consider the limitations discussed with BMI in the lecture. What classification does this patient fall under based on the WHO classification? How do you consider BMI playing a role (or not) in the clinical assessment for this patient? Is it helpful?
2. *Myth*: People with higher weight lack motivation and self-control.
   1. From where do you think this myth originated?
   2. Have you ever considered your own biases with respect to larger bodies? What biases have you noted in yourself and/or others?
   3. How can these biases harm patients in the context of clinical care?

**Part 4 (15-20 mins)**

Before proceeding with lifestyle modification counseling, you ask some questions to better understand her perspective.

**Patient’s perspective:**

The patient felt that her previous provider attributed all of her concerns to excess weight. She also felt uncomfortable in the previous office because one of the medical assistants once gave her the unsolicited advice that all she needed was “a little bit of willpower” to “curb the fat”. She has been worried about her weight gain; she has gained 25 pounds since starting HIV medications. Generally, she does not like taking medications and would prefer more natural remedies when possible. She is absolutely dedicated about taking her HIV medication, however, and never misses a dose.

She would like to be able to lose the weight she gained but is not sure what else she can do as nothing has seemed to help so far. It is getting frustrating having people comment about her weight and tell her that all of her problems are related to this. On the other hand, she is glad that she did not lose weight in the setting of her HIV diagnosis, because she is afraid that people will find out about her HIV diagnosis and tell everyone that she is wasting away because of it.

She also reports feeling depressed. Things are not going well with her partner. She blames him for giving her HIV. He recently lost his job and is looking for a new one. It is stressful having to support them both and helping her daughter pay for college. She has had to take on more patients and work longer hours, making meal preparation very difficult. She has felt too ashamed to disclose her new HIV diagnosis with her friends or family, and this has been troubling her. She wishes she had someone she could talk to other than her boyfriend.

**Prompting questions**

1. What thoughts/feelings emerge as you hear the entirety of her history and life experiences?
2. *Myth*: Forcing people to take responsibility for their higher weight is the best way to facilitate behavior change.
   1. From where do you think this myth originated?
   2. How can this myth harm patients in the context of clinical care? How is it relevant in this case?

**Part 5 (15 mins)**

After obtaining the history and physical and establishing rapport with the patient, you refer her for psychosocial support with medical case management. You note the important steps she has taken to improve her health, and plan for close follow-up in one month. At that time, you agree to discuss in more detail management of her hypertension, hyperlipidemia, and unintentional weight gain. You also discuss with her the option of changing her antiretroviral medications, but she is not ready to make a change at this time because her current regimen is convenient and has worked well for her.

One month later, you are preparing to see the patient for follow-up.

**Prompting questions:**

1. Putting everything together, what recommendations do you have for the patient?
2. How might an approach based on body diversity and acceptance facilitate your discussions with the patient surrounding her medical issues and her concerns about her weight? Do you have concerns about this approach? What elements of this approach do you view as most valuable to patient care?

**Case resolution and wrap-up (15-20 mins)**

Over the next several visits, you recommend sleep hygiene, reducing salt, carbohydrate and sugar intake, and regular physical activity over the next 6 months. You suggest changing her HIV medication and starting a new antihypertensive. She is ready to change her HIV medication but reluctant to restart an antihypertensive in light of her experience prior. She would like to try to manage her blood pressure with lifestyle, which is also more consistent with her desire to use natural remedies rather than medications. She starts rosuvastatin for hyperlipidemia.

You refer the patient for mental health services. She does not want to start an anti-depressant medication since she is worried about the side effects. With encouragement and support from her medical case manager, she successfully discloses her HIV status to her sister and her older daughter, and they are very supportive, which helps her with the burden of HIV stigma. Her relationship with her partner ends on mutual terms and he moves out. She continues to take on extra work as a home health aide and at times her job remains quite stressful, but she has a few patients for whom she regularly cares and feels that she gets a sense of purpose from her work.

Over the next year, the patient meets regularly with a trained Health At Every Size® dietitian, and they work together to create meal plans within her budget that reduce salt, sugars and carbohydrates and increase potassium-rich foods, fresh fruits and vegetables, working to be more attuned to her eating habits and eating more things that she likes. She starts a regular exercise program 2-3 times a week by going to the local gym to swim, which she always had enjoyed. She reports feeling more energized, fit, and hopeful. At 18 months after her first visit, her vital sign and lab parameters are:

- BP 136/70
- Triglycerides 168
- LDL 145, HDL 53
- A1c 5.7
- ALT 21

**Prompting questions:**

1. What are some of the factors in the case that led to improved clinical outcomes in her blood pressure and labs?
2. What are the top three take-home messages that you learned from the pre-assignments and the case discussion?

*Contributors to this guide: Dr. Michelle DallaPiazza, Dr. Joy Cox, Dr. Dhvani Doshi, Dr. Victor Cueto, Dr. Aleksey Tentler. Rutgers New Jersey Medical School*
