# Supplementary material for: An Educational Session for Medical Students Exploring Weight Bias in Clinical Care Through the Lens of Body Diversity
Source: MedEdPORTAL. 2023 Sep 5;19:11342. doi: 10.15766/mep_2374-8265.11342 (PMC10477274; doi:10.15766/mep_2374-8265.11342)
Supplement: Supplementary file 1 — Understanding Body Diversity.pptxAddressing Weight Bias in Clinical Care.pptxFacilitator Guide.docxStudent Guide.docxMaterials Checklist and Timeline.docxQuiz.docxEvaluation Survey.docx [file mep_2374-8265.11342-s001.zip › F. Quiz.docx]

**Appendix F - Quiz**

**Weight Bias**

***Instructions:*** *This quiz can be offered electronically as a part of the educational management system. Submission of the quiz can be due one week after the session. Students can use course and outside resources to answer the questions (open content) and have one untimed attempt.*

Question 1 (Objective 1)

**1 point**

Which of the following critiques of the weight-based health paradigm argues against the tenet that body weight is volitional and within the control of the individual?

1. **A powerful biologic system regulates metabolic rate and an unconscious drive to eat and move**
2. A focus on weight instead of health and social determinants can enhance weight stigma
3. Conventional weight loss diets rarely lead to sustained weight loss over the long run, and can lead to weight cycling
4. Creating a less obesogenic environment will reduce the prevalence of obesity

Question 2 (Objective 2)

**5 points**

Of the many factors that influence weight within the 4 main categories, which did you find to be most interesting or surprising and why?

Question 3 (Objective 3)

**1 point**

According to a study by Amy et al in 2006, cisgender women who weigh more report that their weight is a barrier to their healthcare because of which of the following sets of experiences in healthcare settings?

1. Lack of access to weight loss surgery
2. **Unsolicited advice to lose weight**
3. A focus on medical problems unrelated to obesity
4. Embarrassment at giving a medical history

Question 4 (Objective 4)

**1 point**

Health at every size (HAES) is a paradigm that emphasizes:

1. Providers' critical self-reflection to examine and address internalized weight bias
2. Patient-centered language in the medical record
3. **Holistic health and well-being for all body shapes and sizes**
4. Six core areas of lifestyle medicine to achieve better health outcomes

Question 5 (Objective 5)

**5 points**

Reflecting on body acceptance and body diversity, how can incorporation of these concepts into the care of patients influence their healthcare experiences?

Question 6 (Multiple objectives)

**5 points**

During the case discussion, which obesity myth discussion did you find to be most interesting and why?

**Total points: 18**

**Grading Rubric for essay questions**

Please take note of the proportion of the quiz final score that the question represents and
ensure the effort used to generate the answer reflects this value. Students may receive either full,
partial, or no credit for their answer.
 Full credit
 Partial credit – score will reflect less than the full value of the question’s worth
 No credit (zero score)

Full credit: Answer demonstrates thoughtful reflection, engagement with the course material and
activities, and understanding of the topic

Partial credit: While complete, the answer fails to demonstrate thoughtfulness, meaningful
engagement with the materials, and/or full understanding of the topic

No credit: The answer was incomplete, showed no evidence of meaningful reflection or
thoughtfulness, or did not address the topic of the question

Common pitfalls that will result in partial credit:

1) The answer does not reflect the effort needed to fulfill full credit based on the weight of the
question’s worth. Many short answer questions are worth 5 points, while multiple choice
questions would be worth 1 point each. It is important to demonstrate the thoughtfulness and
effort that will warrant such a high weight for one question. For example, it is difficult to
demonstrate that you spent time and effort worth 5 points in just 2-3 sentences.

2) While the length of the response is adequate, within the content of the response the ideas are
repetitive, do not demonstrate any critical analysis of the course content, do not demonstrate
self-reflection, or seem for the large part to copy the stem of the questions itself without
adding any significantly new information.

3) The response seems generic or reflective of broad themes and does not cite examples or
reflect effort in engaging with the course materials/small group sessions.
