# Supplementary material for: An Educational Session for Medical Students Exploring Weight Bias in Clinical Care Through the Lens of Body Diversity
Source: MedEdPORTAL. 2023 Sep 5;19:11342. doi: 10.15766/mep_2374-8265.11342 (PMC10477274; doi:10.15766/mep_2374-8265.11342)
Supplement: Supplementary file 1 — Understanding Body Diversity.pptxAddressing Weight Bias in Clinical Care.pptxFacilitator Guide.docxStudent Guide.docxMaterials Checklist and Timeline.docxQuiz.docxEvaluation Survey.docx [file mep_2374-8265.11342-s001.zip › G. Evaluation Survey.docx]

**Appendix G - Evaluation Survey**

**Weight Bias**

Using the scale below, please evaluate this activity in terms of:

|  | Poor | Only fair | Adequate | Good | Excellent |
| --- | --- | --- | --- | --- | --- |
| Overall content |  |  |  |  |  |
| Lectures |  |  |  |  |  |
| Small Group |  |  |  |  |  |
| Small Group Facilitator |  |  |  |  |  |

My knowledge and/or skills improved after this activity with respect to my ability to:

|  | Hardly at all | To a small degree | To a moderate degree | To a considerable degree | To a very high degree |
| --- | --- | --- | --- | --- | --- |
| Discuss critiques of the weight-centered health paradigm |  |  |  |  |  |
| Describe the multifactorial influences on body weight |  |  |  |  |  |
| Describe the effect that weight bias can have on healthcare access, delivery, and outcomes |  |  |  |  |  |
| Outline approaches to reduce weight stigma in the healthcare setting |  |  |  |  |  |
| Discuss body diversity and body acceptance and their relevance to clinical care |  |  |  |  |  |

Rate the degree to which you agree with the following statements:

|  | Hardly at all | To a small degree | To a moderate degree | To a considerable degree | To a very high degree |
| --- | --- | --- | --- | --- | --- |
| Additional training on this topic will be beneficial for learning to be an effective physician. |  |  |  |  |  |
| I have a greater appreciation after this session for the value of body acceptance in clinical care. |  |  |  |  |  |

Please comment on the strengths of this activity.

Please provide suggestions on how this activity can be improved.

Thank you for your feedback!
