# Supplementary material for: An Educational Session for Medical Students Exploring Weight Bias in Clinical Care Through the Lens of Body Diversity
Source: MedEdPORTAL. 2023 Sep 5;19:11342. doi: 10.15766/mep_2374-8265.11342 (PMC10477274; doi:10.15766/mep_2374-8265.11342)
Supplement: Supplementary file 1 — Understanding Body Diversity.pptxAddressing Weight Bias in Clinical Care.pptxFacilitator Guide.docxStudent Guide.docxMaterials Checklist and Timeline.docxQuiz.docxEvaluation Survey.docx [file mep_2374-8265.11342-s001.zip › A. Understanding Body Diversity.pptx]

## Slide 1
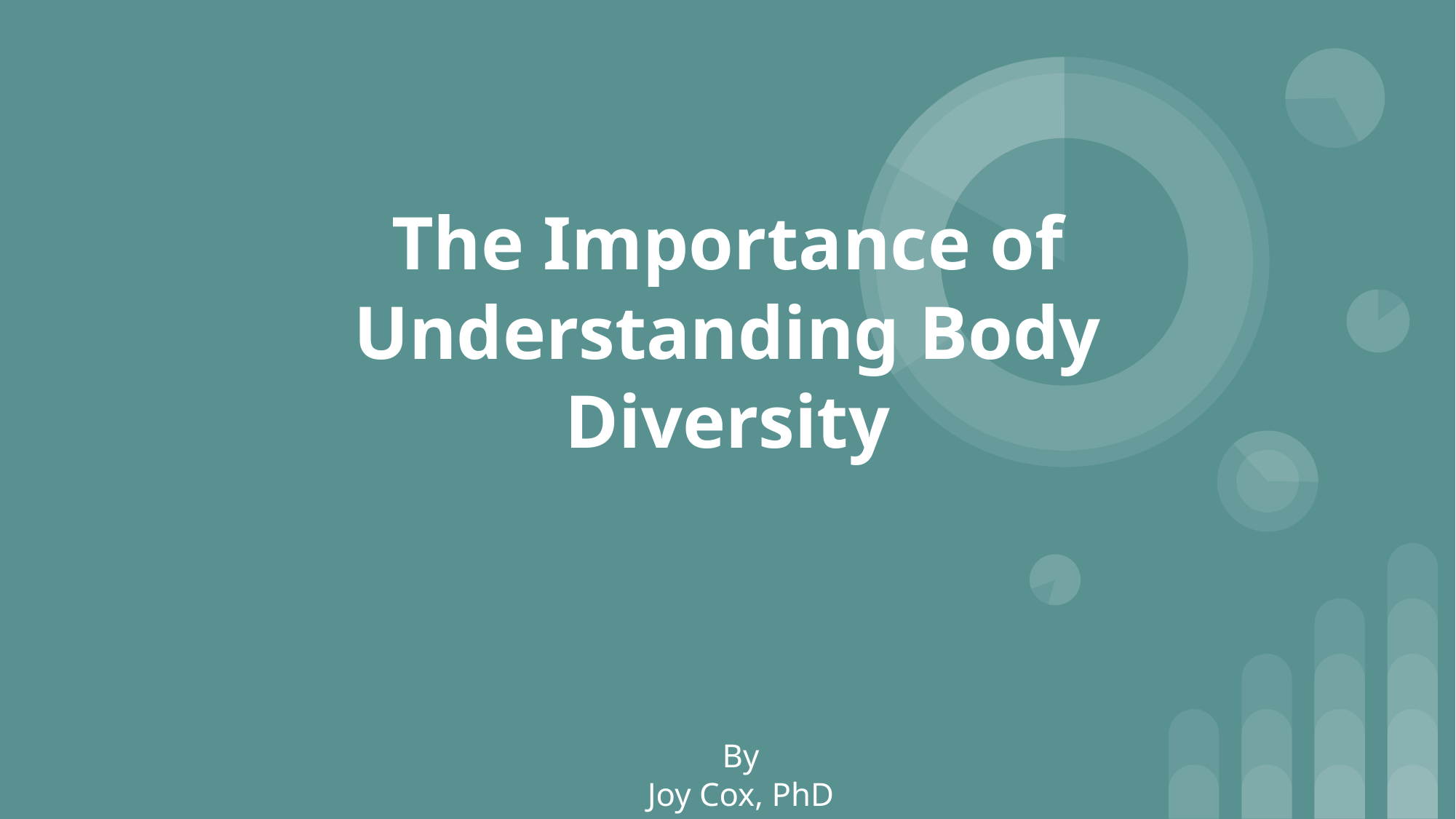

# The Importance of Understanding Body Diversity
By
Joy Cox, PhD

## Slide 2
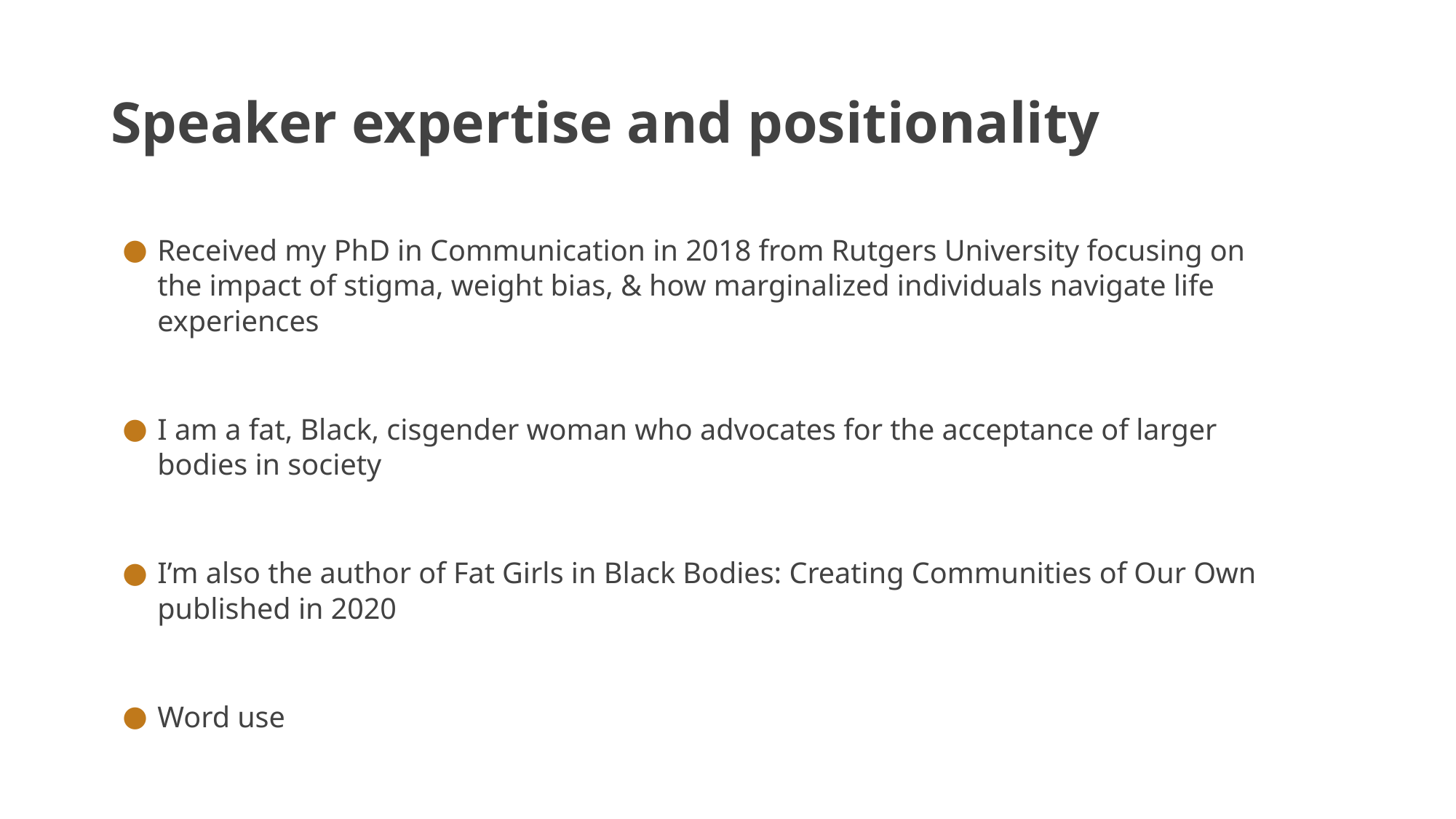

# Speaker expertise and positionality
Received my PhD in Communication in 2018 from Rutgers University focusing on the impact of stigma, weight bias, & how marginalized individuals navigate life experiences
I am a fat, Black, cisgender woman who advocates for the acceptance of larger bodies in society
I’m also the author of Fat Girls in Black Bodies: Creating Communities of Our Own published in 2020
Word use

## Slide 3
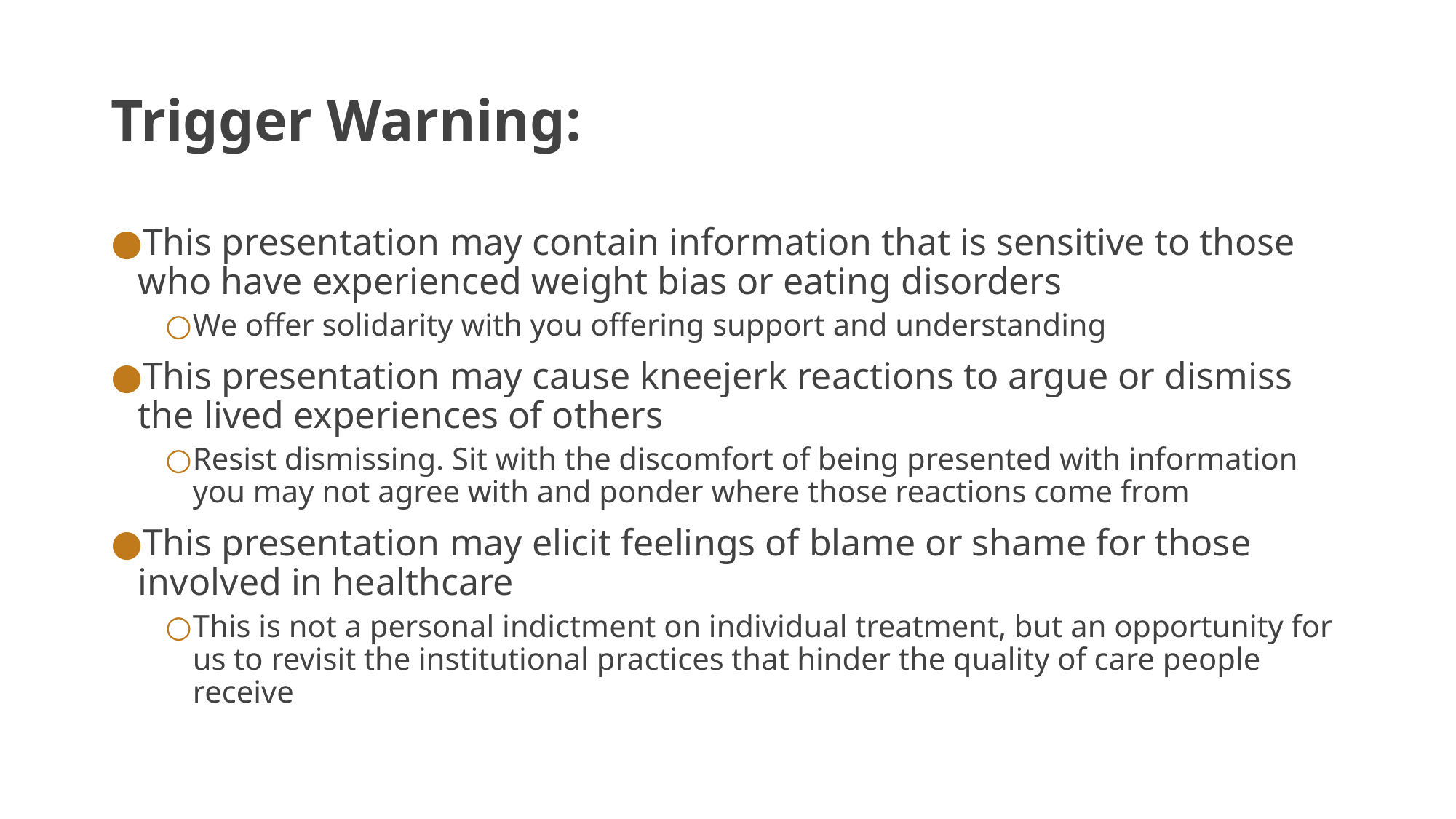

# Trigger Warning:
This presentation may contain information that is sensitive to those who have experienced weight bias or eating disorders
We offer solidarity with you offering support and understanding
This presentation may cause kneejerk reactions to argue or dismiss the lived experiences of others
Resist dismissing. Sit with the discomfort of being presented with information you may not agree with and ponder where those reactions come from
This presentation may elicit feelings of blame or shame for those involved in healthcare
This is not a personal indictment on individual treatment, but an opportunity for us to revisit the institutional practices that hinder the quality of care people receive

## Slide 4
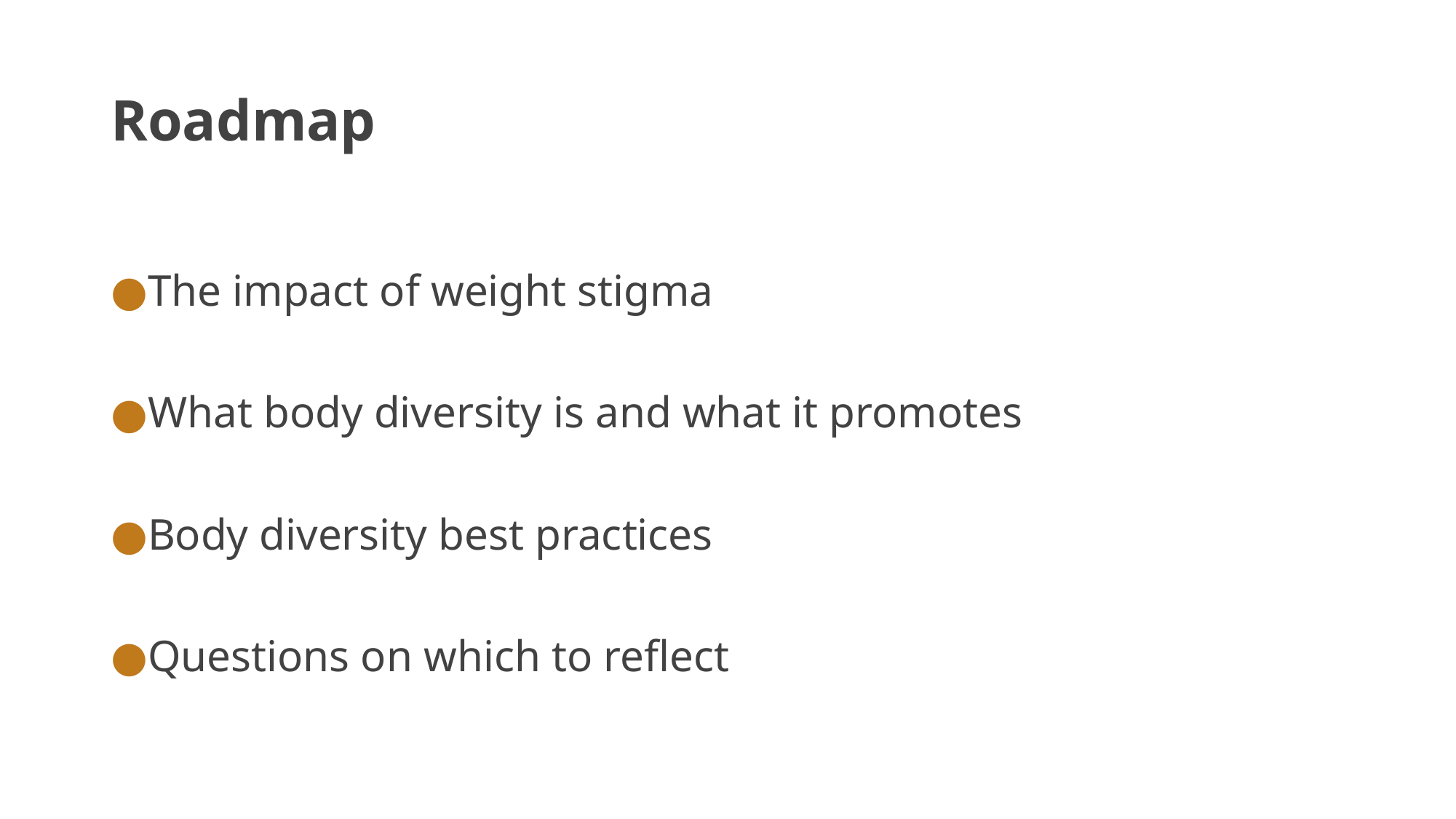

# Roadmap
The impact of weight stigma
What body diversity is and what it promotes
Body diversity best practices
Questions on which to reflect

## Slide 5
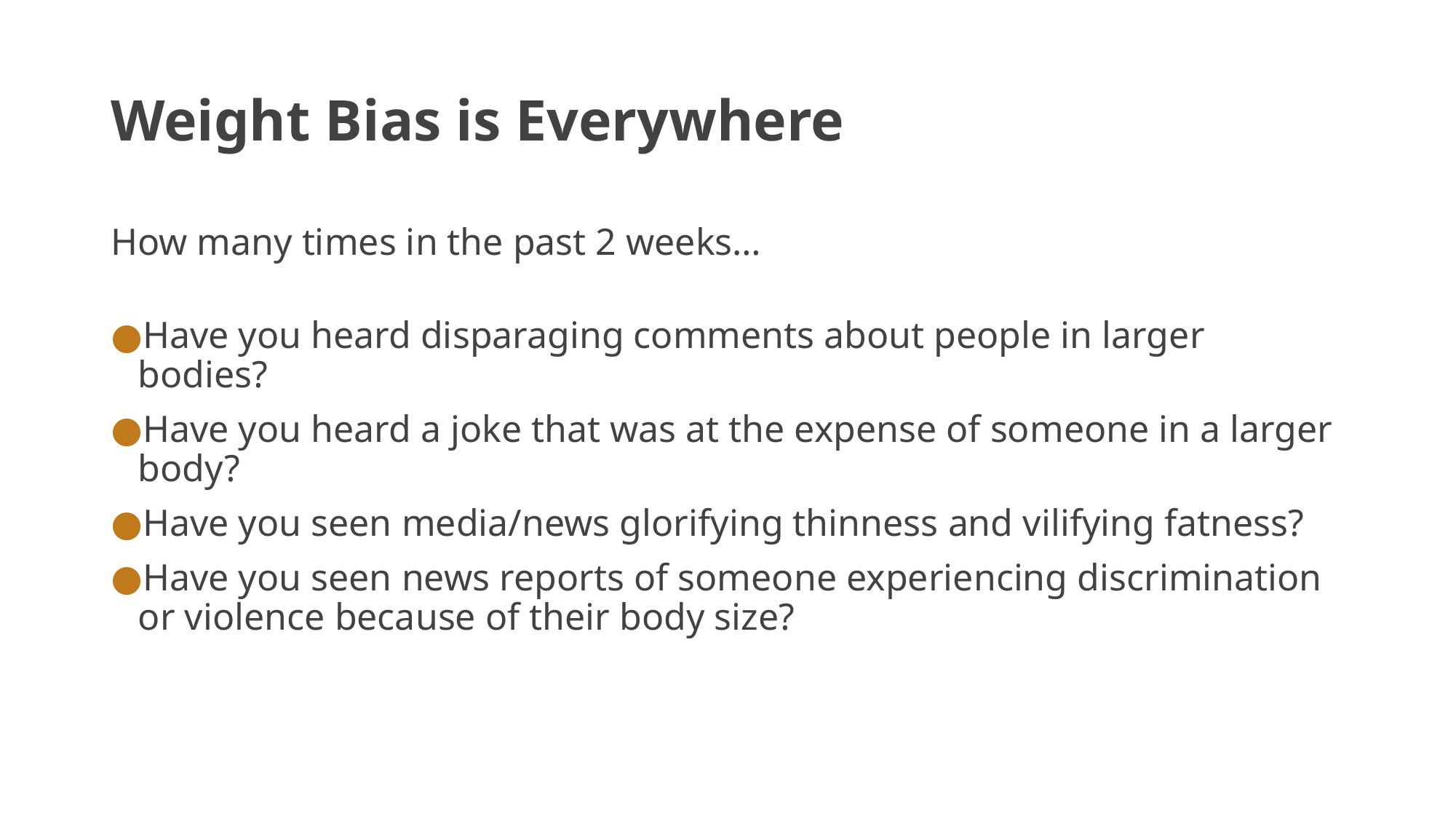

# Weight Bias is Everywhere
How many times in the past 2 weeks…
Have you heard disparaging comments about people in larger bodies?
Have you heard a joke that was at the expense of someone in a larger body?
Have you seen media/news glorifying thinness and vilifying fatness?
Have you seen news reports of someone experiencing discrimination or violence because of their body size?

## Slide 6
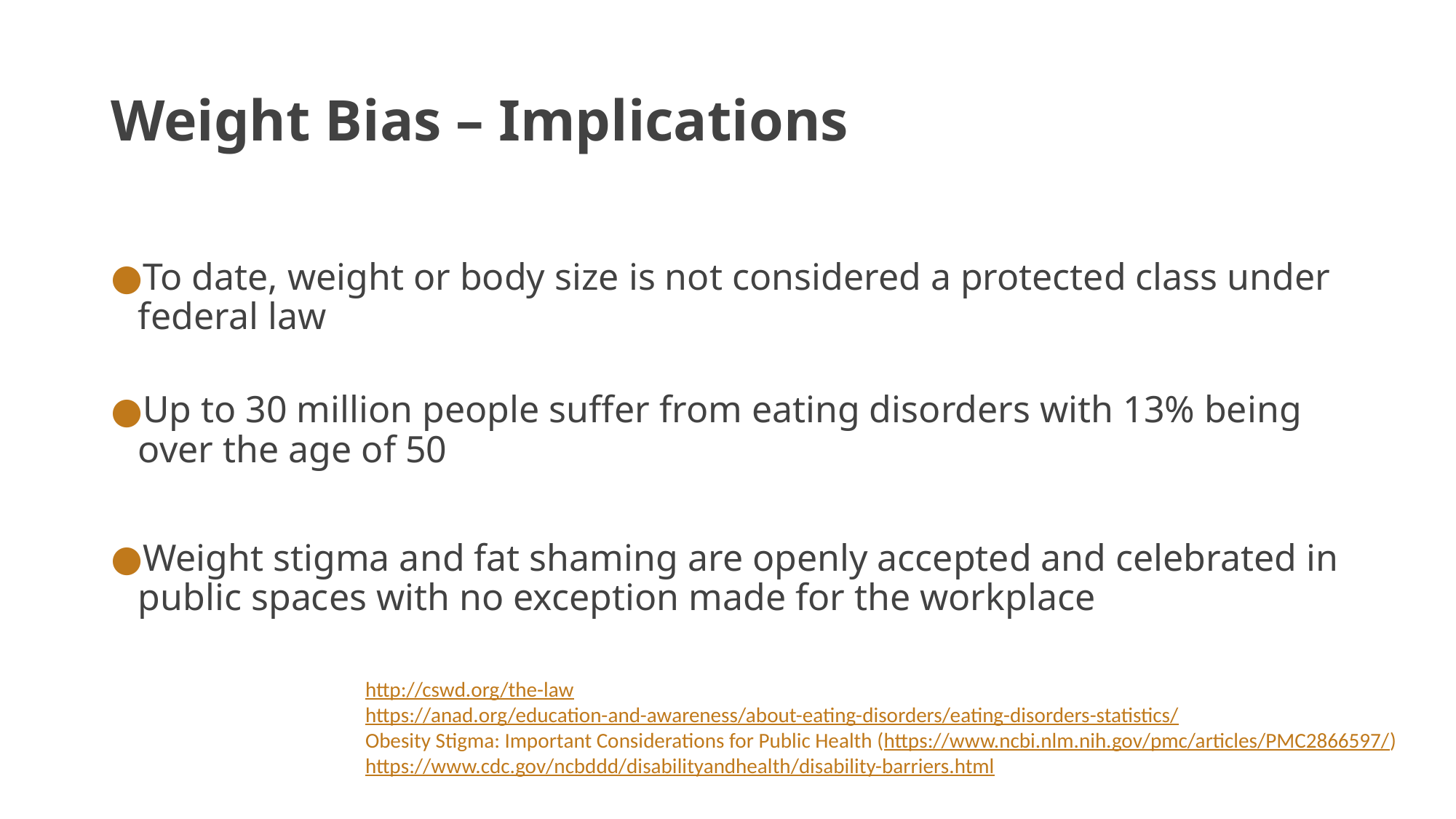

# Weight Bias – Implications
To date, weight or body size is not considered a protected class under federal law
Up to 30 million people suffer from eating disorders with 13% being over the age of 50
Weight stigma and fat shaming are openly accepted and celebrated in public spaces with no exception made for the workplace
http://cswd.org/the-law
https://anad.org/education-and-awareness/about-eating-disorders/eating-disorders-statistics/
Obesity Stigma: Important Considerations for Public Health (https://www.ncbi.nlm.nih.gov/pmc/articles/PMC2866597/)
https://www.cdc.gov/ncbddd/disabilityandhealth/disability-barriers.html

## Slide 7
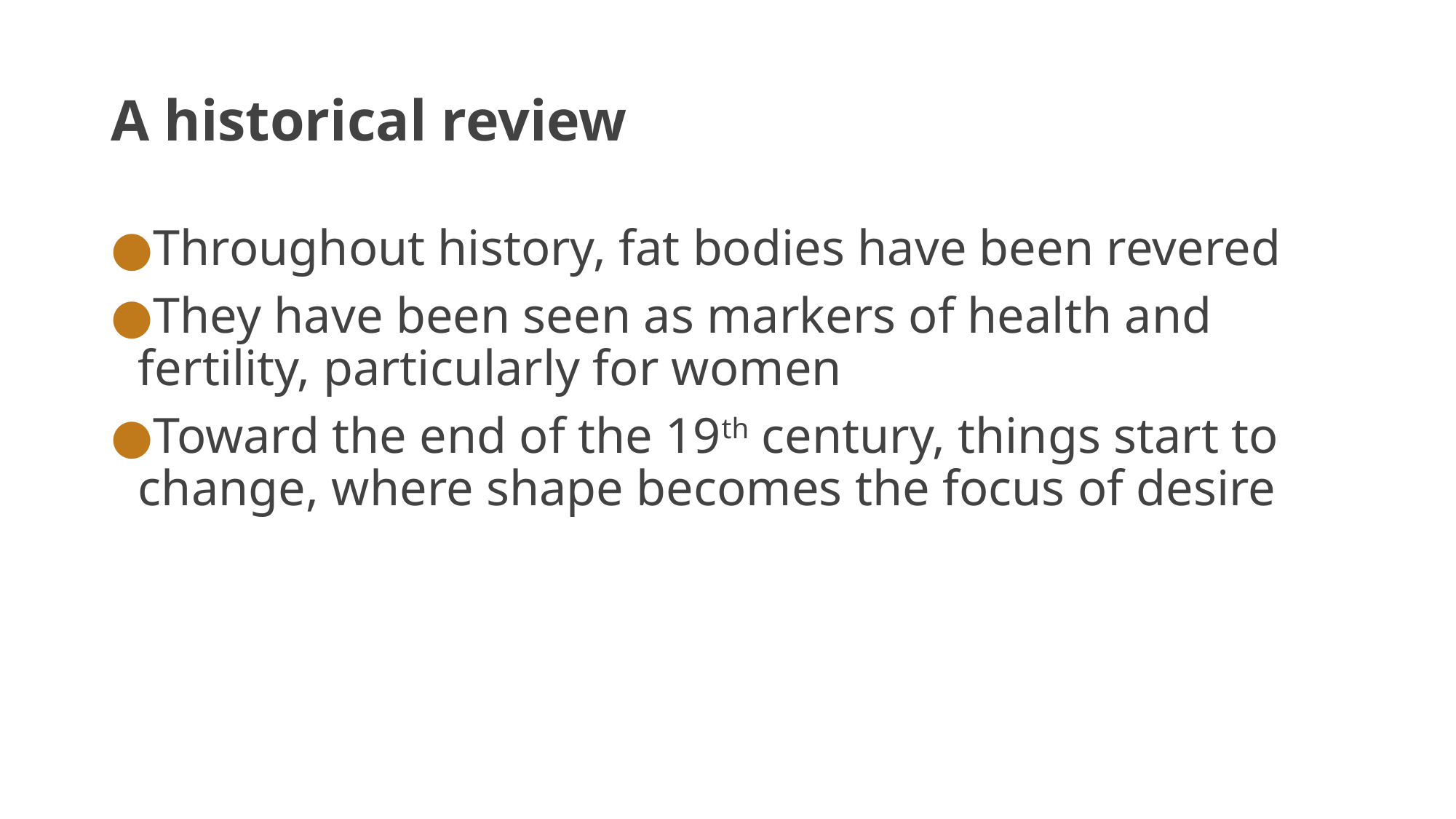

# A historical review
Throughout history, fat bodies have been revered
They have been seen as markers of health and fertility, particularly for women
Toward the end of the 19th century, things start to change, where shape becomes the focus of desire

## Slide 8
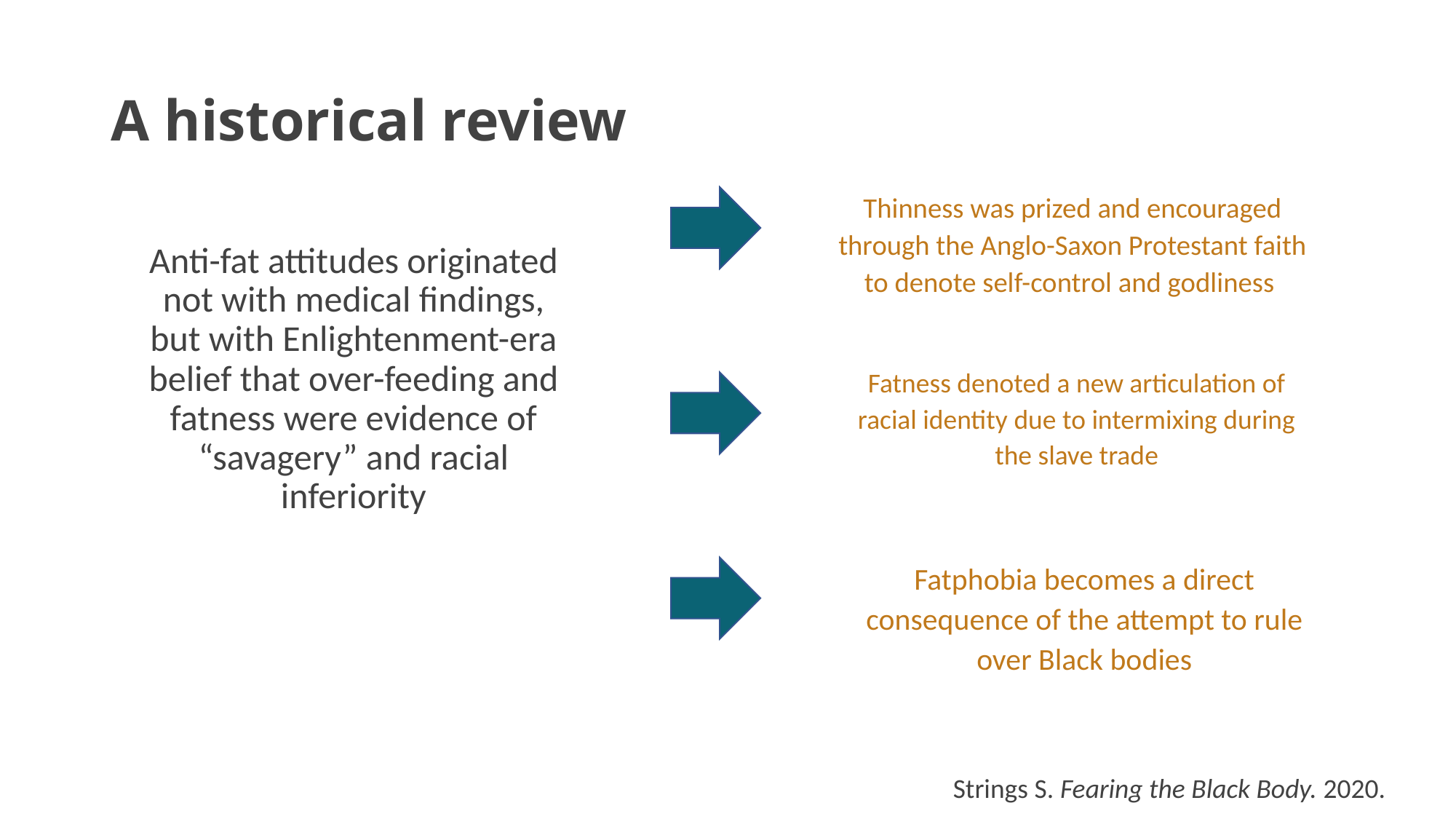

# A historical review
Thinness was prized and encouraged through the Anglo-Saxon Protestant faith to denote self-control and godliness
Anti-fat attitudes originated not with medical findings, but with Enlightenment-era belief that over-feeding and fatness were evidence of “savagery” and racial inferiority
Fatness denoted a new articulation of racial identity due to intermixing during the slave trade
Fatphobia becomes a direct consequence of the attempt to rule over Black bodies
Strings S. Fearing the Black Body. 2020.

## Slide 9
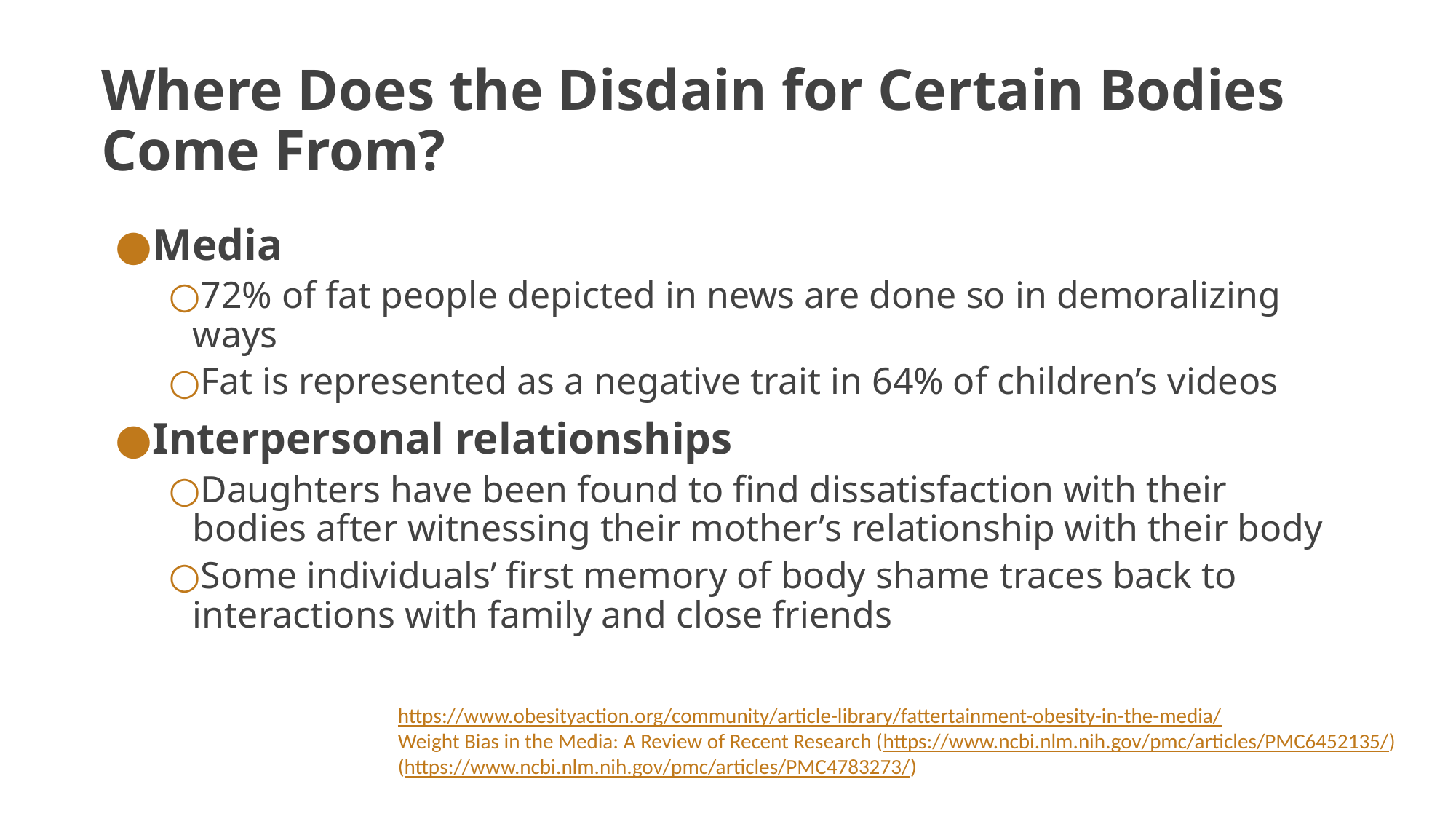

# Where Does the Disdain for Certain Bodies Come From?
Media
72% of fat people depicted in news are done so in demoralizing ways
Fat is represented as a negative trait in 64% of children’s videos
Interpersonal relationships
Daughters have been found to find dissatisfaction with their bodies after witnessing their mother’s relationship with their body
Some individuals’ first memory of body shame traces back to interactions with family and close friends
https://www.obesityaction.org/community/article-library/fattertainment-obesity-in-the-media/
Weight Bias in the Media: A Review of Recent Research (https://www.ncbi.nlm.nih.gov/pmc/articles/PMC6452135/)
(https://www.ncbi.nlm.nih.gov/pmc/articles/PMC4783273/)
https://blog.marketresearch.com/u.s.-weight-loss-industry-grows-to-72-billion
Beauty and Body Image Concerns Among African American College Women (https://www.ncbi.nlm.nih.gov/pmc/articles/PMC4713035/)

## Slide 10
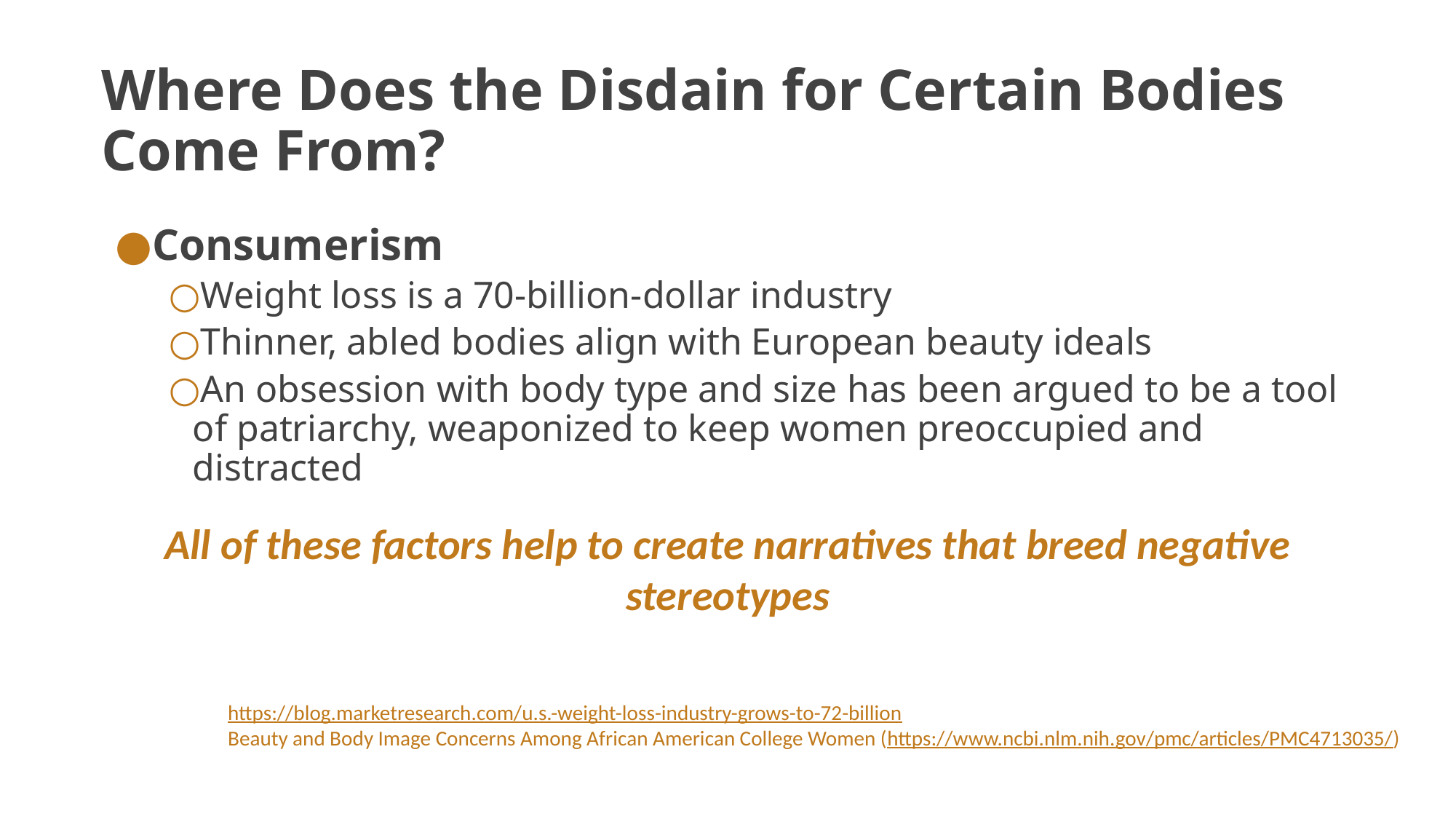

# Where Does the Disdain for Certain Bodies Come From?
Consumerism
Weight loss is a 70-billion-dollar industry
Thinner, abled bodies align with European beauty ideals
An obsession with body type and size has been argued to be a tool of patriarchy, weaponized to keep women preoccupied and distracted
All of these factors help to create narratives that breed negative stereotypes
https://blog.marketresearch.com/u.s.-weight-loss-industry-grows-to-72-billion
Beauty and Body Image Concerns Among African American College Women (https://www.ncbi.nlm.nih.gov/pmc/articles/PMC4713035/)
https://www.obesityaction.org/community/article-library/fattertainment-obesity-in-the-media/
Weight Bias in the Media: A Review of Recent Research (https://www.ncbi.nlm.nih.gov/pmc/articles/PMC6452135/)
In It Together: Mother Talk of Weight Concerns Moderates Negative Outcomes of Encouragement to Lose Weight on Daughter Body Dissatisfaction and Disordered Eating (https://www.ncbi.nlm.nih.gov/pmc/articles/PMC4783273/)

## Slide 11
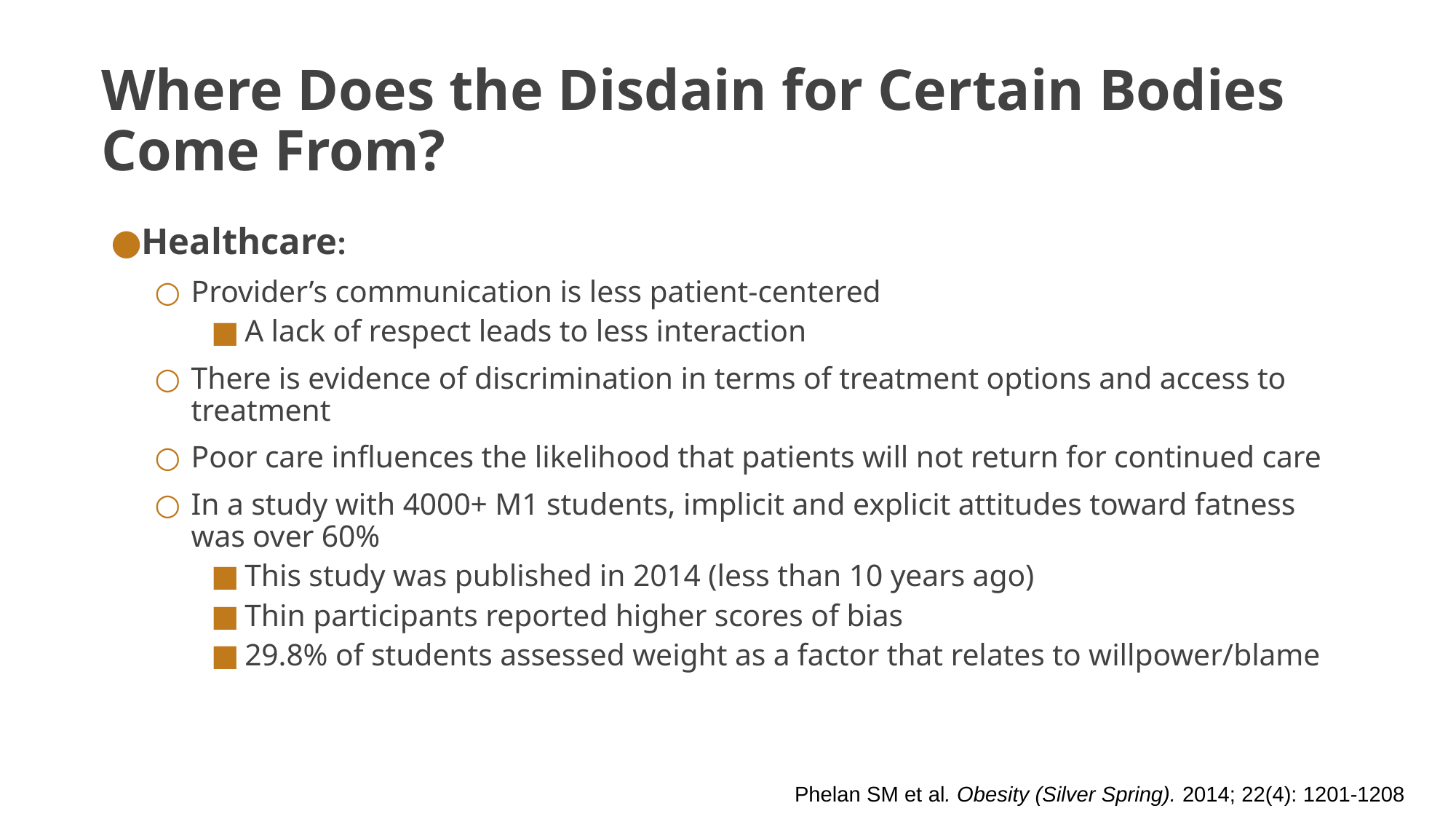

# Where Does the Disdain for Certain Bodies Come From?
Healthcare:
Provider’s communication is less patient-centered
A lack of respect leads to less interaction
There is evidence of discrimination in terms of treatment options and access to treatment
Poor care influences the likelihood that patients will not return for continued care
In a study with 4000+ M1 students, implicit and explicit attitudes toward fatness was over 60%
This study was published in 2014 (less than 10 years ago)
Thin participants reported higher scores of bias
29.8% of students assessed weight as a factor that relates to willpower/blame
Phelan SM et al. Obesity (Silver Spring). 2014; 22(4): 1201-1208

## Slide 12
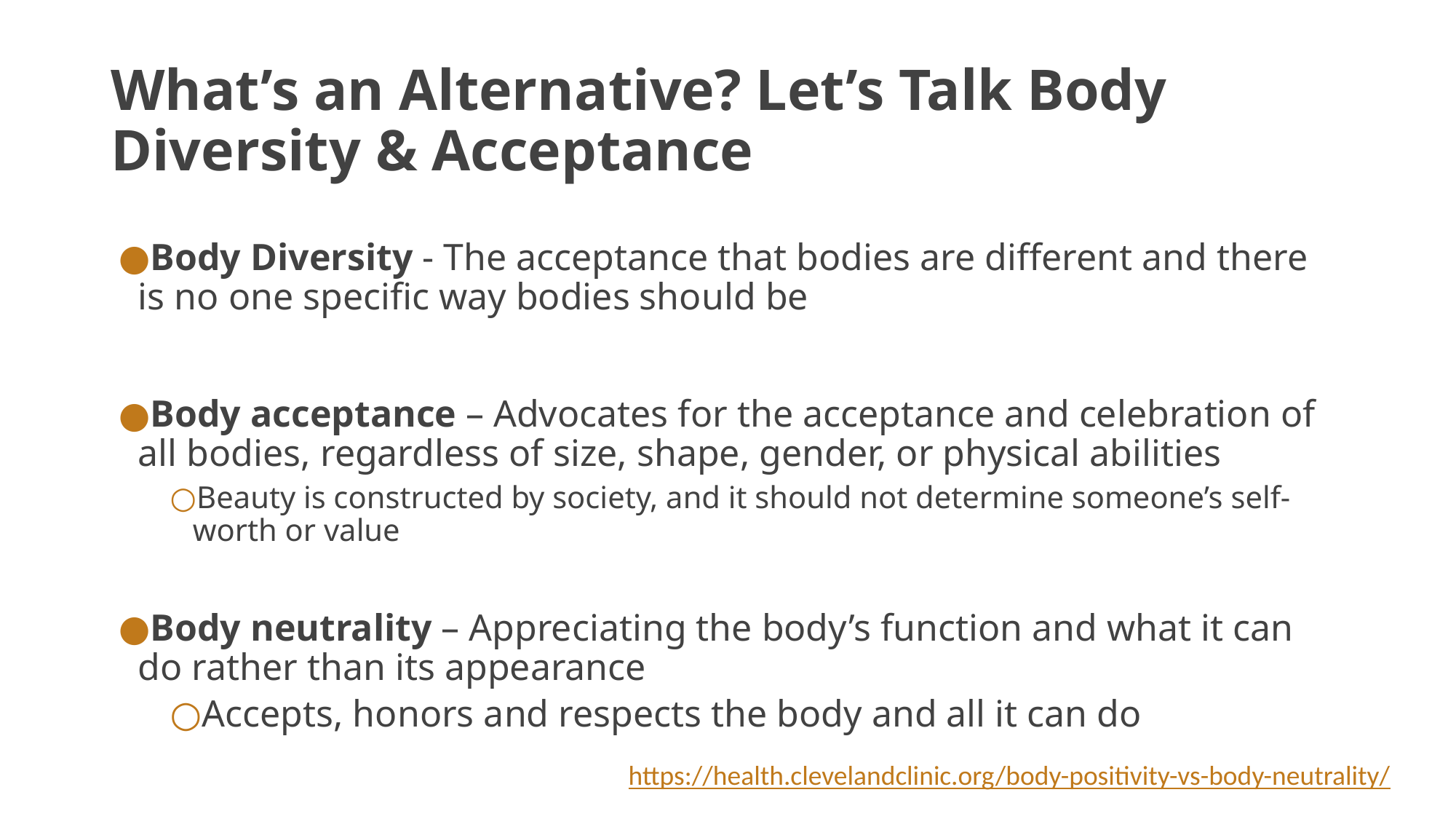

# What’s an Alternative? Let’s Talk Body Diversity & Acceptance
Body Diversity - The acceptance that bodies are different and there is no one specific way bodies should be
Body acceptance – Advocates for the acceptance and celebration of all bodies, regardless of size, shape, gender, or physical abilities
Beauty is constructed by society, and it should not determine someone’s self-worth or value
Body neutrality – Appreciating the body’s function and what it can do rather than its appearance
Accepts, honors and respects the body and all it can do
https://health.clevelandclinic.org/body-positivity-vs-body-neutrality/

## Slide 13
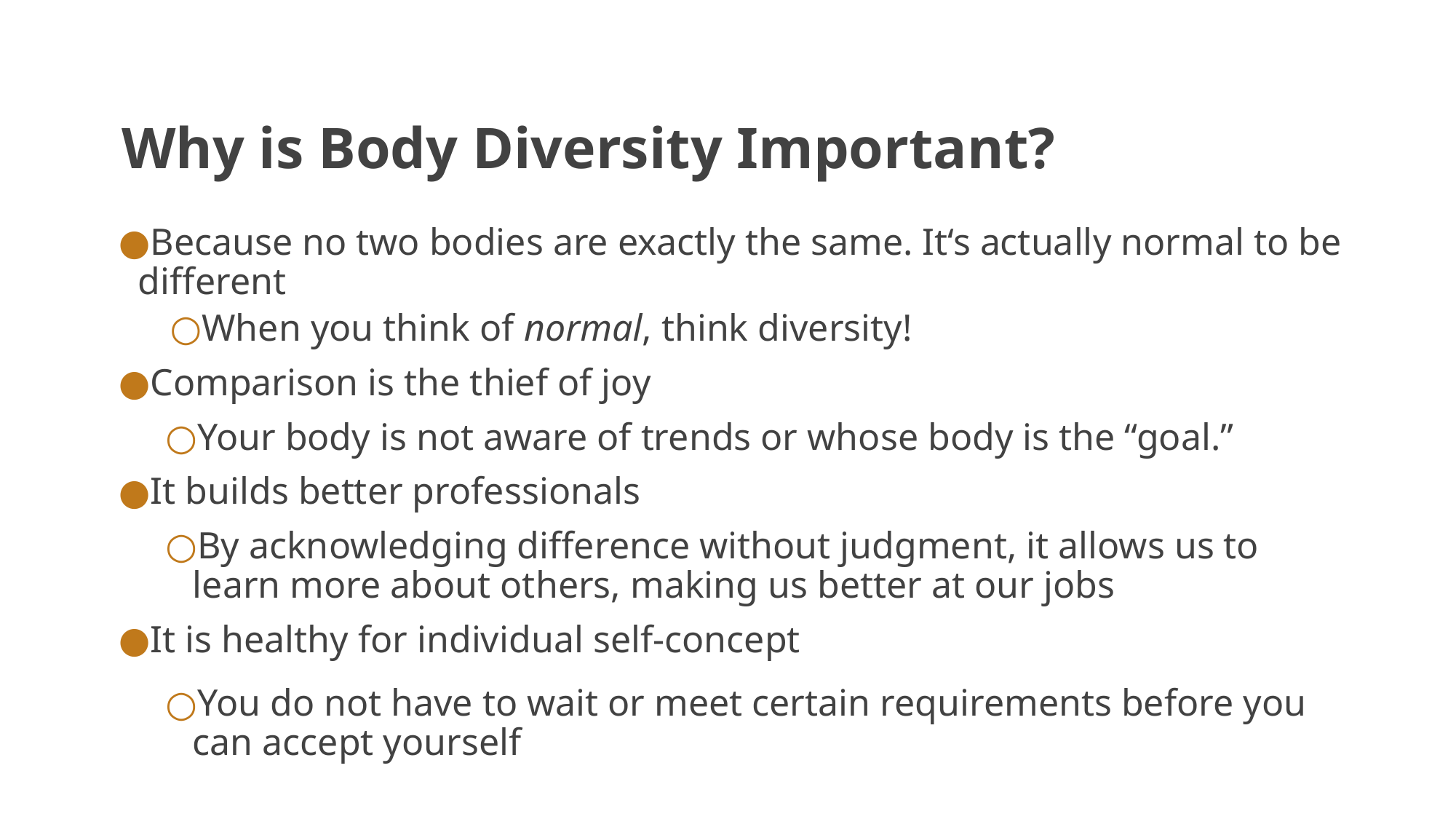

# Why is Body Diversity Important?
Because no two bodies are exactly the same. It‘s actually normal to be different
When you think of normal, think diversity!
Comparison is the thief of joy
Your body is not aware of trends or whose body is the “goal.”
It builds better professionals
By acknowledging difference without judgment, it allows us to learn more about others, making us better at our jobs
It is healthy for individual self-concept
You do not have to wait or meet certain requirements before you can accept yourself

## Slide 14
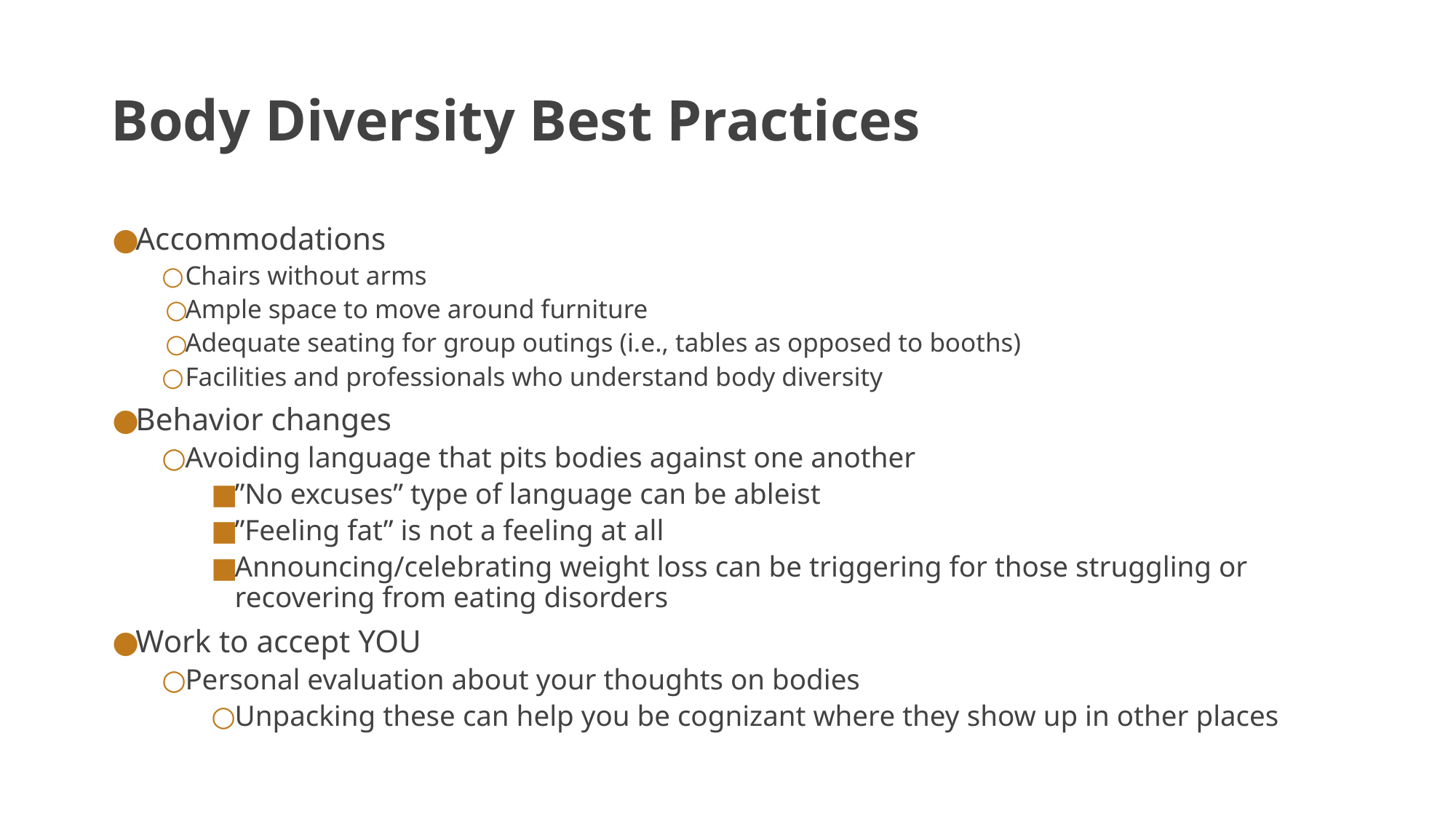

# Body Diversity Best Practices
Accommodations
Chairs without arms
Ample space to move around furniture
Adequate seating for group outings (i.e., tables as opposed to booths)
Facilities and professionals who understand body diversity
Behavior changes
Avoiding language that pits bodies against one another
”No excuses” type of language can be ableist
”Feeling fat” is not a feeling at all
Announcing/celebrating weight loss can be triggering for those struggling or recovering from eating disorders
Work to accept YOU
Personal evaluation about your thoughts on bodies
Unpacking these can help you be cognizant where they show up in other places

## Slide 15
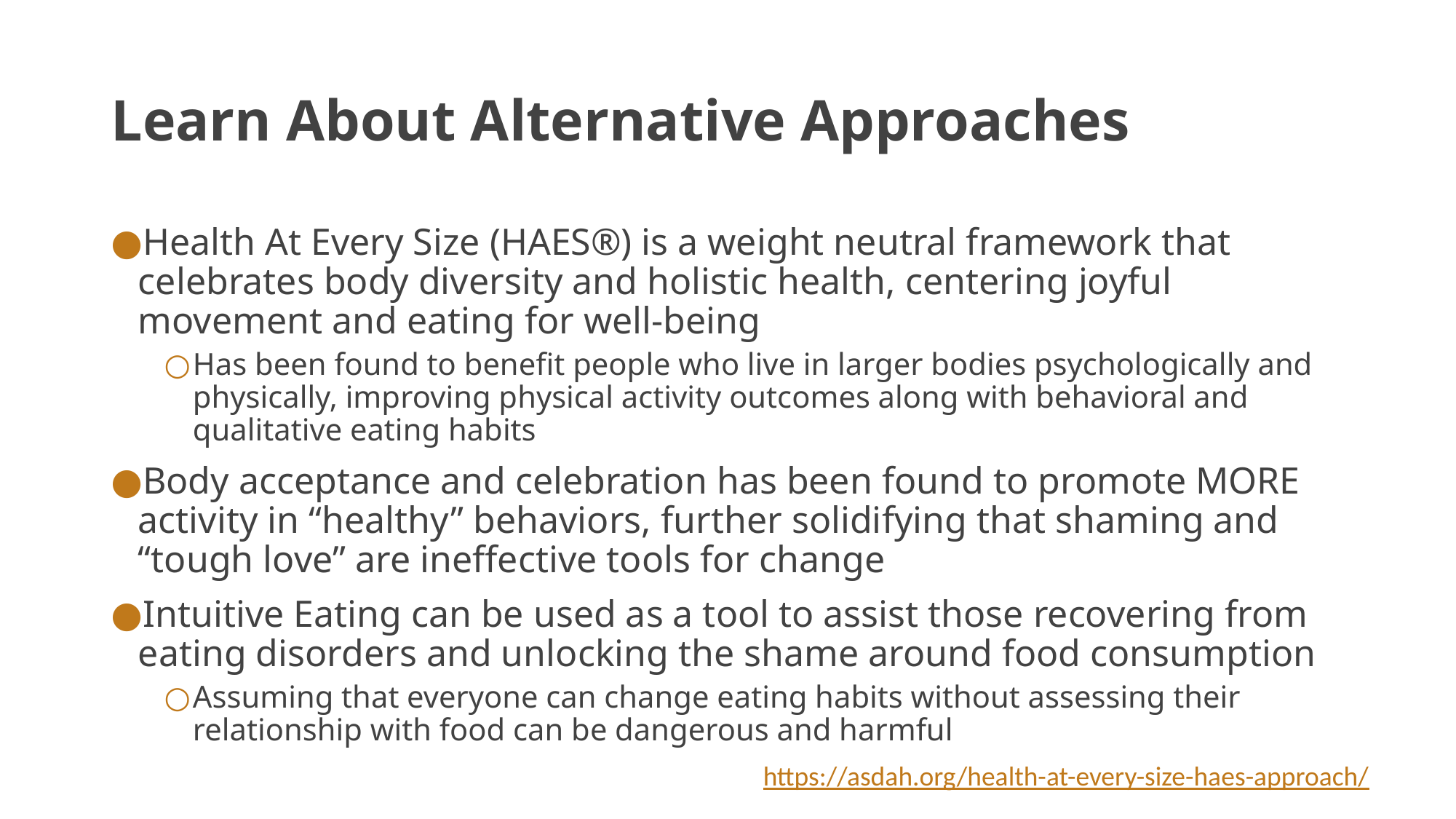

# Learn About Alternative Approaches
Health At Every Size (HAES®) is a weight neutral framework that celebrates body diversity and holistic health, centering joyful movement and eating for well-being
Has been found to benefit people who live in larger bodies psychologically and physically, improving physical activity outcomes along with behavioral and qualitative eating habits
Body acceptance and celebration has been found to promote MORE activity in “healthy” behaviors, further solidifying that shaming and “tough love” are ineffective tools for change
Intuitive Eating can be used as a tool to assist those recovering from eating disorders and unlocking the shame around food consumption
Assuming that everyone can change eating habits without assessing their relationship with food can be dangerous and harmful
https://asdah.org/health-at-every-size-haes-approach/

## Slide 16
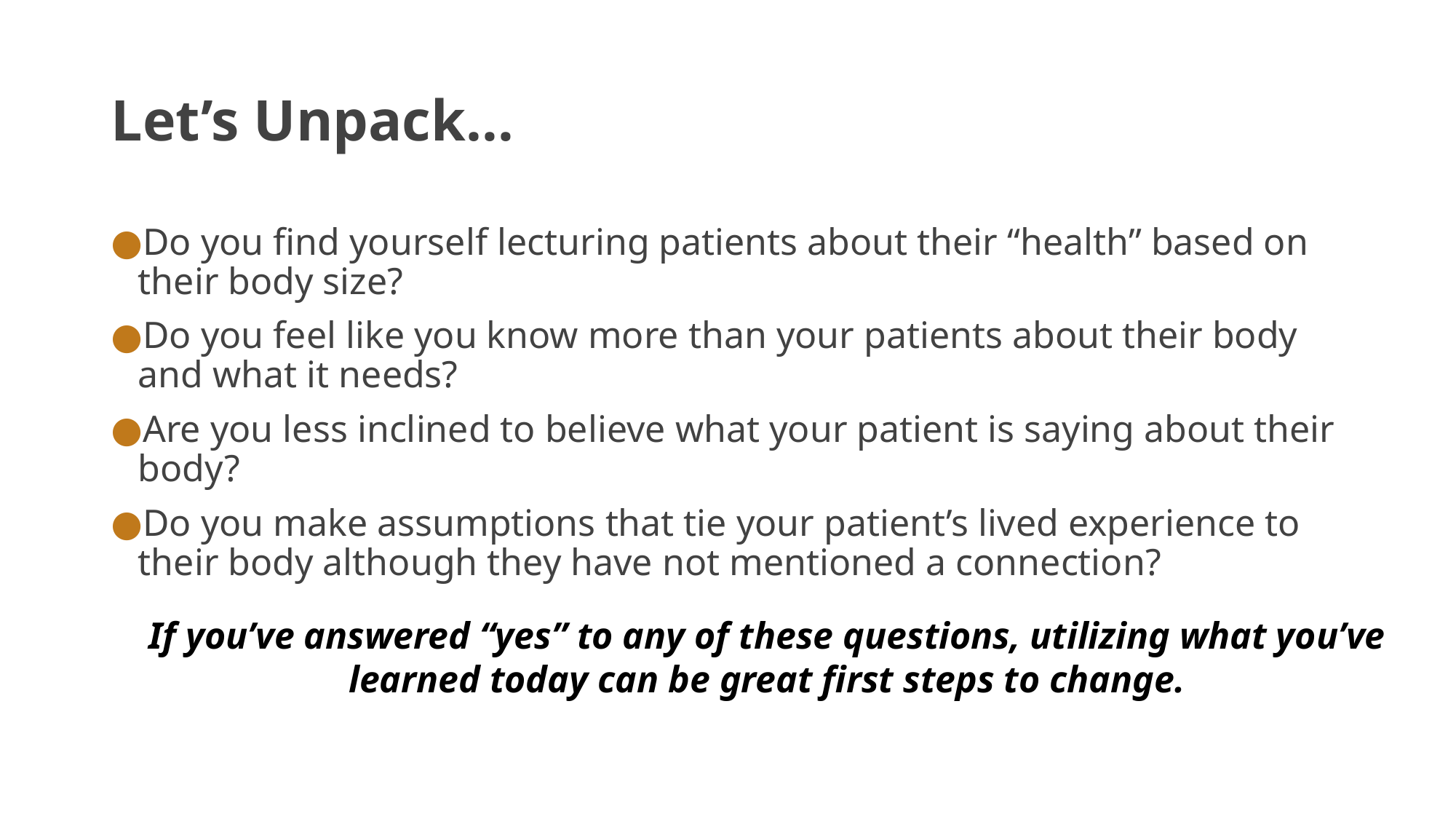

# Let’s Unpack…
Do you find yourself lecturing patients about their “health” based on their body size?
Do you feel like you know more than your patients about their body and what it needs?
Are you less inclined to believe what your patient is saying about their body?
Do you make assumptions that tie your patient’s lived experience to their body although they have not mentioned a connection?
If you’ve answered “yes” to any of these questions, utilizing what you’ve learned today can be great first steps to change.

## Slide 17
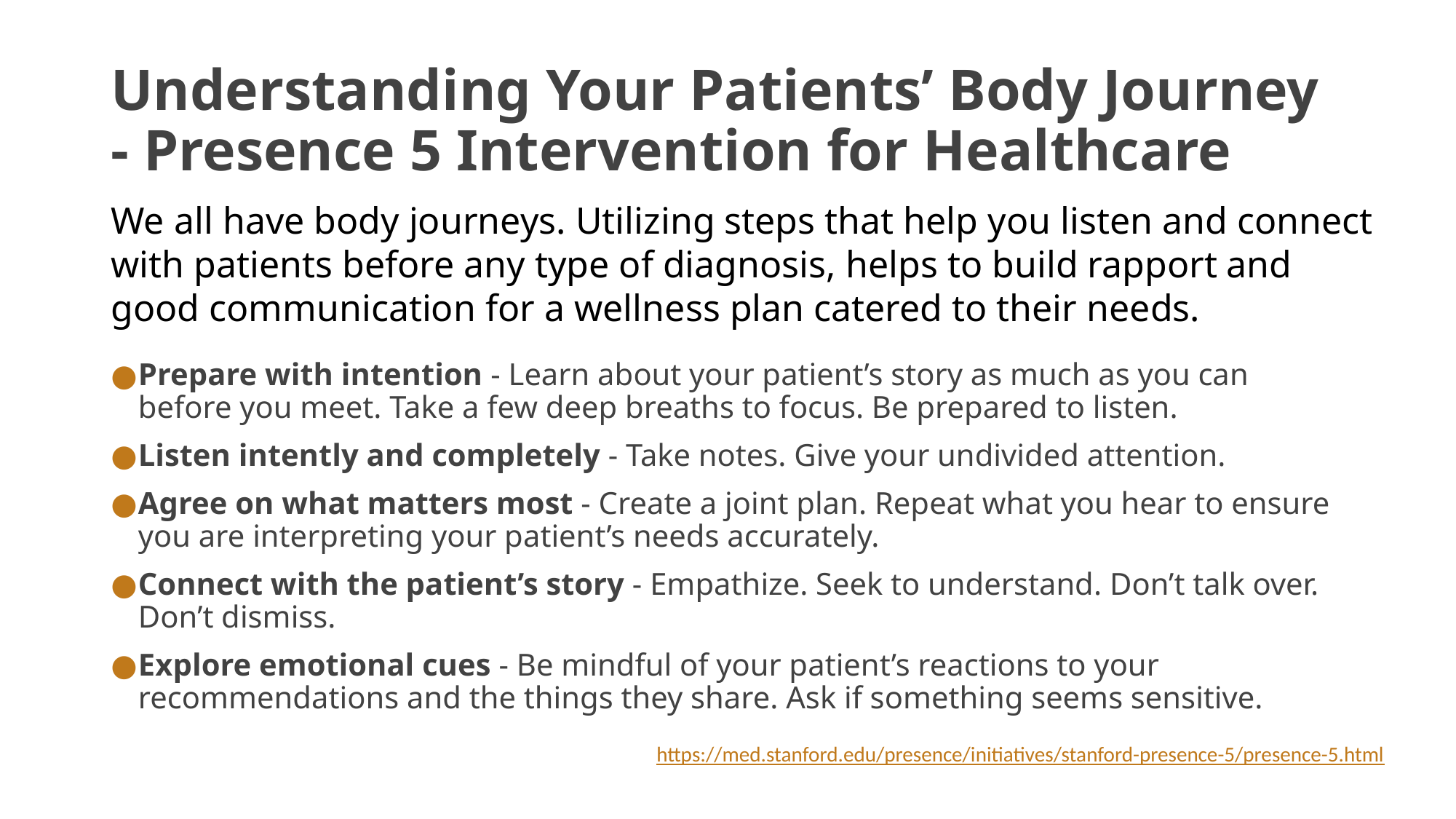

# Understanding Your Patients’ Body Journey - Presence 5 Intervention for Healthcare
We all have body journeys. Utilizing steps that help you listen and connect with patients before any type of diagnosis, helps to build rapport and good communication for a wellness plan catered to their needs.
Prepare with intention - Learn about your patient’s story as much as you can before you meet. Take a few deep breaths to focus. Be prepared to listen.
Listen intently and completely - Take notes. Give your undivided attention.
Agree on what matters most - Create a joint plan. Repeat what you hear to ensure you are interpreting your patient’s needs accurately.
Connect with the patient’s story - Empathize. Seek to understand. Don’t talk over. Don’t dismiss.
Explore emotional cues - Be mindful of your patient’s reactions to your recommendations and the things they share. Ask if something seems sensitive.
https://med.stanford.edu/presence/initiatives/stanford-presence-5/presence-5.html

## Slide 18
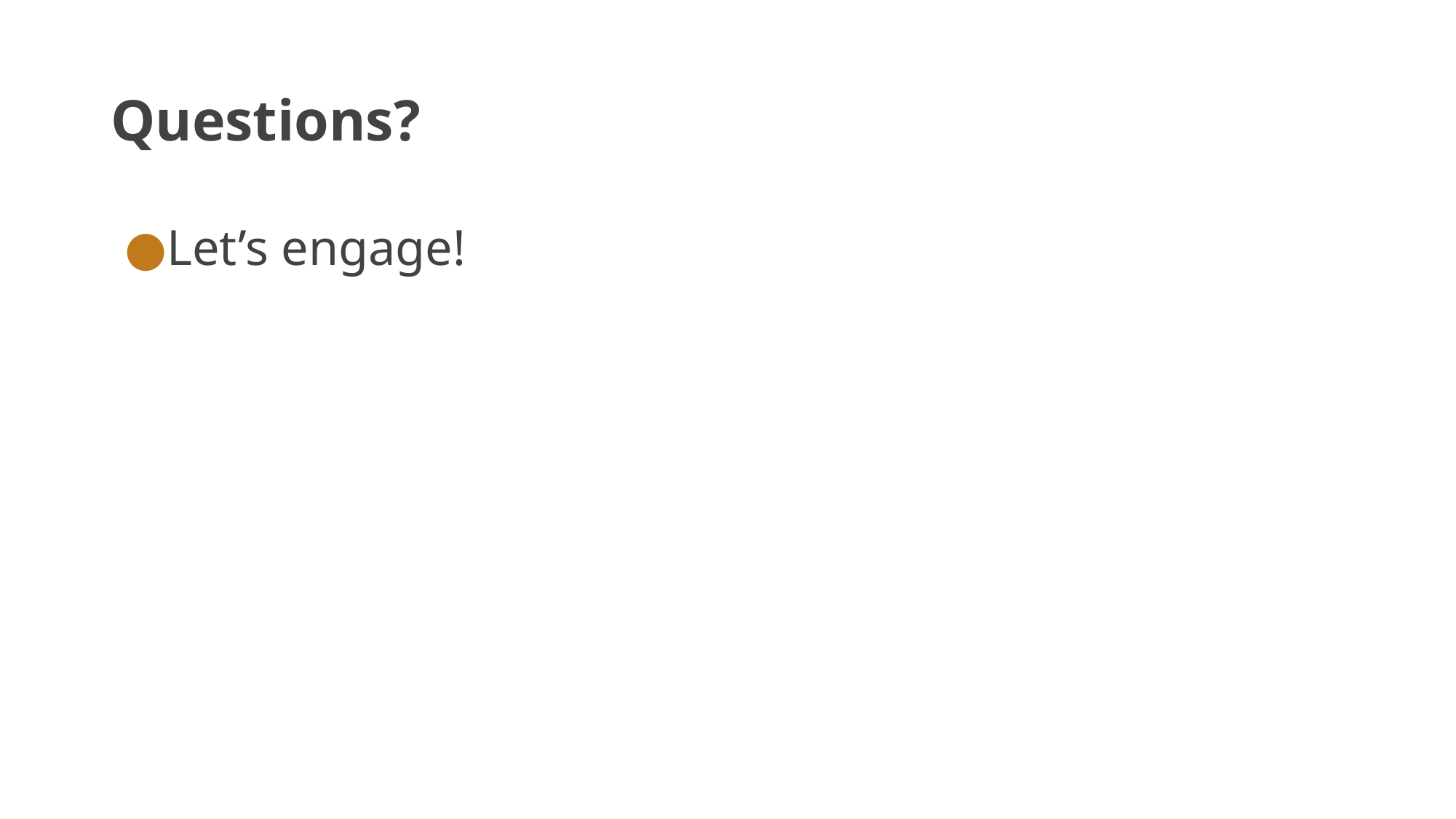

# Questions?
Let’s engage!
