# Supplementary material for: An Educational Session for Medical Students Exploring Weight Bias in Clinical Care Through the Lens of Body Diversity
Source: MedEdPORTAL. 2023 Sep 5;19:11342. doi: 10.15766/mep_2374-8265.11342 (PMC10477274; doi:10.15766/mep_2374-8265.11342)
Supplement: Supplementary file 1 — Understanding Body Diversity.pptxAddressing Weight Bias in Clinical Care.pptxFacilitator Guide.docxStudent Guide.docxMaterials Checklist and Timeline.docxQuiz.docxEvaluation Survey.docx [file mep_2374-8265.11342-s001.zip › E. Materials Checklist and Timeline.docx]

**Appendix E - Materials Checklist and Timeline**

**Weight Bias**

| **Lectures** | | | **Small Group** | |
| --- | --- | --- | --- | --- |
| **Materials Checklist** | | | **Materials Checklist** | |
| □ Lecture hall reservation | | | **□** Small group rooms reservation (10-12 students per room) with table and chairs | |
| □ Computer with PowerPoint | | | □ Facilitator Guide (Appendix C) x 1 | |
| □ Projector | | | □ Student Materials (Appendix D) x 1 per student | |
| □ Sign in sheets (if applicable) | | | □ Sign in sheets for each room (if applicable) | |
| **Timeline (50-60 minutes per lecture)** | | | **Timeline (95-120 minutes)** | |
| **Lecture #1: The Importance of Understanding Body Diversity** | | |  |  |
| **Section** | **Slide #** | **Time allotted** | **Section** | **Time allotted** |
| Introduction and objectives | 1-5 | 2 min | Intro and ground rules | 10 min |
| Historical review of weight bias | 6-11 | 10 min | Part 1 (Case Overview) | 10-15 min |
| Data and statistics | 12 | 2 min | Part 2 | 15-20 min |
| Problem with assumptions | 13-14 | 5 min | Part 3 | 15-20 min |
| Self-reflection | 15-16 | 5 min | Part 4 | 15-20 min |
| Understanding intersectionality | 17-20 | 10 min | Part 5 | 15 min |
| Changes / interventions we can make | 21-24 | 10 min | Case resolution and wrap up | 15-20 min |
| Summary | 25 | 1 min | **Total** | **95-120 min** |
| Questions |  | 5-10 min |  |  |
| **Total** |  | **55 min** |  |  |
| **Break** |  | **5-10 min** |  |  |
| **Lecture #2: Addressing Weight Bias in Clinical Care** | | |  |  |
| **Section** | **Slide #** | **Time allotted** |  |  |
| Intro and objectives | 1-4 | 1 min |  |  |
| Weight Centered Health Paradigm (WCHP) | 5-6 | 5 min |  |  |
| Factors influencing weight | 7 | 2 min |  |  |
| Limitations of BMI | 8-11 | 2 min |  |  |
| Harms of WCHP | 12 | 5 min |  |  |
| Pathologizing obesity | 13 | 2 min |  |  |
| Weight bias in healthcare | 14-20 | 10 min |  |  |
| Self-reflection | 21-22 | 2 min |  |  |
| Weight-inclusive care | 22-33 | 15 min |  |  |
| Summary | 34-35 | 1 min |  |  |
| Questions |  | 5-10 mins |  |  |
| **Total** |  | **55 min** |  |  |
| **Break** |  | **5-10 min** |  |  |
