# Supplementary material for: An Educational Session for Medical Students Exploring Weight Bias in Clinical Care Through the Lens of Body Diversity
Source: MedEdPORTAL. 2023 Sep 5;19:11342. doi: 10.15766/mep_2374-8265.11342 (PMC10477274; doi:10.15766/mep_2374-8265.11342)
Supplement: Supplementary file 1 — Understanding Body Diversity.pptxAddressing Weight Bias in Clinical Care.pptxFacilitator Guide.docxStudent Guide.docxMaterials Checklist and Timeline.docxQuiz.docxEvaluation Survey.docx [file mep_2374-8265.11342-s001.zip › _Educational Summary Report_11342.pdf]

MedEdPORTAL® | The AAMC Journal of Teaching and Learning Resources

Original Publication

Open Access

An Educational Session for Medical Students Exploring Weight Bias in Clinical Care Through the Lens of Body Diversity

Tzeidel Brown Eichenberg, MD, Seema Parikh, Joy Cox, PhD, Dhvani Doshi, MD, MPH, Mercedes Padilla-Register, MA, Michelle DallaPiazza, MD*

*Corresponding author: mld229@njms.rutgers.edu

Abstract

Introduction: Weight bias is pervasive in health care and can lead to inadequate care for people with higher weight. However, few medical schools offer training on mitigating weight bias and incorporating body diversity into clinical care. **Methods:** As part of a course for second-year medical students, we developed and implemented a 3-hour multimodal educational session on mitigating weight bias. Didactics included content on body diversity and addressing weight bias, followed by a facilitated case discussion in small groups focused on debunking common myths related to weight. Assessment consisted of an open-content quiz and evaluation of a postsession survey. We performed a thematic analysis of the essay quiz responses and evaluation survey results. **Results:** Three hundred fifty-three students participated in academic years 2020-2021 and 2021-2022. In the postsession quiz, students described several learning points, including understanding environmental influences on body size, improving communication by reducing weight bias, and strengthening the patient-provider relationship. In the postsession evaluation, students reported that their knowledge and skills had improved with respect to the learning objectives, with means of 4.0-4.1 on a 5-point Likert scale. Areas for suggested improvement included more time for discussion and more guidance on weight-inclusive care. **Discussion:** This multimodal educational session on weight bias was successful in meeting the stated learning objectives. Future work will consist of building on this content and extending future iterations to residents and attendings, with the goal of disrupting harmful assumptions and improving access to holistic, evidence-based care for all people, regardless of size.

Keywords
Body Acceptance, Body Diversity, Weight Bias, Weight Stigma, Weight-Inclusive Care, Case-Based Learning, Health Equity, Diversity, Equity, Inclusion

Educational Objectives

By the end of this activity, learners will be able to:

1. Discuss critiques of the weight-centered health paradigm.
2. Describe the multifactorial influences on body weight.
3. Describe the effect that weight bias can have on health care access, delivery, and outcomes.
4. Outline approaches to reduce weight stigma in the health care setting.
5. Discuss body diversity and body acceptance and their relevance to clinical care.

Citation:
Brown Eichenberg T, Parikh S, Cox J, Doshi D, Padilla-Register M, DallaPiazza M. An educational session for medical students exploring weight bias in clinical care through the lens of body diversity. *MedEdPORTAL*. 2023;19:11342.
https://doi.org/10.15766/mep_2374-8265.11342

Introduction

Weight bias is defined as negative weight-related attitudes, beliefs, and judgments regarding people with higher weight.[1] The source of this bias is the stigmatizing misconception that weight is a lifestyle choice and that higher weight results from personal failing. Weight bias is universal in Western society, leading to discrimination against people with higher weight in school, work, and health care. Given this, weight bias is a human rights and public health issue that must be addressed at multiple levels.[2]

Not only is achieving a "normal weight" often not feasible for people with higher weight, overemphasis on weight in the health care setting can cause harm in the form of missed diagnoses, delayed care, and amplified weight stigma.[3,4] Cisgender women disproportionately experience these detrimental effects.[2,5]

Copyright © 2023 Eichenberg et al. This is an open-access publication distributed under the terms of the Creative Commons Attribution license.

Cisgender women classified with "obesity" are four times more likely than patients with a "healthy" body mass index (BMI) to delay presenting to care for an acute concern[5] and are less likely to have on-time reproductive cancer screenings, reporting feelings of humiliation around being weighed and disrespectful treatment as barriers.[6] The experience of weight stigma, defined as the social devaluation of individuals with higher body weight,[2] is also associated with higher rates of depression, anxiety, and physiologic stress and can lead to binge eating and exercise avoidance.[4]

Despite ample evidence that weight bias in health care is harmful, most health care providers harbor weight bias.[3] At least a third of physicians endorse beliefs that people with higher weight are lazy, have less willpower or motivation, and are less likely than lower weight counterparts to successfully adhere to treatment plans.[7] Providers spend less time discussing patients' diagnosed health issues in favor of spending more time discussing diet and exercise during primary care visits for people with higher weight.[8] Providers both overestimate how frequently their patients with higher weight wish to discuss their weight and underestimate how motivated they feel to adopt health-modifying behaviors.[3,9]

The first study of weight bias among medical students, published in 1985, showed that they harbored universally negative attitudes toward "morbidly obese" patients.[10] A more recent analysis found that the majority of health professions students had witnessed instructors make derogatory weight-based remarks, and a third of the students surveyed held negative weight biases, which were associated with frustrated feelings toward treating patients with higher weight.[11]

Given the prevalence of weight bias among medical professionals, it is imperative that medical curricula include content on mitigating weight bias in clinical care. Research on weight bias education for health professions students has shown that utilizing simulation or demonstration is more effective at reducing weight bias than lecture-style instruction alone.[12,13] While one online lesson briefs clinicians on making their clinical space more welcoming to people with higher weight,[14] no medical education database has published lesson materials for a multimodal weight bias mitigation session. Two resources published in *MedEdPORTAL*[15,16] describe counseling patients on weight loss with sensitive, evidence-based techniques but primarily aim to encourage weight loss, as opposed to centering the students' awareness of their own weight bias, how to mitigate bias, and how to implement weight-inclusive clinical practice.

For the purpose of using nonstigmatizing language in this publication, we utilize the expression *higher weight* as a neutral term to describe body type and the word *obesity* to refer to the medicalized condition based on BMI assessment commonly used in clinical and research settings.[17] Here, we detail the content and educational outcomes of a multimodal workshop to educate medical students on the pervasive nature of weight bias in health care and how to begin to mitigate its harms. We incorporate new clinical paradigms acknowledging that all bodies are different and centering on physical, psychological, and general well-being, rather than body appearance. While we acknowledge that students will be exposed to a weight-centered health paradigm (WCHP) throughout their training, our goal in introducing a body diversity framework prior to clinical exposure is to provide multiple perspectives, reduce weight shaming, and encourage a weight-inclusive approach to health that promotes dignity, acceptance, and shared decision-making.

Methods

We integrated this 3-hour workshop into the required Health Equity and Social Justice (HESJ) course at Rutgers New Jersey Medical School. Prior to the weight bias session, students learned about implicit bias, racism and health, social determinants of health, LGBTQ+ health, and relationship-centered communication, among other topics.[18] Through the organ systems curriculum, students learned about the hormonal regulation of satiety and weight homeostasis, as well as weight loss treatments.

We first included a weight bias lecture in the academic year 2018-2019 (AY19); here, we report on outcomes from academic year 2020-2021 (AY21) and academic year 2021-2022 (AY22), when we updated the content to present it through a body diversity lens and with a small-group, case-based dialogue. In AY21, due to restrictions from the COVID-19 pandemic, the session was entirely remote, using recorded lectures and a video chat platform. In AY22, the session occurred in person.

Curriculum Design

We designed the workshop so that students could review the current literature on weight bias and body diversity through didactics and then engage in an interactive, case-based, small-group dialogue to concretize the information. Content and topics were developed after an in-depth literature review. In keeping with the structure of other topics in the HESJ curriculum, we included an interactive, case-based, small-group component applying adult learning practices.[19]

Copyright © 2023 Eichenberg et al. This is an open-access publication distributed under the terms of the Creative Commons Attribution license.

Didactic Lectures

Lecture 1: The Importance of Understanding Body Diversity (Appendix A): In AY21, given limitations from the COVID-19 pandemic, this lecture was recorded and assigned to students in advance of the small-group sessions. In AY22, this lecture was presented in person prior to the small group.

The lecture leveraged a community perspective and was developed and presented by a PhD faculty member with expertise in communications, social justice, and intersectional body diversity (Joy Cox). The lecture started by describing weight bias, its ubiquity in society, and the harm it causes. It then reviewed the history of cultural and social trends that evolved over time into oppression of people with higher weight, particularly how Western body ideals were shaped by racism and slavery. It reviewed key data on health care discrimination and linked these data to personal stories. The lecture concluded with the definitions and importance of body diversity and intersectionality, introducing body diversity best practices such as Health at Every Size (HAES),[20] body acceptance, intuitive eating, and empathic listening.

Lecture 2: Addressing Weight Bias in Clinical Care (Appendix B): In AY21, given limitations from the COVID-19 pandemic, this lecture was recorded and assigned to students in advance of the small-group sessions. The recorded lecture was again a preassignment in AY22, to be completed prior to the live lecture and the small group.

This lecture presented harms of weight bias within a clinical context. It was developed by physician faculty members for the HESJ course during AY19, then updated with a weight-inclusive lens in AY21 (Joy Cox, Michelle DallaPiazza). The lecture outlined critiques of the WCHP prevalent in public health and medicine, as well as how it oversimplifies the determinants of weight. We reviewed the multifaceted determinants of size, limitations of BMI as a health parameter, and the harms associated with the WCHP. Weight bias in health care was explored in depth, reviewing key research. We then highlighted strategies for mitigating personal bias and reducing barriers to timely and appropriate care, with a focus on the structural and communication approaches of weight-inclusive care. This portion emphasized viewing health as multifaceted and promoted improving health care access for people with higher weight and reducing weight stigma.[21] Screening for disordered eating was outlined. Frameworks such as HAES[20] were reviewed as evidence-based approaches to weight-inclusive care.

Small-Group Case Discussion

The small groups consisted of 12-13 students, led by one facilitator, for an allotted time of 95-120 minutes. The facilitators were faculty and residents from multiple specialties (internal medicine, pediatrics, surgery, emergency medicine, and family medicine). We supplied the small-group materials and instructions, including the facilitator guide (Appendix C), in advance to all facilitators during a training at the start of the course. We embedded prompting questions as well as explanatory information to provide context and guidance. The training consisted of reviewing the content of the lectures as well as discussing the case as presented in the facilitator guide. We asked facilitators to share their relevant personal and clinical experiences.

The small groups worked through an individual patient case with prompting questions. We provided the student materials (Appendix D) during, rather than in advance of, the session. The case explored the medical history and perspectives of a cisgender woman with HIV and multiple contributing factors to weight gain, leading her to experience weight bias during clinical care. Prompting questions focused on selected obesity myths,[21] asking students to reflect on how these myths could lead to inadequate or stigmatizing care. The myths included (1) weight can be controlled with diet and exercise alone, (2) people with higher weight lack motivation and self-control, and (3) forcing people to take responsibility for their weight is the best way to facilitate behavior change. The facilitators and prompting questions encouraged students to discuss how to best engage the patient, debunk these common myths, identify resources, and ensure nonstigmatizing care.

Appendix E provides a checklist for materials and timelines for the lectures and small-group session.

Assessment

Assessment for the session consisted of an open-content online quiz (Appendix F) testing content from the lectures and the small group. The quiz utilized both multiple-choice and short-essay response questions. Quiz scores were part of each student's course grade.

Evaluation

In AY21 and AY22, we asked students to complete an anonymous feedback survey after the session. The survey (Appendix G) asked them, using 5-point Likert scales, to rate each component of the content, to assess their confidence in achieving the stated learning objectives, and to rate whether they felt they had a deeper understanding of the role of body

Copyright © 2023 Eichenberg et al. This is an open-access publication distributed under the terms of the Creative Commons Attribution license.

diversity in clinical care. Students were also asked to submit free-text responses regarding strengths and improvements. The survey design was in keeping with standard surveys at our institution for curriculum evaluation. Using an online survey platform, the students submitted the evaluations within 1 week after the session.

Data Analysis

We obtained institutional IRB approval for analysis of the students' quiz and anonymous survey data. The course director (Michelle DallaPiazza) downloaded the essay quiz responses and permanently removed student identifiers. The essay questions included the following:

- Of the many factors that influence weight within the four main categories, which did you find to be most interesting or surprising and why?
- During the case discussion, which obesity myth discussion did you find to be most interesting and why?
- [Asked only in AY22] Reflecting on body acceptance and body diversity, how can incorporation of these concepts into the care of patients influence their health care experiences?

Two coders (Seema Parikh, Mercedes Padilla-Register) categorized by theme and independently coded the responses for the quiz and the free text from the evaluation survey using NVivo, a qualitative data analysis software, which provided frequency counts of each code. Student responses could contain multiple themes. Codes were created based on the common themes seen in the weight bias curriculum. At the conclusion of the coding, discrepancies between the two coders were discussed and solved by consensus. Coding discrepancies occurred less than 5% of the time. We also summarized the results of the responses on the anonymous evaluation survey, quantifying responses by frequency and calculating the mean for each category that had been assessed with a 5-point Likert scale.

Results

Student Demographics

In the 2 years that this mandatory weight bias session was offered, 353 students participated in the course. Fifty-one percent of the course participants identified as cisgender women, 49% as cisgender men, and none as transgender or nonbinary. Thirty-seven percent identified as Asian, 11% as Black/African American, 15% as Hispanic or Latino ethnicity, and 32% as White, while 5% chose not to report their race/ethnicity. Demographic makeup was similar across the 2 years the course was offered.

Analysis of the Quiz Responses

Results of the thematic analysis of the free-text quiz answers are shown in Tables 1 and 2. For both years, the frequency of themes was similar. Students in both years most often wrote that they were surprised by the impact of environmental factors on weight (Table 1). They wrote about how they had not previously thought about the effects of climate change, food access, and pop culture. The obesity myth that students most frequently found interesting to discuss was that weight can be controlled with diet and exercise alone (Table 1), highlighting the myriad factors that influence weight outside of diet and exercise. For the last question, asked only in AY22, students wrote about how incorporating body acceptance into clinical practice would improve health care outcomes (Table 2).

Analysis of the Evaluation Surveys

After the session, 261 participants (74%) completed an anonymous course evaluation survey, 129 (72%) from AY21 and 132 (75%) from AY22. They rated each component of the educational session on a 5-point Likert scale (1 = *Poor,* 2 = *Only Fair,* 3 = *Adequate,* 4 = *Good,* 5 = *Excellent*). Average responses from students in aggregate from both years were above 4 for all categories rating the course content quality, with the small group and the small-group facilitator rated highest (*M*s = 4.3 and 4.5, respectively). Mean responses were slightly higher in AY22 (in person) than in AY21 (virtual), on the order of 0.1-0.2 higher for each component.

Students also evaluated the degree to which their knowledge and/or skills improved in each of the five objectives after they had completed the weight bias course. They evaluated each objective on a scale from hardly at all (1) to a very high degree of agreement (5) that each objective had been met. The students largely felt that their knowledge and skills had improved with respect to the learning objectives (Figure), with most students reporting that they had improved to a considerable degree (4) or to a very high degree (5; *M* range = 4.0-4.1).

Sixty-nine percent of students agreed to a considerable (4) or a very high (5) degree that additional training on this topic would be beneficial to their training as a physician, and 78% of students overall agreed that they had a greater appreciation for body diversity after having completed the weight bias session (*M*s on 5-point Likert scale = 3.9 and 4.1, respectively).

In the two optional questions asking for feedback on the session's strengths and any suggested improvements (Table 3),

Copyright © 2023 Eichenberg et al. This is an open-access publication distributed under the terms of the Creative Commons Attribution license.

Table 1. Analysis of Quiz Responses (Influences on Weight and Myths) for AY21 ($N = 178$) and AY22 ($N = 175$)

Question and Themes[a]	No. (%)	
	AY21	AY22
Of the many factors that influence weight within the four main categories, which did you find to be most interesting or surprising and why?		
Environmental	94 (53)	117 (67)
Climate change	22 (12)	32 (18)
Media and pop culture	16 (9)	22 (13)
Access to food	19 (11)	22 (13)
Biological and medical	86 (48)	52 (30)
Genetics	15 (8)	16 (9)
Microbiome	28 (16)	15 (9)
Socioeconomic	75 (42)	61 (35)
Cost of food	20 (11)	7 (4)
Structural racism	17 (10)	15 (9)
Behavioral and cultural	34 (19)	24 (14)
Food traditions	18 (10)	15 (9)
Counterproductive weight loss dieting	5 (3)	3 (2)
During the case discussion, which myth discussion did you find to be most interesting and why?		
Anyone can control weight with diet and exercise alone	64 (36)	85 (49)
People with higher weight lack motivation and self-control	46 (26)	47 (27)
Forcing people to take responsibility for their weight is the best way to facilitate behavior change	33 (19)	30 (17)

Abbreviation: AY, academic year.
[a]Multiple themes could be counted in each response.

80 students (36 in AY21 and 44 in AY22) submitted comments. For the strengths, common themes included the interactive nature of the small-group discussion and the quality of the lectures. Students in AY21 more commonly highlighted the small-group activity, possibly because both lectures were presented asynchronously that year. Students in AY22 more frequently highlighted the in-person didactic lecture, The Importance of Understanding Body Diversity, specifically stating that the lecturer was "very engaging" and "her anecdotes made her presentation more powerful."

For improvements, students in both academic years suggested that lectures should include more on the practical uses of the weight bias curriculum, including strategies for talking to patients about weight and weight loss. Students also suggested that the curriculum could be bolstered with more patient-centered

Table 2. Analysis of Quiz Essay Responses for AY22 ($N = 175$)

Question and Themes	No. (%)	Example
Reflecting on body acceptance and body diversity, how can incorporation of these concepts into the care of patients influence their health care experiences?[a]		
Better care and outcomes	67 (39)	"It can also improve health outcomes because if the patient's opinions on their weight and body are included in the discussion of treatment options, they are more likely to follow through with treatment plans as well as return for things such as follow ups and future screenings."
Patient-provider relationship	38 (22)	"Setting this foundation paves the way for a healthy and productive patient-physician relationship that can be very effective when approaching other areas of concern."
Patient feeling comfortable	37 (21)	"Teaching more healthcare workers about body diversity is important to make these patients feel more comfortable sharing personal information with them."
Trust	35 (20)	"Incorporating body acceptance/diversity is the best way for providers to create a trusting relationship with the patient."
Better communication	34 (20)	"By reflecting on our own biases regarding weight and body habitus, we can improve our communication with patients by reducing harmful stigmatizing language."
Patient empowerment	14 (8)	"As physicians it is crucial to empower patients and not judge them based on implicit biases that we may have regarding weight."
Respect	9 (5)	"When a physician is aware of the weight bias, they are more likely to treat the patient with respect."

Abbreviation: AY, academic year.
[a]This question was asked only in AY22.

Copyright © 2023 Eichenberg et al. This is an open-access publication distributed under the terms of the Creative Commons Attribution license.

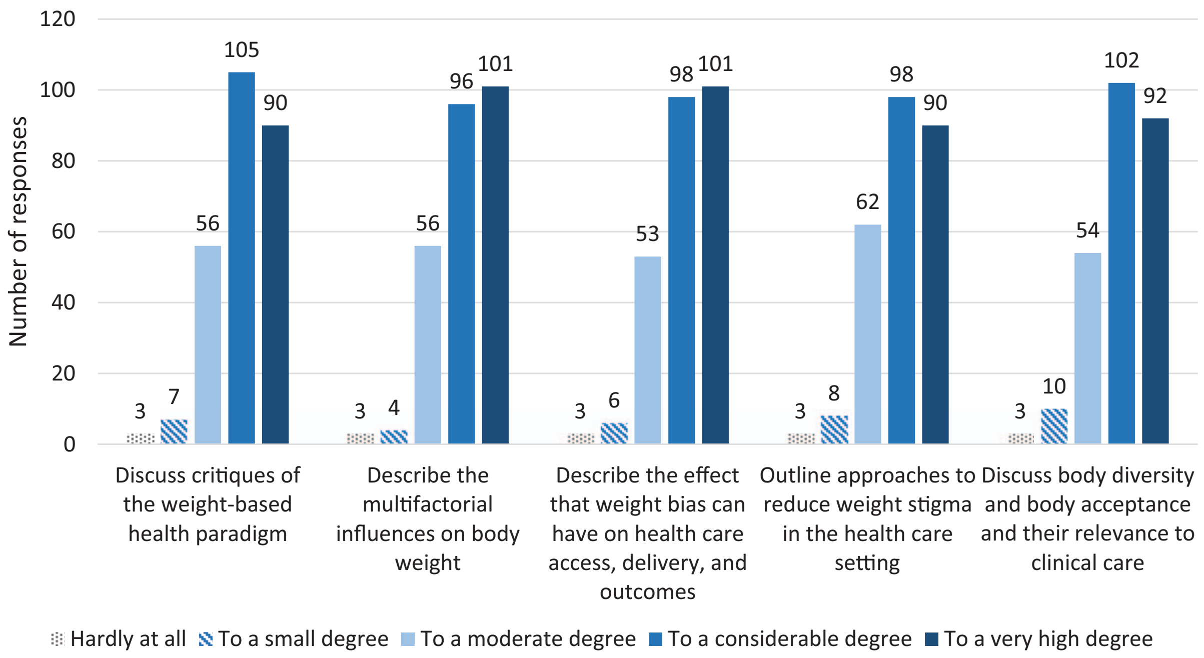

Figure. Student responses ($N = 261$) in academic years 2020-2021 and 2021-2022 indicating the degree to which knowledge and/or skills improved with respect to each of the five objectives in the evaluation survey. Responses left blank were not included in the total numbers.

examples and more time for discussion. A few of the suggestions strongly upheld the WCHP as an important consideration in improving health outcomes, citing lectures in the organ systems curriculum that underscored higher weight as a risk factor for disease.

Discussion

Among other interventions across media and public health, education of health care providers represents a key approach for minimizing weight stigma and its many pervasive harms.[2]

Table 3. Thematic Analysis of Student Evaluation Survey Comments on Strengths and Suggestions for Improvement

	No.		
**Theme**	**AY21**	**AY22**	**Example**
Strengths			
Small group with case study	18	12	"The case was really comprehensive and brought up a lot of interesting points that were conducive to a productive conversation."
Didactic lectures	7	28	"The lecture… was very well-done, engaging, and provided an interesting and new perspective on the issue of weight bias."
Improvements			
Attempt to limit technical difficulties	6	0	"There were some technical difficulties with [the virtual platform] at the beginning. Hopefully those can be resolved for future sessions."
Discuss more about the practical aspects, e.g., how to discuss how information can improve outcomes	4	6	"More tactical/practical training on how to engage with patients on this issue when there is a discrepancy between celebrating body diversity and dealing with medical concerns that involve weight."
More time for discussion/case	3	2	"Perhaps extended time allotted for questions and discussions."
Add in more information on obesity as a risk factor for disease	2	6	"I would have liked a bit more on weight as a risk factor for certain health conditions."
Lectures should be more evidence based	2	5	"I would have appreciated learning more about statistics and specific research involving the body diversity movement."
Supplement the curriculum with more patient experiences	1	6	"I think having more stories of how weight centered healthcare has had negative impacts on patients would further cement the importance of the activity."
More discussion on the myths, including how diet and exercise can help some control weight	1	2	"The small group was not inviting discussion when it asked to discuss a myth as though it was certainly false. There is nuance in the conversation. For many, exercise and diet are adequate to control weight, so why say that they aren't? This was not a discussion but an affirmation of predetermined facts which doesn't invite critical thinking."

Abbreviation: AY, academic year.

Copyright © 2023 Eichenberg et al. This is an open-access publication distributed under the terms of the Creative Commons Attribution license.

By using the lens of body diversity, this educational session for medical students aimed to dispel common myths and provide guidelines for reducing weight bias and stigma in clinical care. Students reported high levels of confidence in achieving the learning objectives in the postsession survey. Furthermore, they cited several important learning points: the importance of understanding the multifaceted influences on body size, the critical role of improving communication and care, and strengthening the patient-provider relationship with a body diversity approach.

Notably, this session incorporated elements not typically included in the medical curriculum, including an introduction to the concepts of body diversity and weight-inclusive care. As these concepts gain traction in both medical and social settings,[22] including social media, it is important for health care professionals to become familiar with the benefits of weight-inclusive care in decreasing weight stigma, enhancing patient-provider communication, improving health outcomes, and increasing access to high-quality clinical care.[23,24] Thus, it remains essential to explore the historical and social influences of weight bias, as well as the ways in which public health and health care have contributed to weight stigma.

Occasional feedback highlighted participants' concerns about how adoption of body acceptance approaches might minimize the impact that higher weight can have on clinical outcomes. In our experience, it has been challenging to navigate this tension, particularly since learners often come to this topic with strongly held beliefs and feelings. It is important for educators to acknowledge that students may view this session as presenting information contradictory to what they have previously learned in their medical school lectures regarding obesity as a disease and to lean into having this discussion in the small-group setting (see notes in Appendix C). Body diversity does not advocate for ignoring weight when it has clinical relevance but rather acknowledges the reality of different body shapes and sizes, centers well-being instead of achieving "normal weight," and encourages people to have a positive attitude toward their bodies so that they are able to care for them holistically over the long term.

Based on our feedback, it is helpful to underscore how body diversity and the systems curriculum can be complementary, as well as how we can recognize obesity as a risk factor for disease while also acknowledging and reducing the harms from our intense focus on weight as a direct reflection of health. As part of this, we plan to spend more time in future iterations reviewing how weight bias influences reduced acceptability and access to clinically indicated weight loss treatments at multiple levels—among patients who internalize weight stigma,[25,26] providers who lack knowledge about the multifactorial influences on body weight,[7] insurance companies that deny coverage for care,[27,28] and policymakers who influence guidelines promoting ineffective or non-evidence-based gatekeeping and limit resources.[29] We also plan to highlight how intense focus on the WCHP detracts from giving attention to social forces that have a demonstrable impact on well-being, such as readily accessible green space, high-quality education, social support, safe and affordable housing, and stable income.[30] Furthermore, future iterations will continue with in-person teaching, especially given students' preference and desire to explore guidance for weight-inclusive clinical care more thoroughly.

As understanding of these topics evolves and becomes more integrated into clinical spaces, care will need to be taken to ensure that the session's content remains relevant and tailored to the needs of medical professionals. This includes ensuring that the obesity myths explored in the small-group case discussion are still commonly held by medical students and that students are provided with resources on resisting the hidden curriculum and reporting or addressing instances of weight bias they may witness in their clinical rotations.

The major limitation of this analysis was its lack of ability to objectively assess learners' attitudes toward people with higher weight, as well as how these attitudes may have been affected by the curriculum. The postcurriculum assessment was designed to elicit learners' qualitative beliefs around their own understanding of the learning objectives and would have been strengthened by administration of a precurriculum survey for comparison. Ideally, this analysis would also have included an objective measure of learner weight bias, such as the Implicit Association Test[31] or an attitude-based questionnaire aimed at assessing bias, given prior to the curriculum as well as after. This would have enabled an assessment of a possible reduction in learner weight bias. Such an analysis would have been strengthened even further by an element of longitudinal analysis, such as a repeating of each class's measures of weight bias after significant clinical exposure, particularly at the end of third-year clinical rotations.

Lastly, like many conditions heavily influenced by social determinants and stigma, conveying the nuance and complexity of obesity and weight stigma research must extend beyond courses dedicated to health communication and health equity. Training on weight bias must also include residents and faculty

Copyright © 2023 Eichenberg et al. This is an open-access publication distributed under the terms of the Creative Commons Attribution license.

since students will be exposed to the WCHP throughout their immersive clinical experiences and within the hidden curriculum. We plan to continue to build on this content by reintroducing these concepts in the clerkships, and we are in the planning process for providing additional training sessions adapted to resident and faculty audiences. Given the pervasiveness of weight bias across society and health care, as well as the immense impact of weight stigma on health, it is essential that we train future physicians with the aim of promoting more inclusive, holistic, and evidence-based care for all people, regardless of size.

Appendices

A. Understanding Body Diversity.pptx
B. Addressing Weight Bias in Clinical Care.pptx
C. Facilitator Guide.docx
D. Student Guide.docx
E. Materials Checklist and Timeline.docx
F. Quiz.docx
G. Evaluation Survey.docx

All appendices are peer reviewed as integral parts of the Original Publication.

Tzeidel Brown Eichenberg, MD: Second-Year Resident, Department of Internal Medicine-Pediatrics, Rutgers New Jersey Medical School

Seema Parikh: Fourth-Year Medical Student, Rutgers New Jersey Medical School

Joy Cox, PhD: Program Development Analyst, Department of Education, Rutgers New Jersey Medical School

Dhvani Doshi, MD, MPH: Assistant Professor, Division of General Internal Medicine, Department of Medicine, Rutgers New Jersey Medical School

Mercedes Padilla-Register, MA: Program Supervisor, Office of Student Affairs, Rutgers New Jersey Medical School

Michelle DallaPiazza, MD: Associate Professor, Division of Infectious Diseases, Department of Medicine, Rutgers New Jersey Medical School; ORCID: https://orcid.org/0000-0003-3262-2368

Disclosures
None to report.

Funding/Support
None to report.

Ethical Approval
The Rutgers University Institutional Review Board approved this project.

References

1. Washington RL. Childhood obesity: issues of weight bias. *Prev Chronic Dis*. 2011;8(5):A94.
2. Rubino F, Puhl RM, Cummings DE, et al. Joint international consensus statement for ending stigma of obesity. *Nat Med*. 2020;26(4):485-497. https://doi.org/10.1038/s41591-020-0803-x
3. Puhl RM, Heuer CA. The stigma of obesity: a review and update. *Obesity (Silver Spring)*. 2009;17(5):941-964. https://doi.org/10.1038/oby.2008.636
4. Puhl RM, Heuer CA. Obesity stigma: important considerations for public health. *Am J Public Health*. 2010;100(6):1019-1028. https://doi.org/10.2105/AJPH.2009.159491
5. Olson CL, Schumaker HD, Yawn BP. Overweight women delay medical care. *Arch Fam Med*. 1994;3(10):888-892. https://doi.org/10.1001/archfami.3.10.888
6. Amy NK, Aalborg A, Lyons P, Keranen L. Barriers to routine gynecological cancer screening for White and African-American obese women. *Int J Obes (Lond)*. 2006;30(1):147-155. https://doi.org/10.1038/sj.ijo.0803105
7. Huizinga MM, Bleich SN, Beach MC, Clark JM, Cooper LA. Disparity in physician perception of patients' adherence to medications by obesity status. *Obesity (Silver Spring)*. 2010;18(10):1932-1937. https://doi.org/10.1038/oby.2010.35
8. Bertakis KD, Azari R. The impact of obesity on primary care visits. *Obes Res*. 2005;13(9):1615-1623. https://doi.org/10.1038/oby.2005.198
9. Befort CA, Greiner KA, Hall S, et al. Weight-related perceptions among patients and physicians. *J Gen Intern Med*. 2006;21(10):1086-1090. https://doi.org/10.1111/j.1525-1497.2006.00567.x
10. Blumberg P, Mellis LP. Medical students' attitudes toward the obese and the morbidly obese. *Int J Eat Disord*. 1985;4(2):169-175. https://doi.org/10.1002/1098-108X(198505)4:2<169::AID-EAT2260040204>3.0.CO;2-F
11. Puhl RM, Luedicke J, Grilo CM. Obesity bias in training: attitudes, beliefs, and observations among advanced trainees in professional health disciplines. *Obesity (Silver Spring)*. 2014;22(4):1008-1015. https://doi.org/10.1002/oby.20637
12. Wiese HJ, Wilson JF, Jones RA, Neises M. Obesity stigma reduction in medical students. *Int J Obes Relat Metab Disord*. 1992;16(11):859-868.
13. Matharu K, Shapiro JF, Hammer RR, Kravitz RL, Wilson MD, Fitzgerald FT. Reducing obesity prejudice in medical education. *Educ Health (Abingdon)*. 2014;27(3):231-237. https://doi.org/10.4103/1357-6283.152176

Copyright © 2023 Eichenberg et al. This is an open-access publication distributed under the terms of the Creative Commons Attribution license.

14. Ginsburg BM. Destigmatizing obesity and overcoming inherent barriers to obtain improved patient engagement. StatPearls. Updated January 1, 2023. Accessed July 26, 2023. https://www.statpearls.com/nurse/ce/activity/74416
15. Miller DP, Crandall SJ, Davis S, et al. NEW LifeStyle: a downloadable teaching and learning program for nutrition, exercise, and weight management. *MedEdPORTAL*. 2012;8:9256. https://doi.org/10.15766/mep_2374-8265.9256
16. Wilechansky R, Burgermaster M, Jones D, Seres D. Obesity, diet, and exercise education for the primary care clerkship using an Articulate Storyline 2 e-learning module. *MedEdPORTAL*. 2016;12:10497. https://doi.org/10.15766/mep_2374-8265.10497
17. Puhl RM. What words should we use to talk about weight? A systematic review of quantitative and qualitative studies examining preferences for weight-related terminology. *Obes Rev*. 2020;21(6):e13008. https://doi.org/10.1111/obr.13008
18. DallaPiazza M, Ayyala MS, Soto-Greene ML. Empowering future physicians to advocate for health equity: a blueprint for a longitudinal thread in undergraduate medical education. *Med Teach*. 2020;42(7):806-812. https://doi.org/10.1080/0142159X.2020.1737322
19. Reed S, Shell R, Kassis K, et al. Applying adult learning practices in medical education. *Curr Probl Pediatr Adolesc Health Care*. 2014;44(6):170-181. https://doi.org/10.1016/j.cppeds.2014.01.008
20. Health at Every Size Principles: about Health at Every Size (HAES). Association for Size Diversity and Health. Accessed July 26, 2023. https://asdah.org/health-at-every-size-haes-approach
21. Ramos Salas X, Forhan M, Sharma AM. Diffusing obesity myths. *Clin Obes*. 2014;4(3):189-196. https://doi.org/10.1111/cob.12059
22. Harper K. The weight of stigma: heavier patients confront the burden of bias. *Scientific American*. May 19, 2022. Accessed July 26, 2023. https://www.scientificamerican.com/article/the-weight-of-stigma-heavier-patients-confront-the-burden-of-bias1/
23. Tylka TL, Annunziato RA, Burgard D, et al. The weight-inclusive versus weight-normative approach to health: evaluating the evidence for prioritizing well-being over weight loss. *J Obes*. 2014;2014:983495. https://doi.org/10.1155/2014/983495
24. Bacon L, Aphramor L. Weight science: evaluating the evidence for a paradigm shift. *Nutr J*. 2011;10:9. https://doi.org/10.1186/1475-2891-10-9
25. Verhaak AMS, Ferrand J, Puhl RM, Tishler DS, Papasavas PK, Umashanker D. Experienced weight stigma, internalized weight bias, and clinical attrition in a medical weight loss patient sample. *Int J Obes (Lond)*. 2022;46(6):1241-1243. https://doi.org/10.1038/s41366-022-01087-2
26. Kaplan LM, Golden A, Jinnett K, et al. Perceptions of barriers to effective obesity care: results from the national ACTION study. *Obesity (Silver Spring)*. 2018;26(1):61-69. https://doi.org/10.1002/oby.22054
27. Obesity drugs: few adults used prescription drugs for weight loss and insurance coverage varied. U.S. Government Accountability Office. August 9, 2019. Accessed July 26, 2023. https://www.gao.gov/products/gao-19-577
28. Gebran SG, Knighton B, Ngaage LM, et al. Insurance coverage criteria for bariatric surgery: a survey of policies. *Obes Surg*. 2020;30(2):707-713. https://doi.org/10.1007/s11695-019-04243-2
29. Gomez G, Stanford FC. US health policy and prescription drug coverage of FDA-approved medications for the treatment of obesity. *Int J Obes (Lond)*. 2018;42(3):495-500. https://doi.org/10.1038/ijo.2017.287
30. Silventoinen K, Rokholm B, Kaprio J, Sørensen TIA. The genetic and environmental influences on childhood obesity: a systematic review of twin and adoption studies. *Int J Obes (Lond)*. 2010;34(1):29-40. https://doi.org/10.1038/ijo.2009.177
31. Greenwald T, Banaji M, Nosek B, Teachman B, Nock M. Implicit Association Test. Project Implicit. 2011. Accessed July 26, 2023. https://implicit.harvard.edu/implicit/

Received: February 14, 2023
Accepted: June 8, 2023
Published: September 5, 2023

Copyright © 2023 Eichenberg et al. This is an open-access publication distributed under the terms of the Creative Commons Attribution license.
